# Land in limbo: Nearly one third of Indonesia’s cleared old-growth forests left idle

**DOI:** 10.1073/pnas.2318029121

**Published:** 2024-07-01

**Authors:** Diana Parker, Anna Tosiani, Muhammad Yazid, Inggit L. Sari, Tatik Kartika, Kustiyo, Rizky Firmansyah, Zuraidah Said, Arief Wijaya, Peter Potapov, Alexandra Tyukavina, Stephen V. Stehman, Viviana Zalles, Amy Pickens, Jeffrey Pickering, Svetlana Turubanova, Matthew C. Hansen

**Affiliations:** ^a^Department of Geographical Sciences, University of Maryland, College Park, MD 20742; ^b^Ministry of Environment and Forestry of Indonesia, Jakarta 10270, Indonesia; ^c^National Research and Innovation Agency of Indonesia, Jakarta 10340, Indonesia; ^d^World Resources Institute of Indonesia, Jakarta 12170, Indonesia; ^e^United Nations Development Programme Indonesia, Jakarta 10250, Indonesia; ^f^Department of Sustainable Resources Management, College of Environmental Science and Forestry, State University of New York, Syracuse, NY 13210

**Keywords:** land use change, deforestation, palm oil, carbon emissions

## Abstract

Indonesia has experienced rapid primary forest loss, second only to Brazil in modern history. We examined the fates of Indonesian deforested areas, immediately after clearing and over time, to quantify deforestation drivers in Indonesia. Using time-series satellite data, we tracked degradation and clearing events in intact and degraded natural forests from 1991 to 2020, as well as land use trajectories after forest loss. While an estimated 7.8 Mha (SE = 0.4) of forest cleared during this period had been planted with oil palms by 2020, another 8.8 Mha (SE = 0.4) remained unused. Of the 28.4 Mha (SE = 0.7) deforested, over half were either initially left idle or experienced crop failure before a land use could be detected, and 44% remained unused for 5 y or more. A majority (54%) of these areas were cleared mechanically (not by escaped fires), and in cases where idle lands were eventually converted to productive uses, oil palm plantations were by far the most common outcome. The apparent deliberate creation of idle deforested land in Indonesia and subsequent conversion of idle areas to oil palm plantations indicates that speculation and land banking for palm oil substantially contribute to forest loss, although failed plantations could also contribute to this dynamic. We also found that in Sumatra, few lowland forests remained, suggesting that a lack of remaining forest appropriate for palm oil production, together with an extensive area of banked deforested land, may partially explain slowing forest loss in Indonesia in recent years.

## Significance

Indonesia has lost 25% of its old-growth forest since 1990, with its intact forest area (natural forest undisturbed by human activity) declining by 45%. Nearly half (44%) of Indonesia’s deforested land had no detectable land use for 5+ y after clearing. This was caused by fires, long assumed to be Indonesia’s principal idle land driver, and by deliberate mechanical clearing, an understudied phenomenon despite its large deforestation footprint. When idle areas were converted to productive uses, the majority were planted with oil palms, which covered 28% of Indonesia’s deforested land by 2020. Oil palms were the only major land use for which lagged conversion was the norm; other major drivers such as smallholder agriculture were typically established immediately after clearing.

Of the three countries with the largest tropical forest areas—Brazil, Democratic Republic of Congo, and Indonesia—Indonesia has the least remaining primary forest and, until recently, was experiencing the highest deforestation rate proportional to its total forest area (1). This rapid deforestation has contributed to Indonesia’s position as one of the world’s top greenhouse gas emitters (2). This is due in part to Indonesia’s extensive peatland forests, which contain much higher levels of below-ground biomass compared to forests on dry soils. When peatlands are drained and degraded or cleared, fires can occur and smolder for weeks or months (3–5), releasing massive amounts of carbon and posing serious public health risks. This, together with the sheer extent of Indonesia’s forest loss over the past few decades, has left the country facing scrutiny from civil society groups and trading partners, with its palm oil industry facing particular pressure to stop clearing primary forest (6).

Indonesia is the world’s largest producer of palm oil and oil palm plantations have expanded in areas previously covered by natural forests (6–13). Measuring the extent to which palm oil causes deforestation, however, is complicated by the transitional nature of Indonesian land use change. Indonesia contains extensive nonforest areas not planted with commodity crops, sometimes referred to as “degraded” or “underutilized” lands (6, 8, 13, 14). Academic researchers and environmental advocates have suggested that oil palm expansion in these landscapes, rather than in primary forests, could help improve sustainability in the industry (6, 8, 9, 14–17). Rehabilitating underutilized landscapes is also a major component of Indonesia’s goal to make its forest and land sector a net carbon sink by 2050, despite plans to continue clearing primary forest over that period (18). However, little attention has been paid to the creation of these underutilized areas, many of which were previously forested.

We examined the fates of cleared primary forests, immediately after clearing and over time, to track deforestation drivers in Indonesia over a 30-y period (1991 to 2020). To determine the extent to which various drivers contributed to primary forest conversion, we used a probability sample to estimate the expansion of both economically productive land uses (such as oil palm) in previously forested areas, and the creation of seemingly idle (“underutilized”) lands. Within idle areas, we differentiated between forest fire clearing and mechanically cleared unplanted land (land left unused, without evidence of crop planting, following manual or mechanized tree felling). This approach allowed us to estimate the area of idle land created by fire, which could have been either intentionally or accidentally set, or cleared mechanically, which shows definitive intent. By tracking postdeforestation changes, we also estimated the area of idle land later converted to economically productive uses. Last, we estimated the area of intact primary forest—old growth forest not logged or otherwise disturbed in recent history—that was degraded during the study period by tracking forest disturbance events such as selective logging.

## Results

**Fig. 1.**
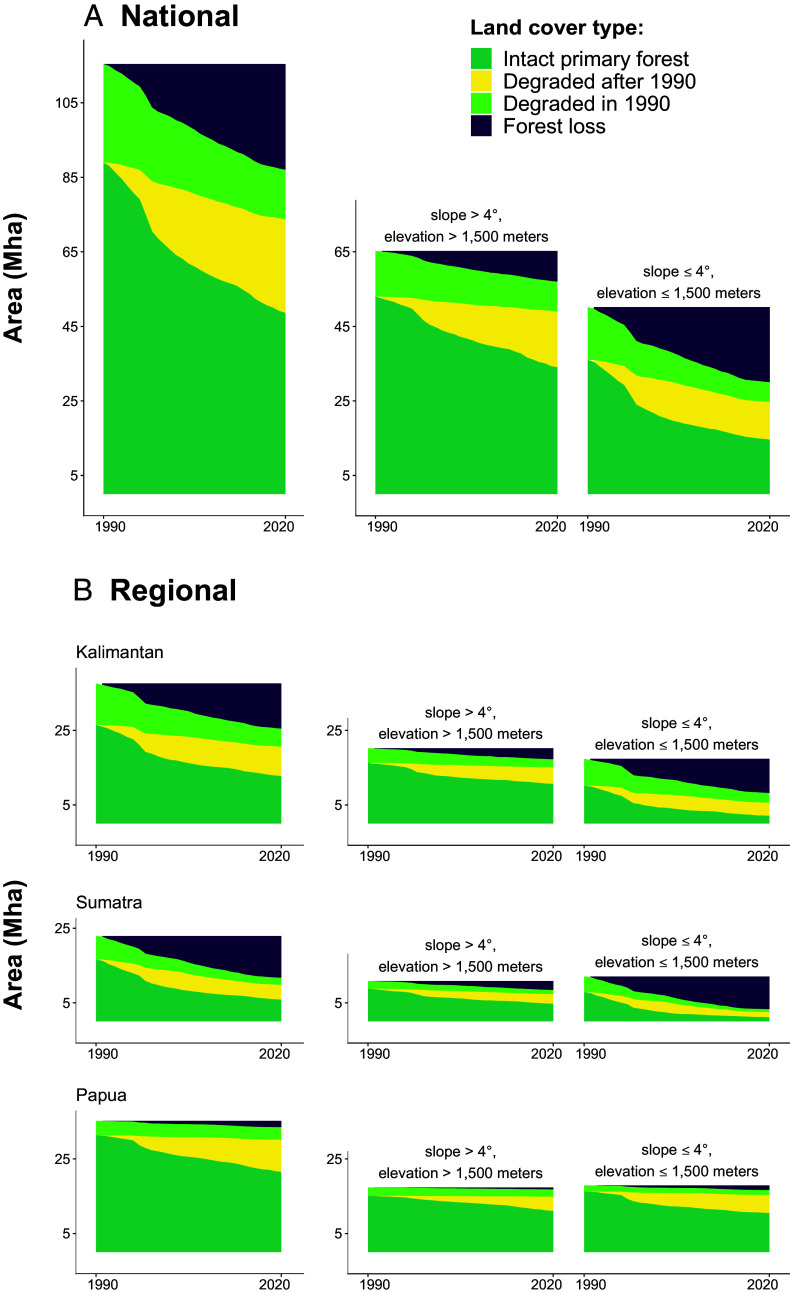
Estimated areas (Mha) of intact and degraded primary forest and forest loss nationally (*A*) and regionally (*B*) from 1990 to 2020. Includes all 1990 primary forest landscapes (column 1) and landscapes with steep slopes and/or high elevations (>4°/>1,500 m) and lower slopes/elevations (≤4°/≤1,500 m) (columns 2 & 3, respectively).

From 1991 to 2020, Indonesia lost an estimated 28.4 (SE of ±0.7) million hectares (Mha) of primary forest, an area roughly 50 times larger than the island of Bali. This represents a loss of nearly one quarter (24.6% ± 0.5) of the total primary forest area in 1990 (Fig. 1). An additional 25.1 (±0.6) Mha of intact primary forest was degraded but not cleared, leaving just 48.6 (±0.8) Mha of intact primary forest remaining in 2020 (*SI Appendix*, Table S1).

Nearly half (44.1% ± 1.3) of all deforested land remained idle for 5 y or more after clearing, and a majority (56.0% ± 1.3) was idle for at least 1 y (Table 1). In these areas, forests were not replaced with crops, used for shifting cultivation (including fallows), or converted to any other observable land use. We will refer to these idle areas, defined as land not converted to an observable land use for at least 12 mo after forest loss, as “unproductive,” although they may still be used in indirect ways, for example as a source of income through land sales, a way to attract investors, or as part of a company’s land bank (19).

**Table 1.** Estimated area and percent of cleared forests converted to land uses at different postdeforestation time steps

	Area estimate (Mha)				Percent of all forest loss (28.37 Mha)			
	Directly after loss	5+ y after loss (≤2015)	5+ y after loss (all loss)	2020	Directly after loss	5+ y after loss (≤2015)	5+ y after loss (all loss)	2020
Total unproductive clearing	15.89 (±0.51)	12.51 (±0.46)	13.34 (±0.48)	8.78 (±0.39)	56% (±1.3)	44.1% (±1.3)	47% (±1.3)	30.9% (±1.2)
Mechanically cleared	8.53 (±0.39)	5.62 (±0.32)	6.27 (±0.34)	3.98 (±0.27)	30.1% (±1.2)	19.8% (±1)	22.1% (±1.1)	14% (±0.9)
Cleared by fire	7.36 (±0.36)	6.89 (±0.35)	7.08 (±0.35)	4.79 (±0.3)	25.9% (±1.1)	24.3% (±1.1)	24.9% (±1.1)	16.9% (±1)
Total productive clearing	12.18 (±0.46)	13.52 (±0.48)	14.59 (±0.5)	17.8 (±0.54)	42.9% (±1.3)	47.6% (±1.3)	51.4% (±1.3)	62.7% (±1.2)
Oil palm	2.6 (±0.22)	4.4 (±0.28)	4.59 (±0.29)	7.83 (±0.37)	9.2% (±0.7)	15.5% (±0.9)	16.2% (±0.9)	27.6% (±1.2)
Smallholder land use	5.16 (±0.31)	4.63 (±0.29)	5.17 (±0.31)	5.08 (±0.3)	18.2% (±1)	16.3% (±0.9)	18.2% (±1)	17.9% (±1)
Tree plantations	1.74 (±0.18)	1.87 (±0.19)	1.92 (±0.19)	1.83 (±0.18)	6.1% (±0.6)	6.6% (±0.6)	6.8% (±0.6)	6.5% (±0.6)
Other land use	1.85 (±0.19)	1.92 (±0.19)	2.11 (±0.2)	2.26 (±0.2)	6.5% (±0.6)	6.8% (±0.6)	7.5% (±0.7)	8% (±0.7)
Built up land	0.83 (±0.12)	0.7 (±0.11)	0.79 (±0.12)	0.79 (±0.12)	2.9% (±0.4)	2.5% (±0.4)	2.8% (±0.4)	2.8% (±0.4)
Land use unclear	0.3 (±0.08)	0.13 (±0.05)	0.43 (±0.09)	1.79 (±0.18)	1.1% (±0.3)	0.5% (±0.2)	1.5% (±0.3)	6.3% (±0.6)

Time steps include: directly after loss (within 12 mo of forest clearing), 5+ y after loss (excluding areas cleared after 2015), 5+ y after loss (including all loss years), and in 2020. For areas cleared after 2015, the land use in 2020 was used to calculate the 5+ y after loss estimates. SEs reported in parentheses.

### Fire.

Of the 15.9 (±0.5) Mha of cleared forest left unproductive for at least 12 mo, 7.4 (±0.4) Mha was deforested by fire. Fire is both a driver of forest loss in Indonesia and a tool to clear debris after tree felling (20–23). We defined deforestation by fire as the loss of at least 50% of the natural canopy from fire in a standing forest, with land subsequently left idle for 1 y or more. Debris burning after mechanical clearing and burned areas converted immediately (within 12 mo) to an observable land use were not classified as fire clearing.

Deforestation by fire accounted for an estimated 25.9% (±1.1) of all primary forest loss from 1991 to 2020, and just under half (46.3% ± 1.7) of all unproductive clearing. Forest loss due to fire occurred almost exclusively during El Niño events, with an estimated 3.5 (±0.3) Mha of primary forest cleared by fire during the 1997/98 El Niño alone (*SI Appendix*, Fig. S1).

Areas deforested by fire were often left idle for long periods of time. An estimated 92.1% (±1.4) of all forests lost due to fire had not been converted to a productive use 5 y after the initial clearing event, and 4.8 (±0.3) Mha of idle land deforested by fire existed in Indonesia in 2020.

### Mechanically Cleared Unplanted Land.

**Fig. 2.**
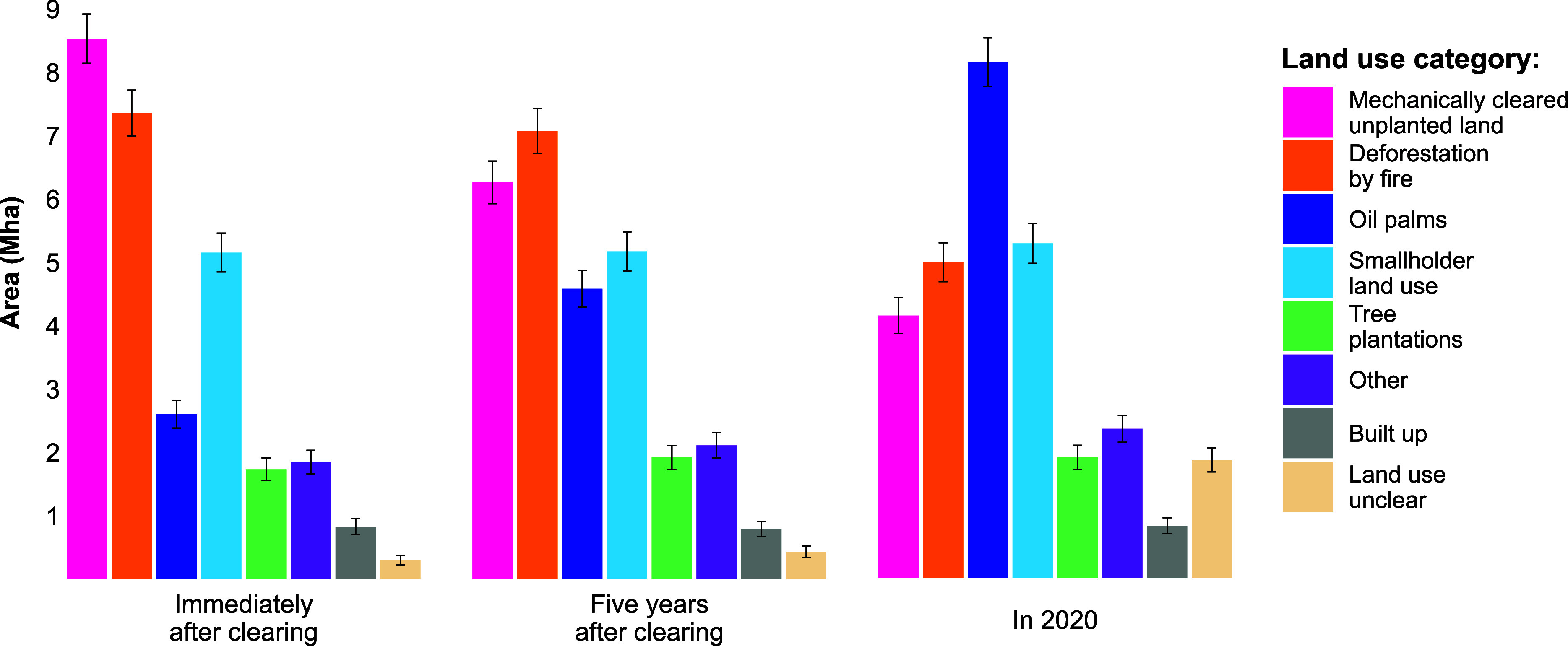
Land use areas reported for three time-steps: immediately (within 12 mo) after forest clearing, 5 y after forest clearing (or, for land cleared after 2015, at the end of the study period), and in 2020.

While fire was a major deforestation driver in Indonesia, an even larger unproductive area (8.5 ± 0.4 Mha) was mechanically cleared. In some of these cases, fire may have been used to clear debris after tree felling but was not the forest clearing mechanism (*SI Appendix*, Fig. S2). These mechanically cleared unplanted areas represent 30.1% (±1.2) of all primary forest loss in Indonesia, more than any other driver (Fig. 2).

**Fig. 3.**
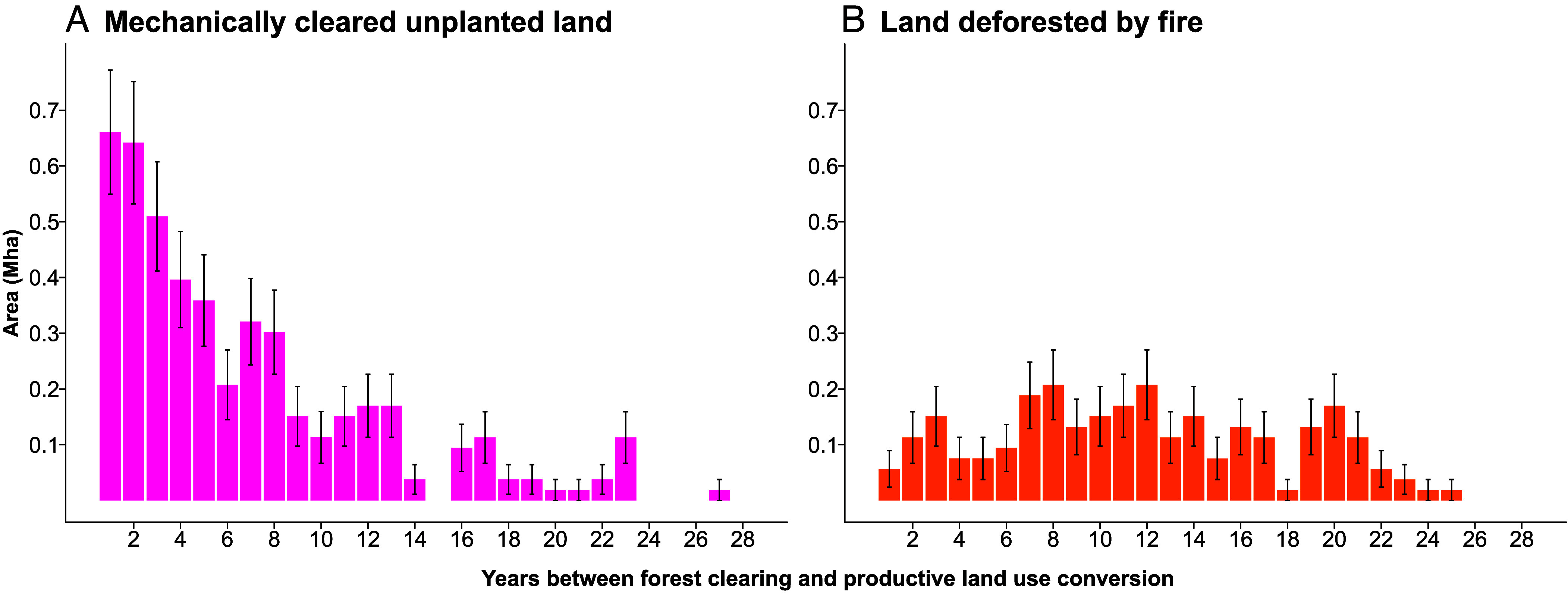
Time between forest clearing and productive land use establishment in areas that were initially left idle. Idle land is disaggregated by clearing mechanism: mechanically cleared land (*A*) and land deforested by fire (*B*).

Compared to fire-cleared forests, mechanically cleared unplanted land was more rapidly converted to productive uses, although 4.0 (±0.3) Mha remained idle in 2020. Five years after clearing (or, for areas cleared after 2015, at the end of the study period), productive land uses had been established in 25.7% (±2.1) of mechanically cleared unplanted areas, and 49.3% (±2.3) were converted to a productive use by 2020. Mechanically cleared unproductive areas that became productive after a lag experienced a median idle period of 6 y, compared to 12 y in productive areas initially cleared by fire (Fig. 3).

These findings contradict the common assumption that most idle landscapes were either created by fire or were shifting cultivation fallows (13, 24). Fire did play an important role but was responsible for less than half of all unproductive deforested land. Additionally, the trajectories following forest loss show that plantation companies and others expanding into idle areas are less likely to use fire-cleared land. Shifting cultivation fallows were classified as smallholder land use and excluded from our idle land class; newly deforested (after 1990) shifting cultivation land was also seldomly converted to other uses (*SI Appendix*, Table S2).

### Productive Land Uses.

By 2020, oil palm plantations covered 7.8 (±0.4) Mha of previously forested land, more than any other productive land use. Of this area, 32.5% (±2.3) immediately replaced primary forests, meaning oil palms were planted within 12 mo of forest clearing. Another 43.6% (±2.4) had been planted in mechanically cleared idle areas after a delay of 1 y or more, and 19.5% (±1.9) expanded into areas initially cleared by fire; 4.3% (±1) replaced other productive land uses (*SI Appendix*, Table S2).

Between 1991 and 2020, 6.0 (±0.3) Mha of primary forest was either cleared and immediately replaced with oil palms or was mechanically cleared, left idle, and then planted with oil palms after a lag. Other productive land uses were less frequently established in idle areas; the lagged conversion dynamic was most common in areas used for palm oil production.

**Fig. 4.**
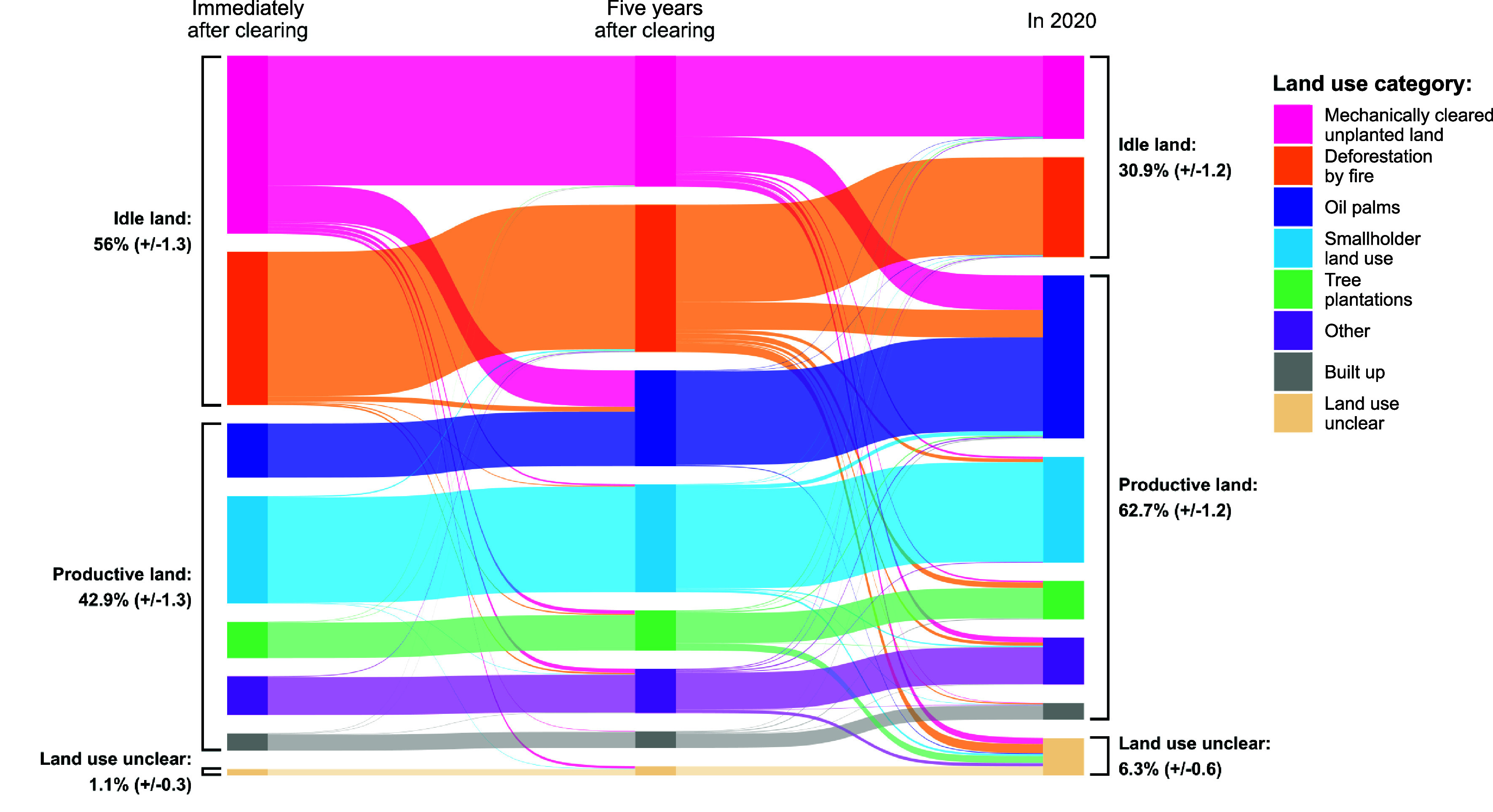
Land use transitions expressed as proportions of the total area of deforestation. Time steps include immediately after clearing (within 12 mo), 5 y after clearing (or, for land cleared after 2015, at the end of the study period), and 2020.

Fig. 4 illustrates this trend. Immediately after clearing, smallholder land use (including both permanent and shifting cultivation agriculture, and excluding small-scale oil palm), was the largest productive land use in previously forested areas, covering 5.2 (±0.3) Mha. Oil palms (including large- and small-scale plantations) covered 2.6 (±0.2) Mha. Five years after forest clearing, the area planted with oil palms had grown to 4.6 (±0.3) Mha, a 76% increase. By the end of the study period, the deforested area used for smallholder agriculture was 5.1 (±0.3) Mha, a slight decrease compared to the initial smallholder area. Other productive drivers also experienced relatively minor changes. The area planted with oil palms, meanwhile, had expanded to cover more than one quarter (27.6% ± 1.2) of Indonesia’s deforested land.

### Regional Patterns.

An estimated 42.7% (±1.1) and 39.4% (±1.1) of all forest loss occurred in Kalimantan and Sumatra, respectively. These regions also had the highest relative rates of deforestation observed during the study period. By 2020, Sumatra had lost nearly half of its 1990 primary forest area (48.8% ± 1.4), and Kalimantan had lost roughly one-third (32.2% ± 1) (Fig. 1). Deforestation was more common in areas with low slopes (≤4°) and elevations not exceeding 1,500 m above sea level—the landscapes most ideal for oil palms and typically easiest to access (25). An estimated 71% (±1.2) of all deforestation observed during the study period occurred in these low-slope/low-elevation areas, despite the fact that those landscapes contained less than half (43.5% ± 0.6) of Indonesia’s 1990 primary forest. Nationally, Indonesia lost 40.1% (±0.9) of its primary forest in areas with low slopes and elevations, while Sumatra and Kalimantan lost 72.8% (±1.8) and 52.8% (±1.6), respectively. By 2020, only 3.3 (±0.2) Mha of low-slope forested land remained in Sumatra, while 8.8 (±0.4) Mha had been cleared.

### Intact Forest Degradation.

Most deforested areas (77.4%±1.1) were degraded at least 1 y before clearing. For this study, we define forest degradation as a disturbance at a 30-m resolution or within a 140-m radius resulting in <50% canopy loss at a 30-m resolution. Common forest degradation causes include selective logging, nearby clearing, and non-stand-clearing fires (*SI Appendix*, Fig. S3).

Degraded primary forests were more vulnerable to conversion compared to intact areas. Of the estimated 26.4 (±0.6) Mha of forest already degraded in 1990, 49.7% (±1.3) had been cleared by 2020, compared to just 17.1% (±0.5) of forests that were intact in 1990 (*SI Appendix*, Fig. S4). Intact areas degraded after 1990 were also vulnerable to clearing; of the 35.6 (±0.7) Mha of intact primary forest degraded during the study period, 29.4% (±1) had been cleared by 2020. In areas with slopes ≤4° and elevations ≤ 1,500 m, nearly half (43%±1.6) of all intact forests degraded after 1990 were eventually deforested.

### Recent Deforestation Declines.

From 2017 to 2020, Indonesia experienced the lowest primary forest loss rates observed during the study period (*SI Appendix*, Fig. S1). The average annual deforestation from 2017 to 2020 was just 0.36 (±0.04) Mha, a 56% decrease compared to the previous 4 y and 62% lower than the mean annual loss over the entire study period. If fire clearing, which is heavily influenced by weather conditions, is excluded, deforestation from 2017 to 2020 declined by 48% compared to the previous 4 y (*SI Appendix*, Fig. S5).

Intact primary forest degradation also declined in 2017 to 2020, dropping 38% compared to the previous 4-y period, and 50% in low slope/low elevation landscapes (*SI Appendix*, Table S3). Given that degraded primary forests are more vulnerable to clearing, a decline in intact forest degradation may be a hopeful sign that deforestation will remain low. At the same time, the relatively high rates of intact forest degradation in Papua may mean forests there are vulnerable to future clearing, despite the region’s historically low deforestation rates.

## Discussion

The extent to which Indonesian primary forests are being actively cleared then left idle has not been well documented. Our study shows that the majority of idle lands created after 1990 were not caused by escaped fires but were the result of deliberate mechanical clearing. When deforestation was caused by fire, it typically coincided with El Niño events, with nearly half (46.9% ± 2.5) of all forest fire clearing occurring during the 1997/98 El Niño alone. Together, mechanically cleared unplanted land and deforestation by fire accounted for more than half of all primary forest loss in Indonesia. Recent pantropical research has found that the extent of deforestation in agricultural frontiers is much larger than the area actually converted to agricultural land uses (26). Our research supports this finding, and the land use trajectories we observe provide insight into why this phenomenon may be occurring.

Previous studies have found that oil palm plantations are often established in idle deforested land, including a substantial area planted many years after forest loss (8). We build upon this research by identifying the deforestation mechanism in idle areas and tracking the differing trajectories in areas cleared mechanically compared to those cleared by fire. The postclearing trajectories of mechanically cleared unplanted areas suggest a strong link between intentional idle land creation and oil palm expansion. Social science literature supports this connection and suggests that land speculation for oil palm may be a major idle-land driver. Land banking and land speculation are common in Indonesia, and plantations can remain undeveloped for years or even decades after concessions are issued (16, 17, 19). Forest clearing, however, often occurs much more rapidly, and palm oil license holders have been observed clearing primary forest and then leaving the land idle for years (16, 17). The extent to which industrial idle land creation is intentional or the result of unforeseen circumstances cannot be determined through satellite imagery alone; both situations likely occur. Financial setbacks or conflicts with communities may prevent planned development from going forward in some cases (27, 28). Plantation failure may also play a role in idle land creation; some areas classified as idle in our study may have been planted with oil palms only to have the seedlings fail before they could be detected in with satellite imagery, due to fire or improper drainage in peatlands. In other cases, such as in the failed Mega-Rice project in Central Kalimantan, inadequate planning may have resulted in forests being cleared (both mechanically and by stand-clearing fires in drained peatlands) then abandoned, as the subsequent steps needed to prepare the land for cultivation were never carried out or not considered in project planning. Forest clearing may also be a way for companies to secure control over land within their concessions while simultaneously benefiting from timber sales (17). Companies holding selective logging or timber plantation concessions, which cannot legally be planted with oil palms, sometimes neglect to replant cleared forests, as their license agreements require, in order to compel the government to rezone land for nonforestry uses (28). Conversely, oil palm license holders operating in remote areas sometimes clear land well before palm oil processing mills have been established. In these cases, timber extraction may be the primary motivation for securing the license (27).

Land speculation also occurs at smaller scales. In Kalimantan, forest land cleared by local actors was commonly sold to nonlocal buyers, particularly along roadsides, near urban areas, and in areas targeted for future palm oil development (16). The buyers in these cases do not intend to develop the land, but instead plan to resell as land prices rise. In Sumatra, ready-to-plant land that had been cleared and then burned was purchased at a higher price from buyers looking to establish oil palm plantations compared to the price of uncleared land or land that had been cleared but not fertilized through burning (29, 30). In areas where government-issued land certificates are difficult or impossible to obtain; for example, due to prohibitive costs, existing land conflicts, or zoning restrictions; clearing or otherwise “transforming” land is also a way to assert customary ownership or to obtain ownership letters from village governments, which are issued more freely but carry less legal weight (31). This can enable community members to benefit from land sales to concession holders or local elites (31, 32). This may be a particularly important dynamic in situations where communities feel they are at risk of losing access to customary forests, such as when a plantation concession overlaps with community forest land. In these cases, local actors may opt to clear and sell land rather than embark on what they may view as a losing battle to maintain control of their customary forests. Transforming and then selling land may also occur in areas where plantation development is illegal, for example within state forest land, as politically powerful land buyers are better positioned to operate illegal plantations with impunity compared to less powerful community members (31). We do not know the extent to which forest clearing patterns in these cases differ from other smallholder land use activities. In some cases, these transformations may not be distinguishable from smallholder land use in the satellite imagery. For this reason, this study may underestimate the role that land speculation plays in driving forest loss in Indonesia, particularly outside of industrial areas.

Since most forests are degraded before they are cleared, demand for high value timber is unlikely to be the primary factor driving idle land creation. Demand for low-value pulpwood, however, may play a role. This is most likely to have been the case in the 1990s, when pulp production outstripped the development of new pulpwood plantations and companies routinely violated requirements to replant cleared natural forests (28, 33). More recent research showing that deforestation increases, rather than decreases, land value suggests that timber is not currently the principal idle-land driver (29, 30).

While oil palm expansion was common in mechanically cleared unplanted areas and, to a lesser extent, in areas deforested by fire, oil palms rarely replaced recently cleared (after 1990) shifting cultivation or permanent smallholder agriculture. This is a somewhat surprising finding, given that community conflicts with palm oil plantation companies are widespread. It is possible that these conflicts typically occur in smallholder areas that were cleared before 1990. We did not track land use trajectories in those areas, as they did not meet our definition of primary forest in 1990 and were outside the scope of this study. Industrial oil palm expansion may also be more common in customary forests compared to community agricultural areas (34). Since our smallholder land use class only included deforested areas, plantation establishment in customary forests would not be distinguishable from other primary forest conversions in our study.

Our study confirms the recent drop in deforestation observed in estimates derived from map pixel counting (1, 35) and reported by the Indonesian government (36). Further, we found that most deforestation occurred in relatively flat areas well suited for oil palms. In Sumatra, where roughly 40% of the deforestation observed in the study occurred, only 3.3 (±0.2) Mha of low-slope/low-elevation primary forest remained. The recent deforestation decline, particularly in regions with historically high deforestation rates, may be partly attributable to the fact that few forests remain in areas conducive for plantation development.

The findings of this study have important policy implications. First, our study confirms that oil palm expansion was a major contributor to forest loss in Indonesia from 1991 to 2020. Moreover, the real extent to which palm oil drives deforestation is likely much greater than the area directly converted, as it was by far the most common economically productive outcome for land that was initially left idle. Companies that produce and use palm oil have increasingly adopted “No Deforestation, No Peat, No Exploitation” (NDPE) commitments, with most of the industry covered by such commitments (37). The European Union (EU) has also adopted increasingly strict policies regarding deforestation-linked commodities, including a ban on certain imports unless companies can demonstrate that their supply chains do not contribute to forest loss (38). Delayed oil palm planting in deforested areas makes it more difficult to assess the commodity’s true impact on primary forests or to accurately determine whether supply chains are deforestation free. However, the EU’s import restrictions and some NDPE policies apply only to commodities planted on land deforested after 2020. Our study found that Indonesia contains an extensive area of unproductive land that could potentially be used for commodity production without the need to clear any additional forests. We estimate that 30.9% (±1.9) of Indonesia’s cleared primary forest area was unproductive in 2020, including 4 Mha (±0.27) cleared mechanically. Most of this idle land (73.1% ± 2.1) is located in areas where the terrain (slope and elevation) is well suited for oil palms (25). While trees may have regrown in some idle areas, repeated clearings in idle land are common; over half (52.2 ± 2.3%) of the 2020 idle areas experienced repeated unproductive clearings, meaning these areas likely remain low carbon landscapes.

As part of its Nationally Determined Contribution under the Paris Agreement, Indonesia has pledged to turn agriculture, forestry, and other land uses (AFOLU) into a net carbon sink by 2050, or by 2030 with foreign assistance (18). Reducing deforestation and increasing carbon sequestration through reforestation are both important parts of these plans. Our study confirms that a large area of underutilized land exists that could potentially be reforested. However, our research also found that idle areas are often converted to productive uses. Government plans to reforest these areas may conflict with existing plans by land users to develop plantations. At the same time, although Indonesia has pledged to reduce deforestation and is experiencing historically low forest loss rates, its emissions reduction strategy still allows for between 14.6 Mha and 6.8 Mha of government-sanctioned deforestation by 2050 (18). Our study demonstrates that there is already an extensive deforested area that has not yet been converted to a productive use, and the land use trajectories we documented suggest that plans may already exist to establish oil palm plantations in some of these landscapes. If Indonesia restricted commodity production to areas deforested before 2020, not only would there be extensive land available for palm oil and other export crops, but it would help ensure that commodities produced in Indonesia complied with EU requirements and private-sector NDPE policies.

## Materials and Methods

We used a probability sample to estimate the area of intact and degraded primary forest in Indonesia in 1990 and the annual rates of intact primary forest degradation, primary forest loss, and postdeforestation land cover change, disaggregated by land use, from 1991 to 2020. This method is consistent with good practice recommendations for estimating land cover areas, as a probability sample allows us to estimate uncertainties (we report uncertainty as ±1 SE of an estimate) and avoids the biases inherent in area estimates derived from map pixel counting (39). Similar methods have been employed in prior studies estimating forest loss areas and attributing forest disturbance drivers in the Brazilian Amazon and the Congo Basin (40, 41). For this study, we selected a simple random sample from each of the seven regional strata—Sumatra, Kalimantan, Java, Sulawesi, Nusa Tenggara, Maluku, and Papua. A total of 10,000 sample units were selected, with the sample size per region proportional to the region’s area. The sample units were 0.00025° pixels, which is roughly 30 m × 30 m for this equatorial region. The areas, sample sizes, and sampled pixel locations for each stratum are provided here: https://glad.umd.edu/users/Parker/Indonesia_Reference/index.html.

### Land Cover/Land Use and Change Event Definitions.

We define primary forest as natural, old-growth woody vegetation at least 5 m in height and with at least 30% tree cover at a 30-m spatial resolution. This is divided into intact primary forest, with no observable evidence at a 30-m resolution of human disturbance within a 140-m radius, and degraded primary forest, which shows evidence of human disturbance within a 140-m radius but maintains at least half of its original natural canopy at a 30-m resolution. A 140-m radius from the center of a 30-m sample unit was used to differentiate intact from degraded primary forest as it roughly corresponds to the 6.25-ha minimum mapping unit used by the Indonesian Ministry of Environment and Forestry to define primary forest (36).

Sampled pixels not classified as either intact or degraded primary forest in 1990 were assigned to the nonprimary forest category. This comprised pixels without tree cover as well as those with tree cover but containing less than 30% natural tree canopy cover, or pixels for which greater than 50% of the natural vegetation showed evidence of having been cleared in recent history. This included: a) shifting cultivation fallows or tree crops (e.g monoculture rubber, oil palm, or timber plantations) planted before 1990; b) mixed agroforestry areas containing greater than 50% cultivated tree species, to the extent that these areas could be distinguished from natural forest; and c) secondary forests, such as mangrove forests replanted after clearing for fishponds or fire-cleared areas that had been allowed to regrow or were otherwise rehabilitated.

We tracked three types of change events: primary forest degradation, primary forest clearing, and postdeforestation land cover change. We define forest degradation as any disturbance that resulted in less than 50% natural canopy loss within a 30-m resolution pixel or a disturbance within a 140-m radius of the pixel’s center. Examples of forest degradation events include selective logging, non-stand-clearing fires, and nearby clearing within 140 m from the sampled pixel (*SI Appendix*, Fig. S3). We also recorded degradation from peatland draining, including, in some cases, from canals built more than 140 m from the sampled pixel. Canal construction occurring >140 m from the sampled pixel was only classified as degradation if the pixel also experienced a change in its spectral characteristics at a 30-m resolution, indicating a partial canopy disturbance. All degradation events observable in the reference data were recorded in forests that were both intact and degraded in 1990. The first degradation event observed in forest that was still intact in 1990 was used to estimate annual intact forest degradation.

We define primary forest clearing as the loss of at least 50% of the natural canopy at a 30-m spatial resolution, regardless of postclearing land use. Primary forest clearing was identified based on changes in the spectral characteristics of the sample unit, including a temporary or permanent increase in bare ground or a persistent change in the vegetation signature indicating a substantial canopy disturbance. Loss events and the percent canopy loss (≥50% for deforestation, <50% for degradation) were confirmed with very high-resolution data (1.5 m or <1 m) where available. Postdeforestation land cover change was defined as a land cover change impacting at least 50% of the sampled pixel, occurring at least 12 mo after the initial primary forest clearing event.

Change events, including primary forest clearing and postdeforestation land cover changes, were further categorized by land use. The land use categories we identified in this study included: oil palm plantations, smallholder land use, tree plantations, built-up land, and other land uses. Land use class examples can be found in *SI Appendix*, Fig. S6.

The oil palm plantation class included both large-scale plantations and smallholder oil palm. Plantation infrastructure or unplanted plantation blocks were excluded; at least one oil palm must have been planted within the sampled pixel for it to be included in the class. The presence of oil palms within the pixel was confirmed using high-resolution (Satellite pour l’Observation de la Terre (SPOT) 6/7 or Google Earth) imagery. The date oil palms were planted was determined using bimonthly composites and the multiyear regrowth pattern evident in the 16-d Landsat graphs (*SI Appendix*, Fig. S7).

Smallholder land use included both shifting cultivation and permanent or semipermanent small-scale agriculture, excluding rubber and monoculture oil palms. Permanent smallholder agriculture was identified by the clearing pattern, plot size, and a temporal spectral pattern indicative of crops. For wet rice cultivation, periodic field flooding was another indicator. Shifting cultivation was identified by the landscape-scale clearing patterns and regrowth during the fallow period. Shifting cultivation areas remained in the smallholder class during the fallow periods, as this stage is part of the same land use. While fallows may be spectrally similar to forest regrowth after a fire or mechanically cleared unplanted land, the spatial patterns are distinct, which allowed shifting cultivation land to be differentiated from both idle land classes.

Tree plantations included both slow-growing hardwoods, such as teak, and fast-growing pulpwood species, such as acacia. Fast-growing species were more common. Tree species used primarily for their fruits or resins, such as rubber or nutmeg, rather than for timber, were excluded from this class, to the extent that they could be differentiated from timber plantations by their spectral characteristics or using high-resolution data. Tree plantations were identified based on the clearing pattern (typically large, rectangular plantation blocks, similar to industrial oil palm), spectral signatures, and regrowth patterns. Unplanted plantation blocks were not included in our tree plantation category and were instead classified as either mechanically cleared unplanted land or fire clearing, depending on the clearing mechanism. Fast-growing pulpwood plantations could usually be identified by their spatial and temporal patterns alone; high-resolution imagery, the multiyear regrowth pattern, and the spectral signature of the mature trees were used to identify slower-growing plantations.

Built-up land included roads, settlements, and other human infrastructure such as factories, ports, or airports. Other land uses included rubber, fishponds, mining, monoculture tree crops such as nutmeg, cloves, or coconuts, and other clearing types not assigned to the above categories. Fishponds and mining were identified using their spectral signatures and clearing patterns and were confirmed using high-resolution imagery. Fishponds have low reflectance typical of water, appearing dark in our composite imagery, while artisanal mining appears very bright. These classes also have distinct spatial patterns. Rubber, the most common driver observed in our other land use category, was identified by its spectral signature, canopy texture, and regrowth pattern. For this study, we included both large- and small-scale monoculture rubber; we did not include mixed rubber agroforestry. Rubber appears yellow-green in a Landsat SWIR-NIR-Red composite, compared to the darker green of natural forests, with reflectance in the SWIR1 band remaining higher than a natural forest, even after reaching a closed-canopy state. An example of primary forest conversion for rubber can be seen in *SI Appendix*, Fig. S7. Monoculture rubber also has a canopy texture that is more uniform than a shifting cultivation fallow or mixed agroforestry area; these textural differences can be seen in high-resolution imagery, including SPOT 6/7 composites and Google Earth imagery. Other monoculture tree crops may have similarly uniform canopies. However, since these were also included in our other land use class, we do not expect this limitation to have an impact on our findings. Coconuts have spectral signatures and tree crowns that can appear similar to oil palms, but planting blocks are much smaller in size and have a distinct spatial pattern. Google Earth photos were also used, where available, to confirm coconut landscapes. Mixed agroforestry areas, which are typically colocated with other smallholder activities, were included in our smallholder land use class.

For a change event to be associated with a land use, the land use must have been established within 12 mo of forest or land cover clearing. For tree species, such as oil palms, land use establishment was defined by the date trees were planted. This date was determined using a combination of reference data sources, primarily the regrowth pattern evident in time-series composite imagery and time-series spectral plots (*SI Appendix*, Figs. S6 and S8). High-resolution imagery was used to confirm the planting date where available.

In the absence of a postclearing land use, we classified primary forest clearing events based on the clearing mechanism: fire clearing or mechanical clearing. Deforestation by fire was defined as a fire event in a standing forest resulting in ≥50% canopy removal, identified by a spectral signature consistent with burning or ash and/or a clearing pattern consistent with fire (for instance clearing without sharp geometric boundaries) that was not planted or converted to a land use for at least 12 mo after the clearing event. Land burned after being manually cleared (slashed and burned) was not classified as fire clearing. These areas were distinguished from fire clearing by the clearing and regrowth patterns and the timing of the ash signature, if one occurred, in bimonthly or 16-d composites (*SI Appendix*, Fig. S2). Moderate Resolution Imaging Spectroradiometer (MODIS) active fire hotspot data were used to supplement bimonthly and 16-d composites (42, 43).

Mechanically cleared unplanted land was defined as land left unplanted for one or more years following manual or mechanized vegetation removal. These areas were identified by a spectral signature consistent with vegetation removal and not indicating fire clearing (e.g. no ash signature or a delayed ash signature, geometric clearing patterns, and the absence of remnant trees) and regrowth signatures consistent with grass or shrubs. Ash is distinguishable from bare ground by its color in the bimonthly and 16-d imagery; ash appears dark purple in SWIR-NIR-Red composites, compared to the bright pink color characteristic of bare ground (*SI Appendix*, Fig. S2). For pixels left unplanted at the close of the study period, the absence of productive crops was confirmed using very high-resolution SPOT 6/7 and/or Google Earth imagery. Clearing patch size and landscape patterns were also used to distinguish unplanted land from rotational agriculture, which has a similar spectral profile but can be identified by its distinct clearing pattern. Examples of fire-cleared land and mechanically cleared unplanted land can be found in *SI Appendix*, Fig. S9. An example of residue burning after mechanical clearing for unplanted land can be found in *SI Appendix*, Fig. S2.

Our ability to differentiate mechanically cleared unplanted land from deforestation by fire was more limited in areas that had large gaps between cloud-free observations around the time of clearing. This is particularly true in cases where debris was burned after clearing, as a moderate gap of several months could obscure the initial bare ground signal, potentially leading to an incorrect classification of deforestation by fire. Very long observation gaps (for example, gaps longer than 12 mo) can mean the ash signature that occurs after a stand clearing fire is no longer visible when change is detected, potentially leading to an incorrect classification of mechanically cleared land. Gaps in cloud-free observations at the time of clearing are plotted for both deforestation by fire and mechanically cleared unplanted land annually in *SI Appendix*, Fig. S10. Moderate gaps (<12 mo) were more common than longer gaps in both mechanically and fire cleared areas.

Some planted areas may have been classified as “idle” if slow-growing crops were planted but the seedlings died before they could be detected in the satellite imagery (for example, before they reached the closed canopy stage). If plantations failed before plantings could be detected, land was classified based on the initial clearing mechanism. To the extent that plantations failed due to fire, this could have led to an underestimation of fire as an idle land driver. Since early plantings can be detected in high-resolution imagery, this type of classification error would have been most likely in areas cleared before 2013, the first year for which we had SPOT 6/7 reference data, and in areas/eras with fewer Google Earth images.

Frequent postdeforestation clearing events were common in idle land areas. For postdeforestation land cover change events, it was more difficult to determine the clearing mechanism, since the ash signature in a burned grassland is more ephemeral. In cases where repeated idle land clearing events took place, the events were recorded, but the classification remained either fire clearing or mechanically cleared unplanted land, depending on the mechanism associated with the first idle clearing event, until an economically productive land use (oil palm plantations, smallholder land use, tree plantations, other land uses, or built-up land) was established.

The land use was unclear for 1.8 (±0.2) Mha. In some of these areas (46.3% ± 5.1) a previous land use had been identified, but the land use in 2020 could not be confirmed due to a subsequent land cover change event. The remaining 53.7% (±5.1) was either converted directly from primary forest or represented a land cover change on idle land. Most of the areas where land use could not be determined experienced a clearing event in either 2019 or 2020, a period with less available high-resolution imagery.

### Reference Data Sources.

Analysts interpreted satellite-derived reference data for each sampled pixel, recording the 1990 land cover class and, for primary forest pixels, any subsequent change events, including the initial forest degradation and/or deforestation event, and all subsequent land cover and land use transitions. Reference data used for visual interpretation included annual, bimonthly (spanning 2 mo), and 16-d Landsat composites; graphs of annual minimum and maximum normalized difference vegetation index (NDVI) values and 16-d NDVI, SWIR1, and SWIR2 values derived from quality-screened Landsat data (44); graphs of MODIS active fire detections within a 5-km radius (42, 43); SPOT 6/7 composites for the 2013 to 2020 period; and Google Earth imagery.

Landsat composites and graphs were created using Landsat Analysis Ready Data (ARD) (44). Annual composites included mean SWIR1-NIR-Red composites for the years 1985 to 2020. These were created from only clear land observations from the relevant year. If no clear observations existed, pixels were displayed in black, indicating no data. Annual composites using SWIR1-NIR-Red values from the lowest normalized burn ratio (NBR) date were also included as reference data for 1989 to 2020. The mean annual composites covered a 41 × 41 pixel area, with the sampled pixel in the center. NBR composites covered a larger 5 km by 5 km area to better detect the shape of large nongeometric clearings typical of stand-clearing fires.

Bimonthly images included both mean SWIR1-NIR-Red and mean NIR-SWIR1-SWIR2 composites derived from ARD data for the 1989 to 2020 period. For bimonthly images, pixels without any clear observations were gap-filled with cloud-contaminated data from the same period. For the years for which MODIS active fire data were available (2000 to 2020), annual graphs showing the dates and percent confidence for any active fire hotspots occurring either ≤1 km or ≤5 km from the sampled pixel were also included as reference data (42, 43). Indonesia’s National Institute of Aeronautics and Space (LAPAN) provided five pan-sharpened 1.5 m resolution SPOT 6/7 composites for each sampled pixel for the following time periods: 2013 to 2016, 2017, 2018, 2019, and 2020. Very high-resolution data in Google Earth were also evaluated where available.

Sixteen-day Landsat composites were employed for 1989-April 2021; these data were used to confirm the date and type of disturbance in cases where the bimonthly composites were insufficient and to detect clearings that may have occurred in late 2020, which could potentially be missed in the bimonthly data. Reference data pages and sample interpretation results are available at: https://glad.umd.edu/users/Parker/Indonesia_Reference/index.html.

### Interpretation Process.

Sampled pixels were reviewed by a team of seven analysts from Indonesia’s National Research and Innovation Agency (BRIN, formerly LAPAN), Indonesia’s Ministry of Environment and Forestry (KLHK), the World Resources Institute (WRI) of Indonesia, and the University of Maryland (UMD), using an iterative approach. First, a subset of the reference data (annual SWIR1-NIR-Red composites from 1985 to 2016, annual NDVI min/max graphs, and the 2013-2016 SPOT 6/7 image) was interpreted by the team, which consisted of two analysts each from KLHK, BRIN, and WRI-Indonesia and one from the University of Maryland. Analysts assigned each sampled pixel to a 1990 land cover class—intact primary forest, degraded primary forest, or nonprimary forest—and recorded their level of confidence (high or low) in their interpretation. For sampled pixels classified as either intact or degraded primary forest in 1990, all disturbance events (degradation, forest clearing, and postdeforestation land cover change) occurring between 1991 and 2016 were also recorded. Analysts recorded the year the disturbance event took place and their confidence in their interpretation. They also assigned land change drivers to all forest clearing and postdeforestation land cover change events.

This process was carried out through a series of workshops. The sample interpretation legend was developed collaboratively, and participation of the national experts was crucial in developing the legend. During the interpretation process, analysts worked together in the same room and were encouraged to ask questions of other team members when confronted with difficult pixels. For each regional stratum, a small subset of sampled pixels was discussed by the entire team and consensus reached as a group. This was done to ensure that the classification legend was applied consistently by the entire team.

Interpretation results were then compared to several existing forest extent and change products produced by the participating institutions: KLHK forest/nonforest maps (1990, 2000, 2012); Margono et al. and Turubanova et al. primary forest maps (2000 and 2001) (1, 45); tree cover maps created by LAPAN as part of the Indonesian National Carbon Accounting System (INCAS) project (2000 and 2012) (46); and Hansen et al. percent tree canopy cover (2000) and tree cover loss (2001 to 2016) products (35). In cases where results were inconsistent with existing products or cases with low-confidence interpretations, sampled pixels underwent additional review. Inconsistencies were defined as follows:

• Pixels classified as forest (an aggregation of the hutan primer and hutan sekunder land cover classes) in one or more KLHK product, but classified as nonprimary forest in our study in the corresponding year; or pixels classified as nonforest by KLHK but classified as primary forest in our study;
• Pixels classified as primary forest by Margono et al. (45) in 2000 or Turubanova et al. (1) in 2001, but classified as nonforest in the corresponding year in our study; or pixels classified as nonprimary forest in the above-mentioned products but classified as primary forest in our study;
• Pixels classified as nonforest in the INCAS 2000 or 2012 products, but classified as primary forest in the corresponding year in our study (the INCAS forest definition included nonprimary forest tree-covered landscapes, so pixels classified as forest by INCAS but nonprimary forest in our study were not deemed inconsistent) (46);
• Pixels identified as having <80% tree canopy cover in the Hansen et al. global tree canopy cover product in 2000 (35), but classified as primary forest in our study in that year;
• Pixels for which the year of first clearing in our study occurred after 2000, if the year of clearing was more than three years before or after tree cover loss was identified in the Hansen et al. tree cover loss product or if no loss event was identified in the Hansen et al. product (35);
• Primary forest pixels for which no loss events were identified in our study, but which were identified as loss in the Hansen et al. global tree cover loss product (35).

Results were first compared to two products: the 1990 KLHK forest/nonforest map and the Turubanova et al. primary forest map (1). Inconsistent pixels were then reevaluated by the original assigned analyst, who revised their interpretation as they deemed appropriate. The resulting dataset was then compared to all the above-listed products. Sampled pixels with interpretations inconsistent with the above products, as well as all pixels experiencing forest loss or classified as “low confidence” by the original interpreter were then reviewed by the UMD analyst, who assigned them to one of three categories: agree, unsure, or recommend change. Sampled pixels classified as “unsure” or “recommend change” were then reviewed by a group of two or more Indonesian team members. Revisions were made based on the consensus reached by these analysts; groups were not required to revise pixels assigned to either “unsure” or “recommend change” categories.

Additional reference data were later added for the 2017-2020 period. Bimonthly, 16-d, and annual NBR composites and 16-d NIR, SWIR1, and SWIR2 graphs were also added to allow clearing dates and mechanisms to be determined with more precision. We reviewed the updated annual SWIR1-NIR-Red composites, 16-d Landsat graphs, and SPOT 6/7 composites for all 10,000 sampled pixels at the University of Maryland; bimonthly composites, NBR composites, and MODIS active fire data were also reviewed for all sampled pixels classified as either intact or degraded primary forest to assess possible additional degradation events. Sixteen-day composites were reviewed in cases where a disturbance event occurred but the date or change driver could not be confirmed using bimonthly data alone. Sixteen-day composites were also reviewed for all sampled pixels left idle for 12 mo or more after the first clearing event, in order to better differentiate mechanical clearing from forest loss due to fire.

Out of 10,000 sampled pixels, 79% were initially reviewed by an analyst from KLKH, BRIN, or WRI-Indonesia, then by the UMD analyst after the additional reference data was added. The other 21% were reviewed at least twice by the UMD analyst. The 1990 land cover, 2016 land cover, first clearing year or direct driver, or the preclearing forest type, were updated by the UMD analyst after the additional reference data was added for 21% of the 10,000 sampled pixels. Of the sampled pixels reviewed by both an analyst from one of the Indonesian organizations and the UMD analyst, 20% were updated based on the added reference data, compared to 24% of pixels reviewed twice by the UMD analyst. These similar rates of change suggest that updates were made primarily based on the availability of additional reference data, rather than differences between interpreters in the application of the classification legend.

Confidence levels were recorded for each change event. The proportions of high and low confidence interpretations for the land use immediately after clearing and the final land use are displayed for all sampled-pixels experiencing forest loss in *SI Appendix*, Fig. S11.

### Comparison to Prior Studies.

We detected a larger area of primary forest loss in Indonesia compared to several previous studies (1, 12, 13). Gaveau et al. found 9.79 Mha of primary forest loss from 2001 to 2019, while we estimated 14.6 (±0.5) Mha of loss during the same period (12). We also estimated a larger area of primary forest converted to oil palm from 2001-2019, 4.1 (±0.3) Mha compared to 3.09 Mha. Our 2019 primary forest area estimates were very similar (87.4 ± 0.8 Mha compared to 87.76 Mha).

For the 2001 to 2016 period, our estimated areas of forest converted to oil palm, timber plantations, and smallholder agriculture were also larger than those found by Austin et al. (13). We estimated 2.9 (±0.2) Mha, 1.3 (±0.2) Mha, and 2.5 (±0.2) Mha of forest converted to those three land uses, respectively, within 5 y of clearing from 2001 to 2016, compared to 2.1 Mha, 1.3 Mha, 2 Mha reported for similar classes by Austin et al.

Both studies derived their total deforested estimates from the Hansen et al. global tree cover loss map overlaid with an Indonesian primary forest map (35, 45), although Gaveau et al. (12) modified the map to improve consistency. Gaveau et al. derived their oil palm estimates from expert-delineated maps of industrial oil palm plantations created using Landsat imagery and small-scale oil palm maps created using high-resolution imagery and radar data (12). Austin et al. estimated deforestation driver areas by interpreting a sample of mapped loss, using high-resolution imagery and Landsat data from the 4-y period after the deforestation event to classify drivers (13). Some of the differences in driver areas may be due to the definitions and methods employed in the various studies. The differences in the total loss areas may be due to biases in the map pixel counts. Turubanova et al. found that while map- and sample-derived primary forest areas were similar for their pan-tropical primary forest map, map-derived estimates of loss within primary forests were biased, underestimating loss compared to sample-based estimates (1). We also found higher levels of deforestation in Indonesia compared to Turubanova et al.’s map-based estimates, particularly in the early 2000s (1).

### Area and SE Calculations.

We used the R survey package (47) to estimate land cover class areas. For each class of interest, we used the *svymean* tool to estimate the population proportion of each class and the corresponding SE. We converted these proportions to area estimates by multiplying the estimated proportions and SEs by the total study area. To estimate subpopulation proportions, such as the proportional contributions of specific drivers to overall primary forest loss, we used the survey package’s *svyby* tool to produce domain estimators.

## Supplementary Material

Appendix 01 (PDF)

## Data Availability

All study data are included in the article and/or *SI Appendix*.
