# Supplementary material for: Land in limbo: Nearly one third of Indonesia’s cleared old-growth forests left idle
Source: Proc Natl Acad Sci U S A. 2024 Jul 1;121(28):e2318029121. doi: 10.1073/pnas.2318029121 (PMC11252779; doi:10.1073/pnas.2318029121)
Supplement: Supplementary file 1 — Appendix 01 (PDF) [file pnas.2318029121.sapp.pdf]

PNAS

Supporting Information for

Land in limbo: nearly one third of Indonesia's cleared old-growth forests left idle

Diana Parker*, Anna Tosiani, Muhammad Yazid, Inggit L. Sari, Tatik Kartika, Kustiyo, Rizky Firmansyah, Zuraidah Said, Arief Wijaya, Peter Potapov, Alexandra Tyukavina, Stephen V. Stehman, Viviana Zalles, Amy Pickens, Jeffrey Pickering, Svetlana Turubanova, Matthew C. Hansen.

*Diana Parker
Email: parkerd@umd.edu

This PDF file includes:

Figures S1 to S11
Tables S1 to S26

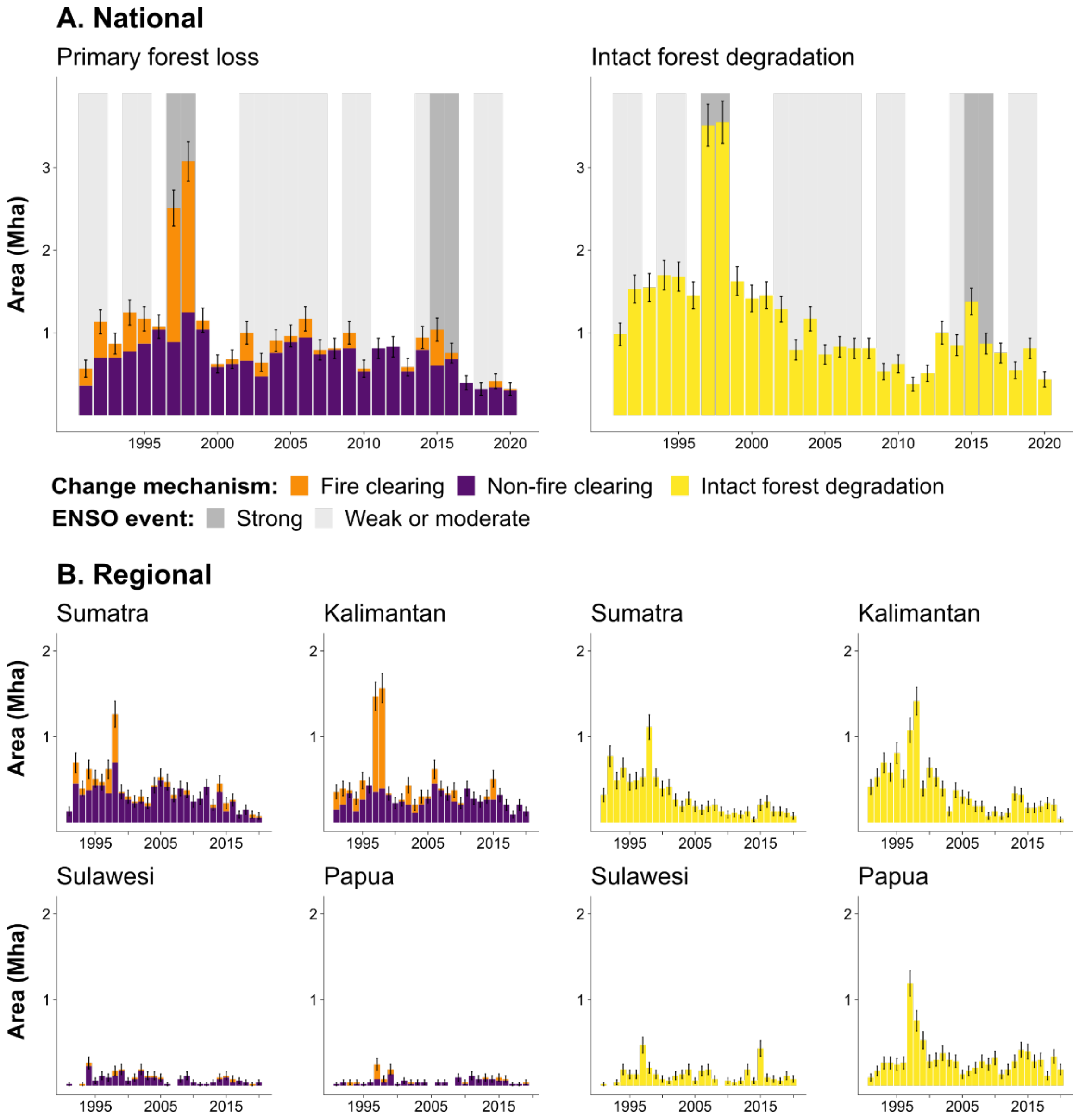

Fig. S1. Annual primary forest loss and intact primary forest degradation, nationally (A) and regionally (B). Strong and weak or moderate El Niño-Southern Oscillation (ENSO) events are highlighted in the national plots. Forest loss is disaggregated by fire and non-fire clearing.

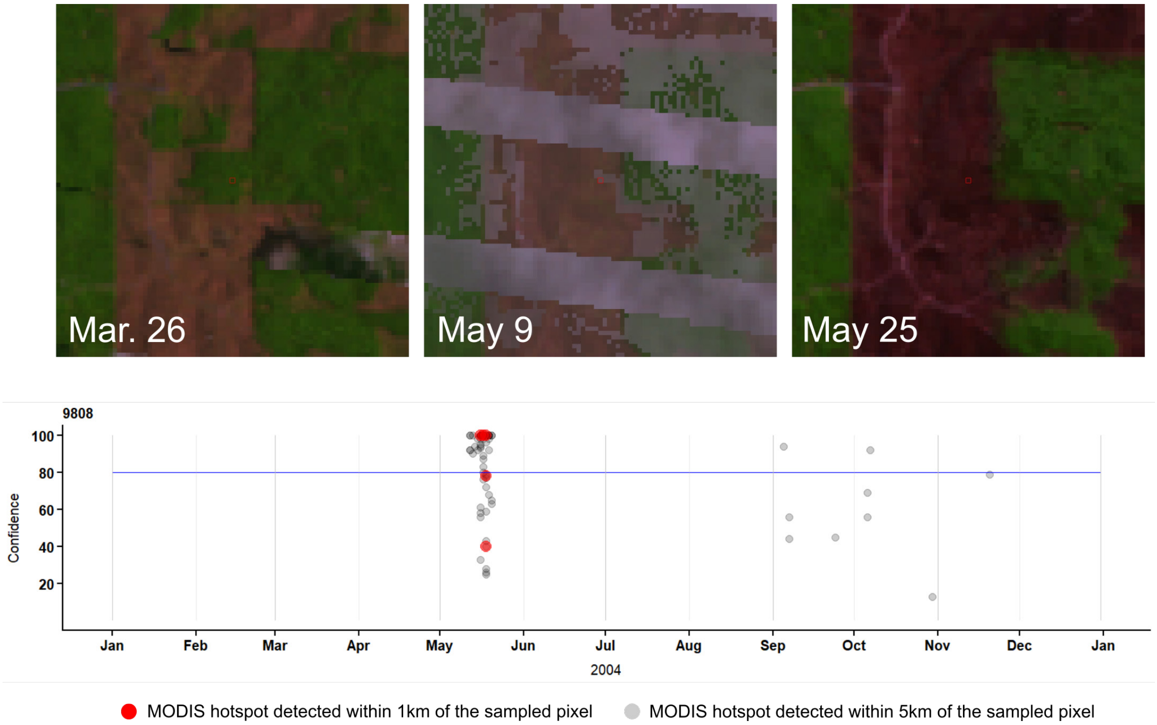

Fig. S2. Example of mechanical land clearing followed by debris burning. This sampled pixel was cleared between March 26th and May 9th, 2004. In the SWIR1-NIR-Red false color composites displayed here, the cleared land appears light pink after the initial clearing event, a spectral signature consistent with unburned bare soil. By May 25th, the spectral signature of the cleared area has become dark purple, consistent with ash. A cluster of MODIS hotspots were detected within 1-km and 5-km of the sampled pixel between May 12th and May 20th. This sampled pixel was classified as mechanically cleared unplanted land in 2004 and was later planted with oil palms in 2009.

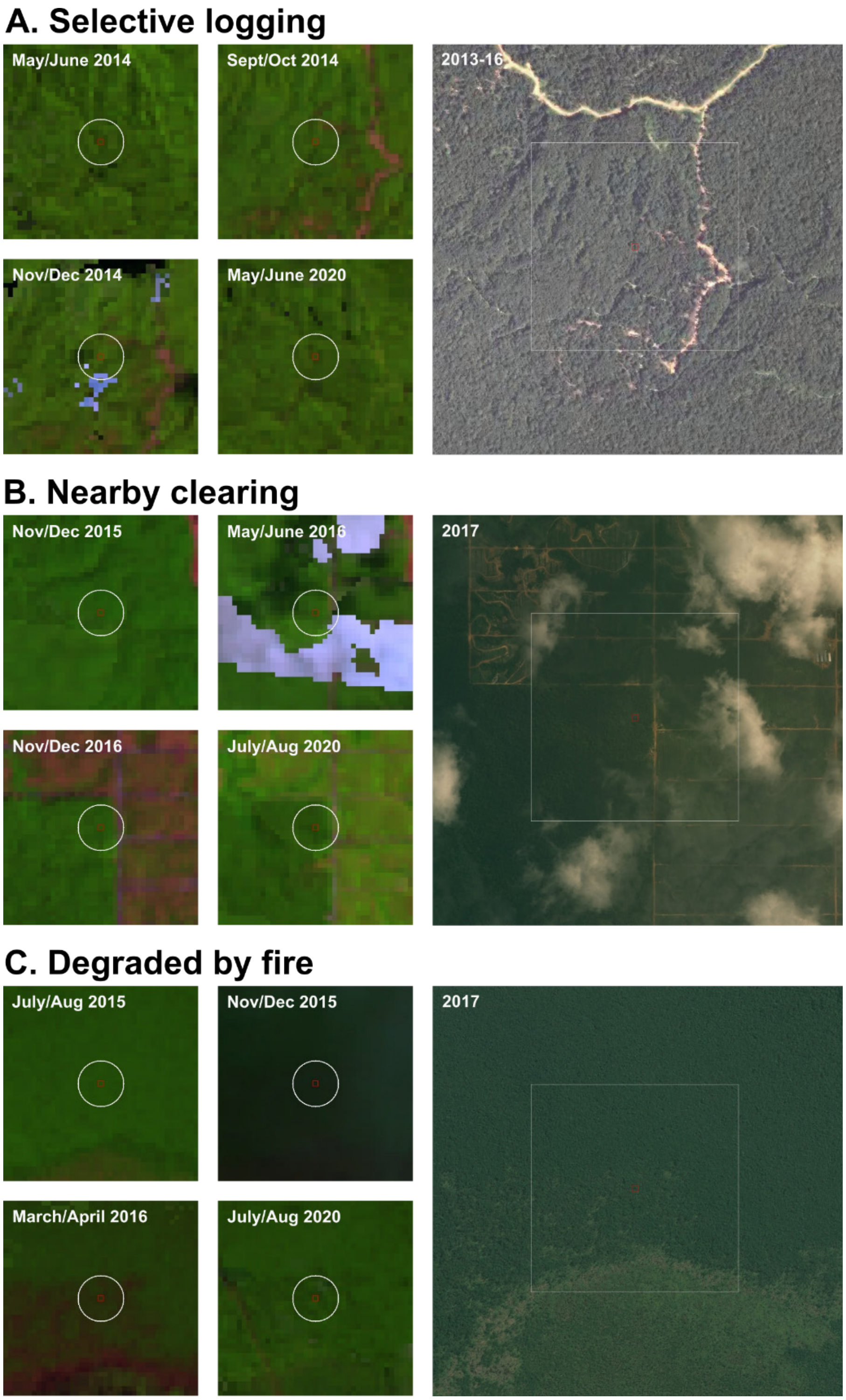

Fig. S3. Intact primary forest degradation examples. A subset of the reference data, including bimonthly Landsat composites and SPOT 6/7 composites, illustrating examples of intact forest degradation, including selective logging (A), nearby clearing (B), and degradation by non-stand clearing fires (C). We define forest degradation as any disturbance that resulted in less than 50% natural canopy loss within a 30-m resolution pixel or a disturbance within a 140-m radius. A 140-m radius around the sampled pixel is outlined in white in the bimonthly Landsat composites.

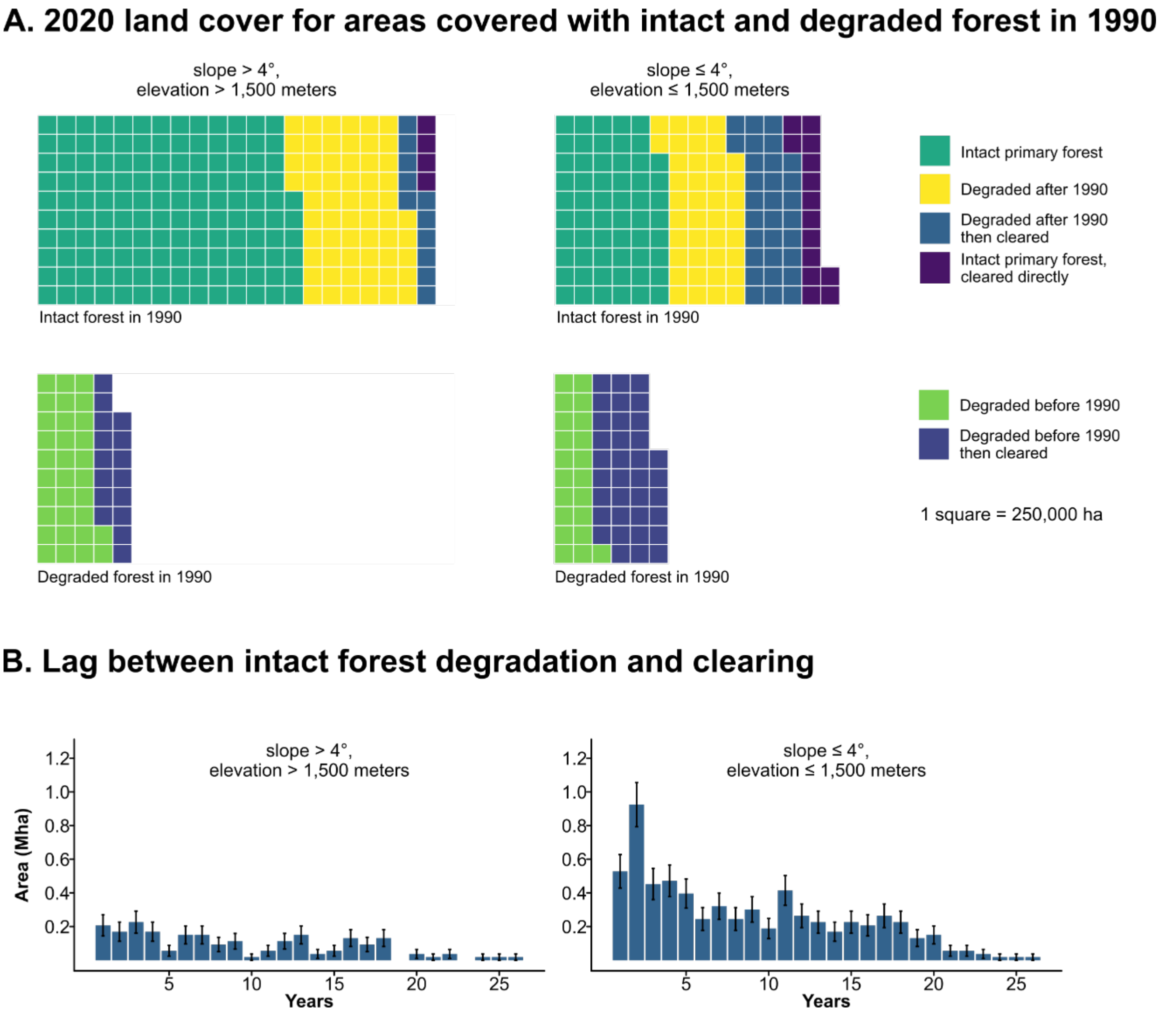

Fig. S4. Intact and degraded primary forest trajectories in areas with high slopes and elevations (>4°/1,500m) and low slope/elevation areas. Includes the 2020 status of forests either intact or degraded in 1990 (A) and the lag times between intact forest degradation and forest clearing in high and low slope/elevation areas (B).

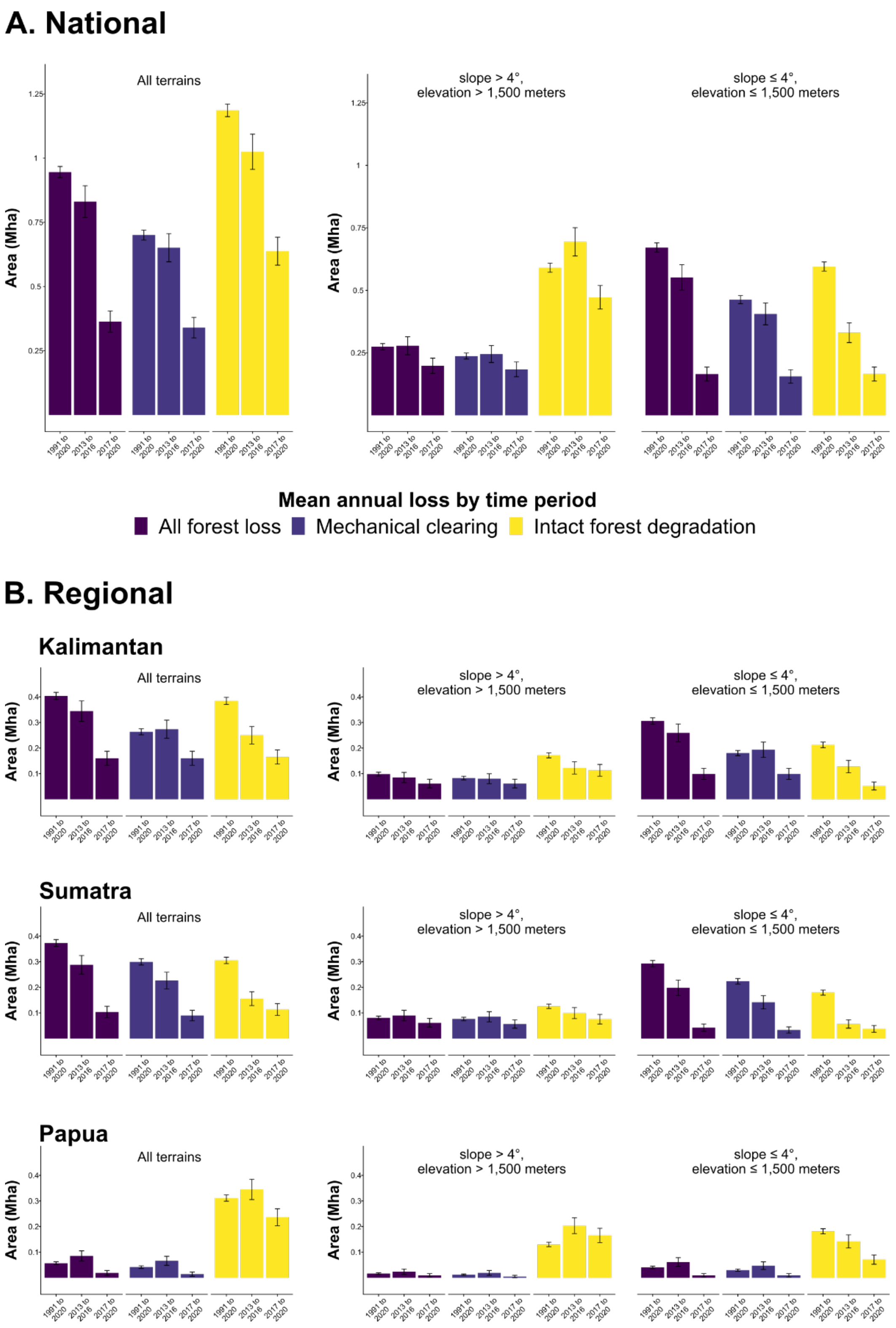

Fig. S5. Mean annual primary forest loss, annual mechanical (non-fire) clearing, and annual intact forest degradation nationally (A) and regionally (B). Mean annual loss is reported for the entire study period (1991-2020), the period from 2013-2016, and the last four years of the study period (2017-2020).

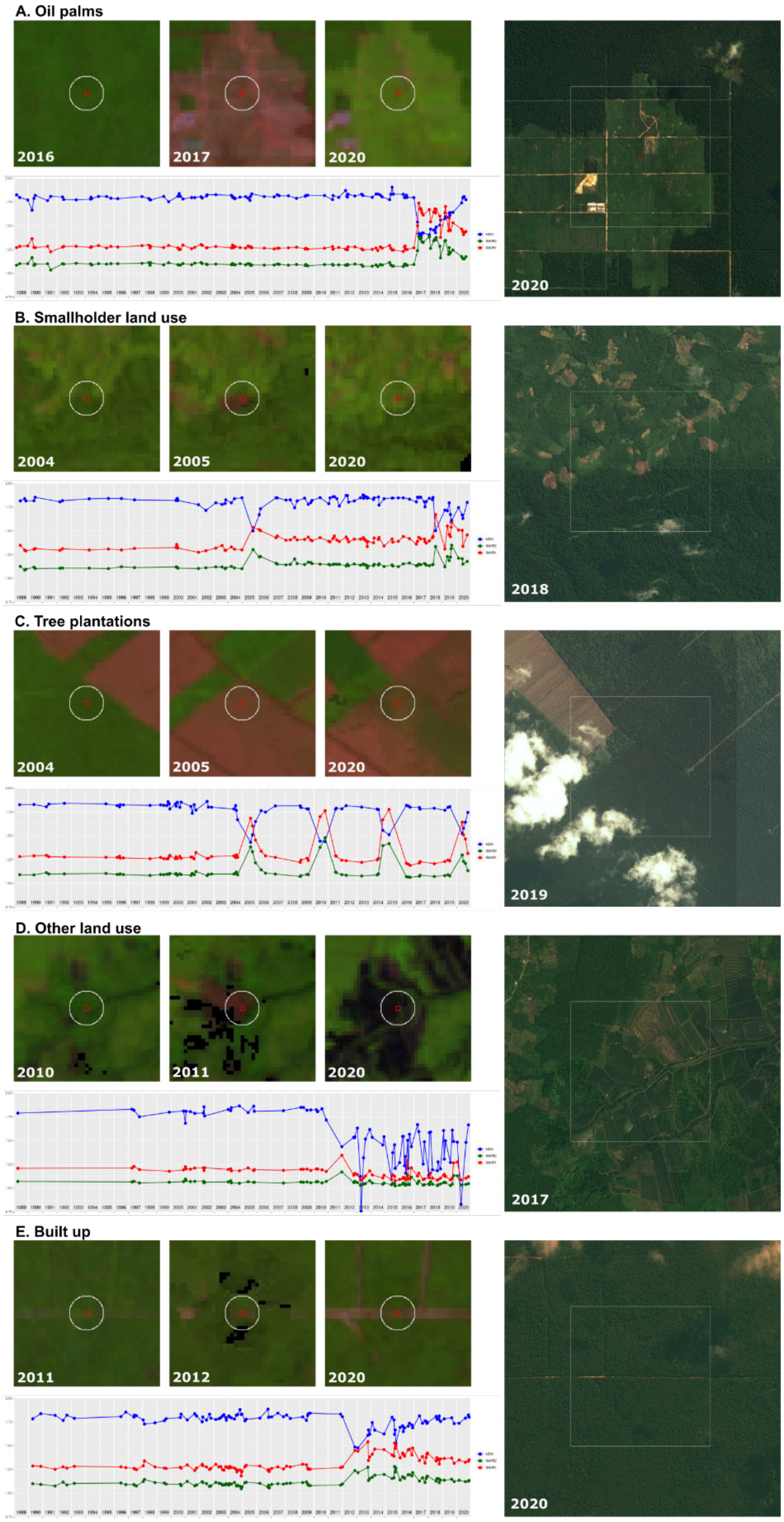

Fig. S6. Productive land use examples. A subset of the reference data, including annual Landsat composites, 16-day NDVI, SWIR1, and SWIR2 plots, and high resolution SPOT 6/7 imagery are provided here for one sampled pixel per class.

A. Annual Landsat composites

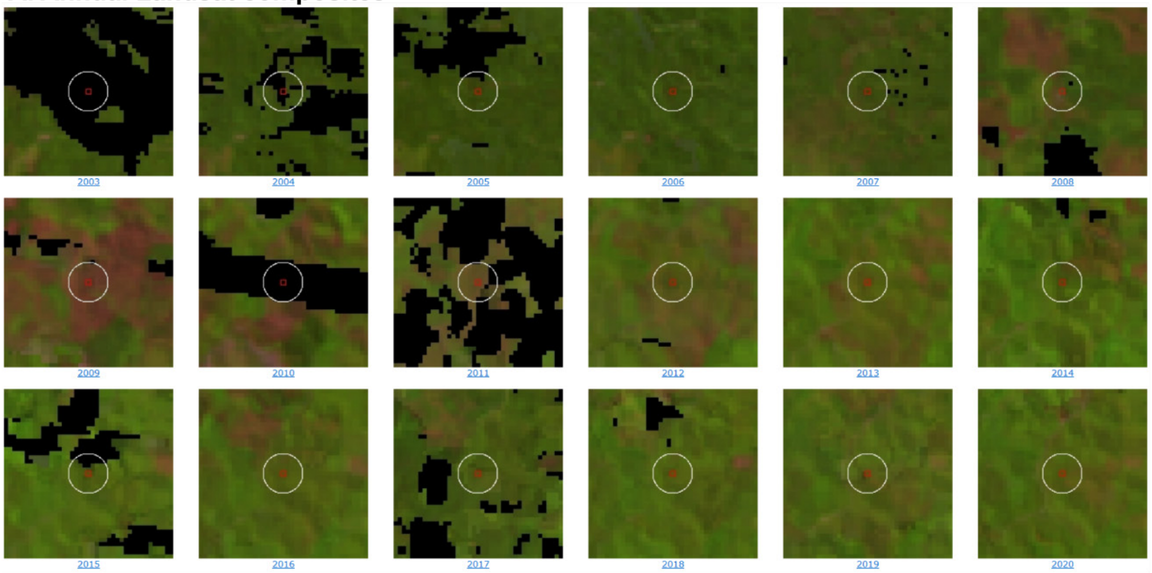

B. 16-day Landsat NDVI, SWIR1, and SWIR2 values

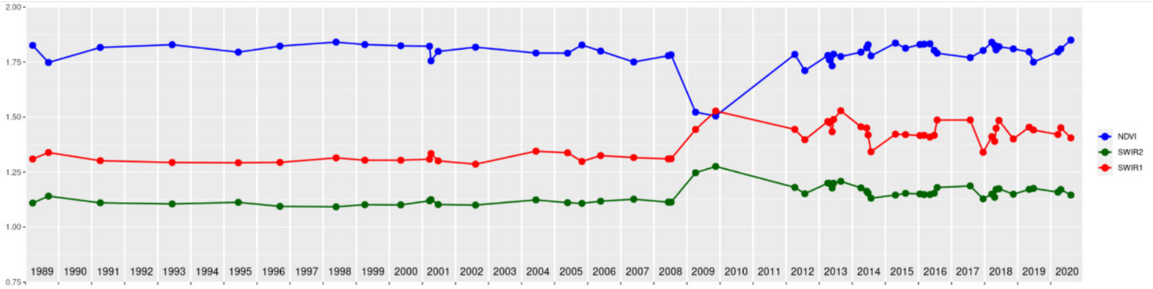

C. SPOT composites

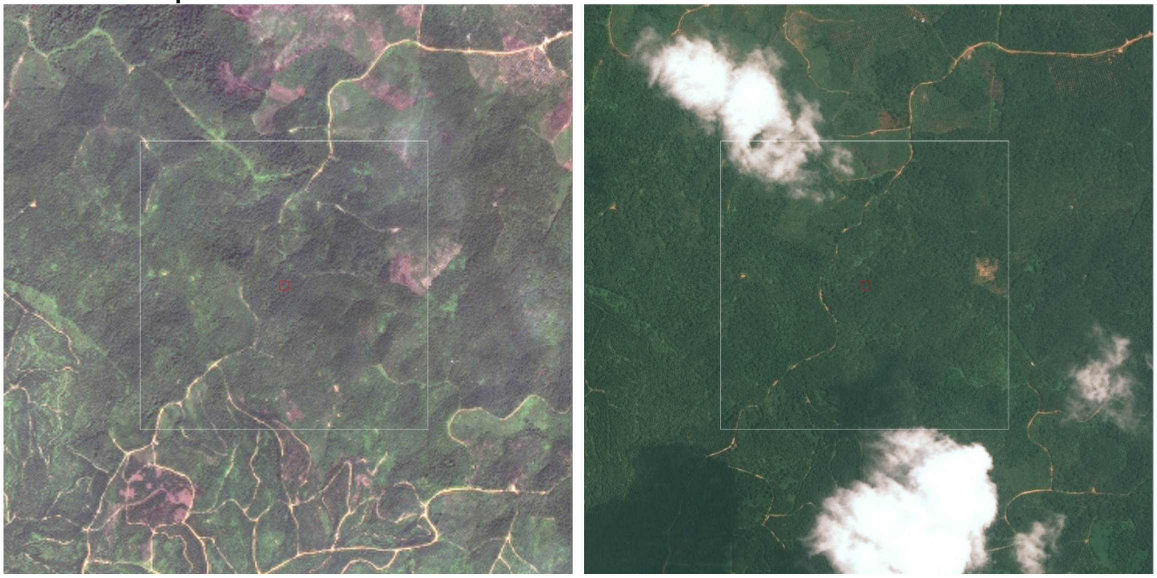

Fig. S7. Reference data for a sampled pixel experiencing forest conversion for rubber. The forest was cleared and the area planted with rubber in 2008. In the 16-day plots, the signature returns to that typical of a closed canopy, but with higher SWIR1 reflectance. In the SPOT 6/7 imagery, the canopy appears more uniform than natural regrowth, indicative of a monoculture plantation.

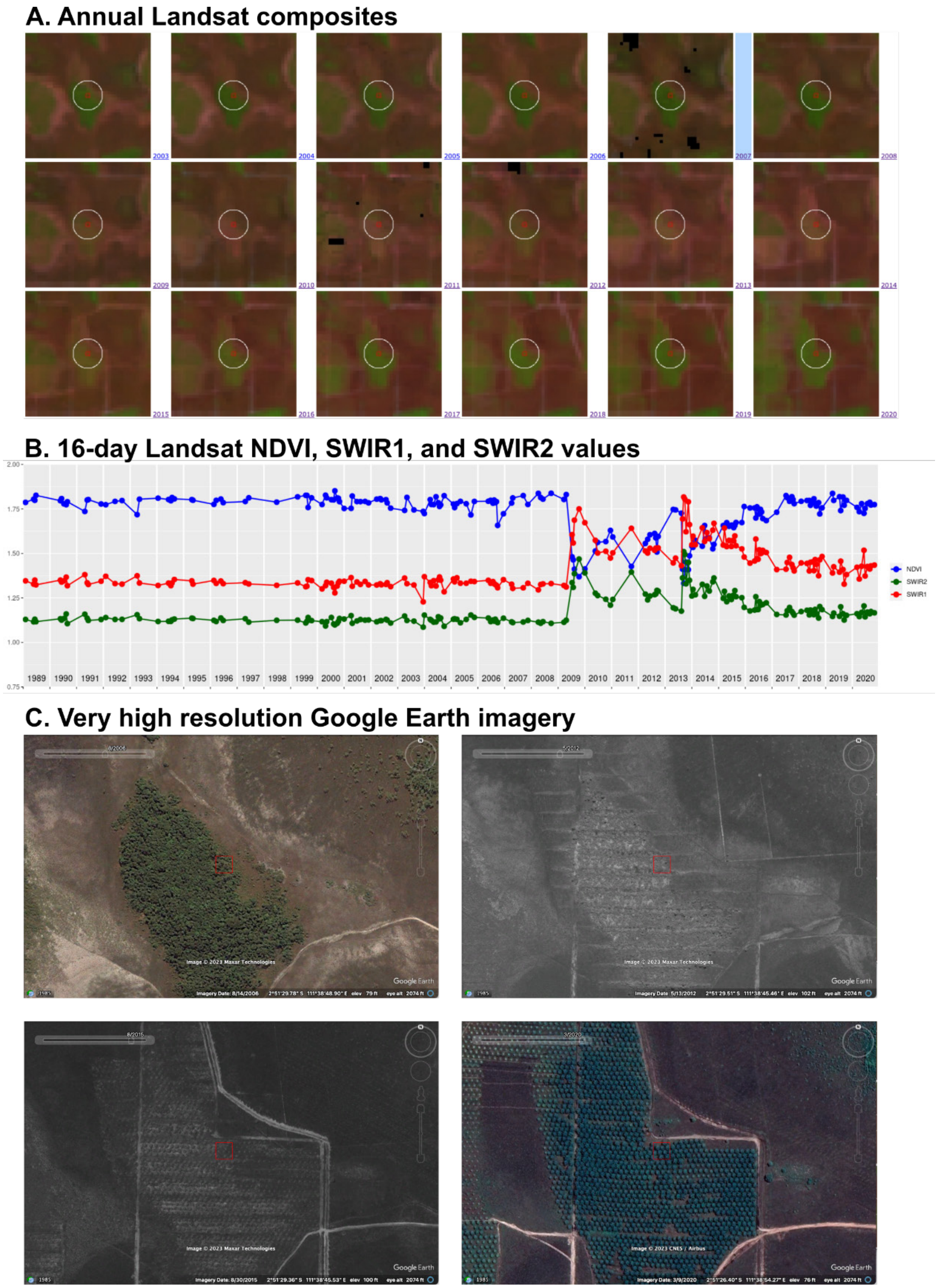

Fig. S8. Reference data for a sampled pixel that experienced delayed oil palm planting. The primary forest was cleared in 2009, then the area was left idle until oil palms were planted in 2013. In the 16-day plot, you can see a temporal signature consistent with idle land from 2009-2013, followed by a signature consistent with oil palm growth. High resolution imagery from Google Earth confirms that the area was forested in 2006 and had been cleared but remained unplanted in 2012. Oil palms are visible in the 2015 and 2020 images.

A. Deforestation by fire

2006:

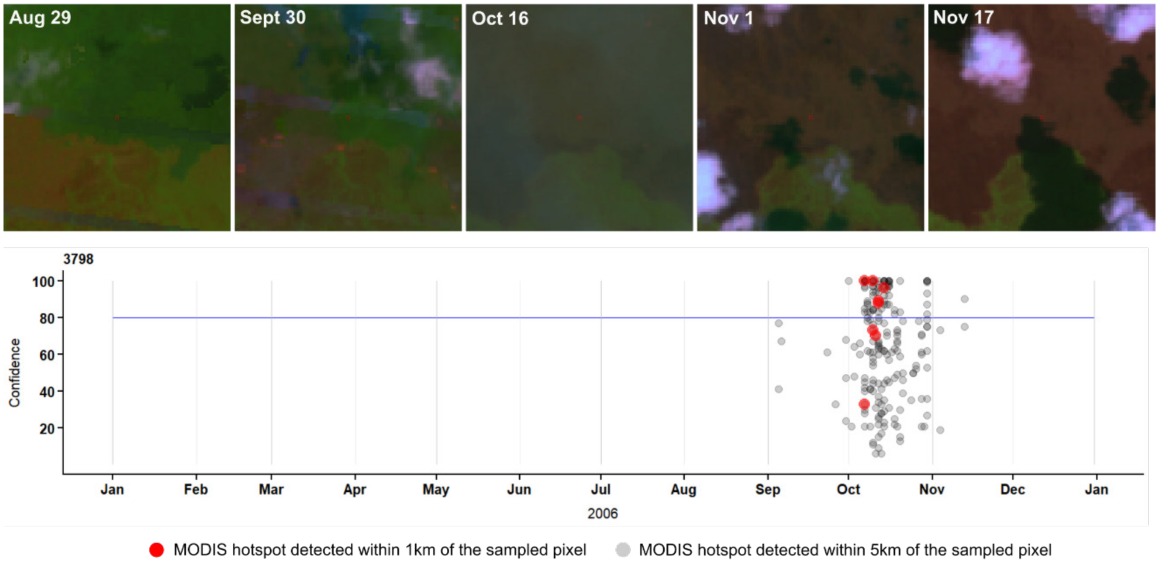

B. Mechanically cleared unplanted land

2002/2003: **2020:**

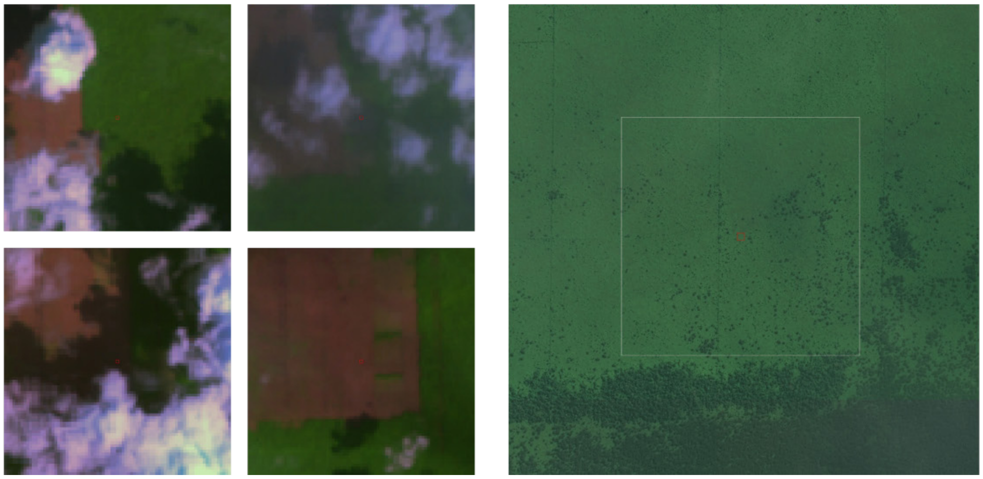

Fig. S9. Idle land clearing examples. In the fire clearing example (A), active fires (bright orange in the Landsat composites) are seen advancing towards the sampled pixel. By October 16, the pixel has been cleared and the image is obscured by haze from the fire. On November 1, the large, non-geometric clearing appears dark purple, which indicates ash. MODIS hotspots were detected in the area starting in early September, with most clustered in early October, coinciding with the clearing date. In the mechanical clearing example, the clearing is geometric in shape and appears pink, which is typical of bare ground. No MODIS hotspots were detected in the vicinity of the sampled pixel in either 2002 or 2003. In high resolution SPOT imagery from 2020, the land remains unplanted.

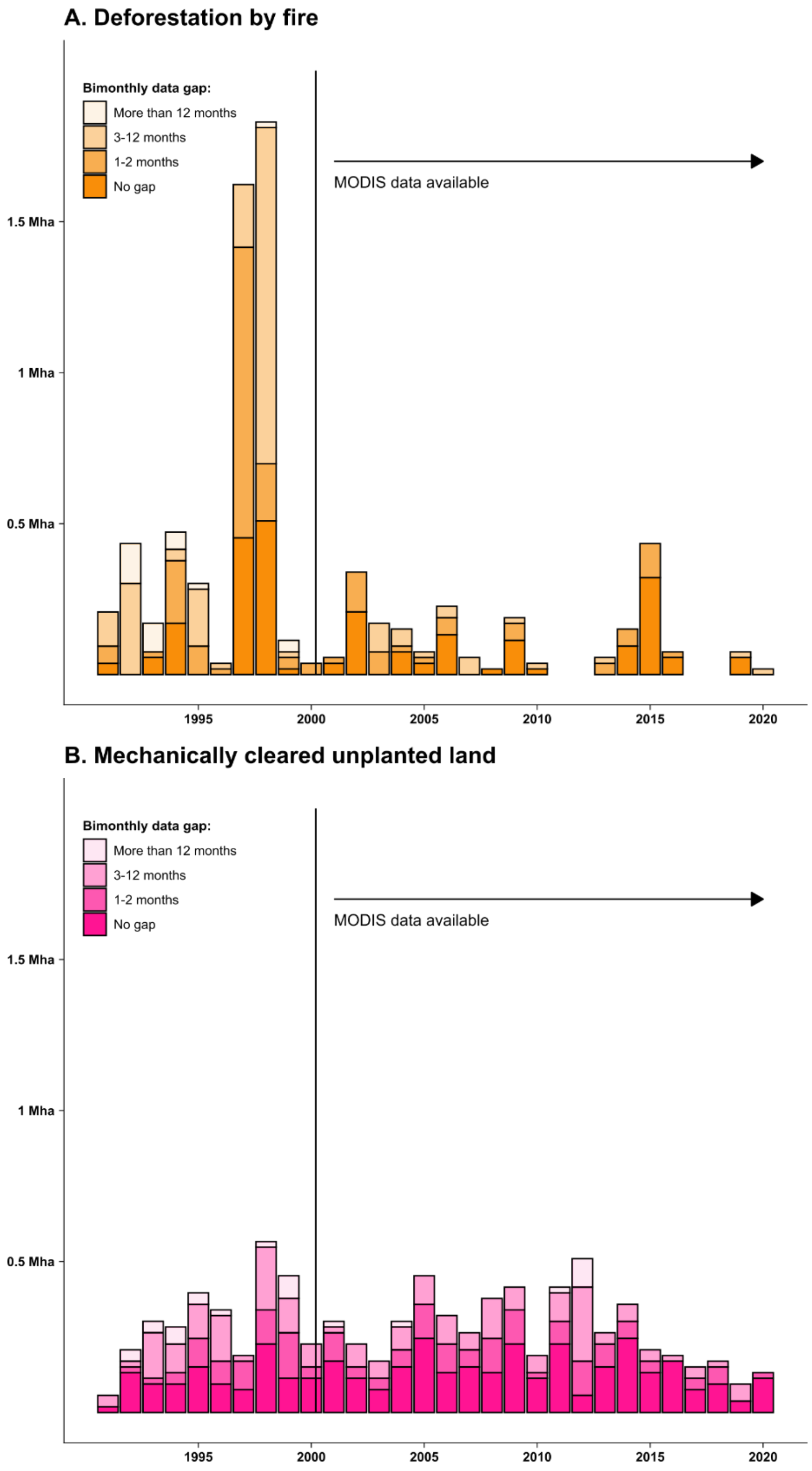

Fig. S10. Gap between the date deforestation was visible in a bimonthly composite image and the previous cloud-free forested bimonthly image date. Mechanically cleared unplanted land can be most accurately differentiated from forest loss due to fire in areas without gaps in the bimonthly imagery at the time the clearing event occurred. MODIS active fire data, available from 2000 onward, were also used to differentiate mechanically cleared land from land deforested by fire.

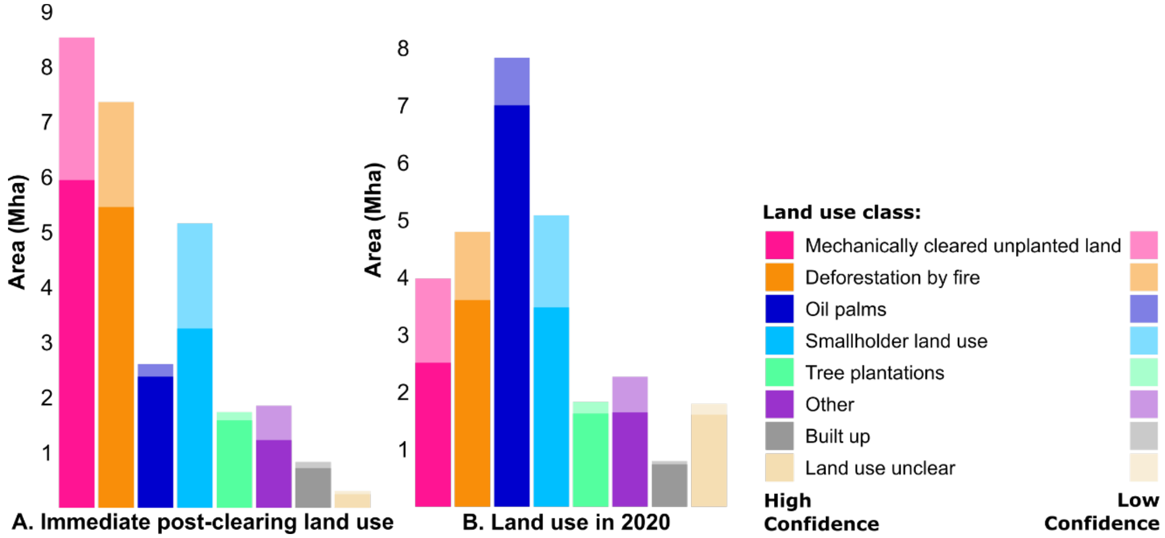

Fig. S11. Proportion of high and low confidence interpretations by driver. Confidence levels are displayed for the land use immediately (within 12 months) of forest clearing (A) and for the land use in 2020 (B).

Table S1. Estimated annual primary forest area (intact, degraded in 1990, and degraded after 1990) and forest loss area (Mha) nationally from 1990-2020. Standard errors are reported in parenthesis.

Year	Primary forest	Intact primary forest	Degraded after 1990	Degraded in 1990	Forest loss
**1990**	**115.4 (±0.83)**	88.98 (±0.86)	–	26.42 (±0.65)	–
**1991**	**114.83 (±0.83)**	87.94 (±0.86)	0.98 (±0.14)	25.91 (±0.64)	0.57 (±0.1)
**1992**	**113.7 (±0.83)**	86.2 (±0.86)	2.44 (±0.21)	25.06 (±0.63)	1.7 (±0.18)
**1993**	**112.83 (±0.83)**	84.54 (±0.86)	3.85 (±0.27)	24.44 (±0.63)	2.57 (±0.22)
**1994**	**111.59 (±0.83)**	82.58 (±0.86)	5.25 (±0.31)	23.76 (±0.62)	3.81 (±0.26)
**1995**	**110.42 (±0.84)**	80.69 (±0.85)	6.74 (±0.35)	22.99 (±0.61)	4.98 (±0.3)
**1996**	**109.34 (±0.84)**	79.03 (±0.85)	7.93 (±0.38)	22.38 (±0.6)	6.06 (±0.33)
**1997**	**106.83 (±0.84)**	74.82 (±0.85)	10.63 (±0.43)	21.38 (±0.59)	8.57 (±0.39)
**1998**	**103.76 (±0.85)**	70.5 (±0.84)	13.44 (±0.48)	19.82 (±0.57)	11.64 (±0.45)
**1999**	**102.6 (±0.85)**	68.55 (±0.84)	14.75 (±0.5)	19.31 (±0.57)	12.8 (±0.47)
**2000**	**101.98 (±0.85)**	67.06 (±0.83)	15.88 (±0.52)	19.04 (±0.56)	13.42 (±0.48)
**2001**	**101.3 (±0.85)**	65.51 (±0.83)	17.03 (±0.54)	18.76 (±0.56)	14.1 (±0.49)
**2002**	**100.3 (±0.85)**	64.08 (±0.82)	17.98 (±0.55)	18.25 (±0.55)	15.1 (±0.5)
**2003**	**99.66 (±0.85)**	63.15 (±0.82)	18.58 (±0.56)	17.93 (±0.55)	15.74 (±0.51)
**2004**	**98.75 (±0.85)**	61.85 (±0.82)	19.36 (±0.57)	17.55 (±0.54)	16.65 (±0.53)
**2005**	**97.79 (±0.84)**	61.06 (±0.81)	19.51 (±0.57)	17.23 (±0.54)	17.61 (±0.54)
**2006**	**96.62 (±0.84)**	60.09 (±0.81)	19.83 (±0.58)	16.7 (±0.53)	18.78 (±0.55)
**2007**	**95.83 (±0.84)**	59.17 (±0.81)	20.32 (±0.58)	16.34 (±0.53)	19.57 (±0.56)
**2008**	**95.02 (±0.84)**	58.32 (±0.8)	20.64 (±0.58)	16.06 (±0.52)	20.38 (±0.57)
**2009**	**94.02 (±0.84)**	57.66 (±0.8)	20.74 (±0.59)	15.62 (±0.52)	21.38 (±0.58)
**2010**	**93.45 (±0.84)**	56.96 (±0.8)	21.1 (±0.59)	15.4 (±0.51)	21.95 (±0.59)
**2011**	**92.64 (±0.84)**	56.47 (±0.8)	20.98 (±0.59)	15.19 (±0.51)	22.76 (±0.6)
**2012**	**91.81 (±0.84)**	55.86 (±0.8)	21.11 (±0.59)	14.83 (±0.5)	23.59 (±0.61)
**2013**	**91.22 (±0.84)**	54.79 (±0.79)	21.76 (±0.6)	14.68 (±0.5)	24.18 (±0.61)
**2014**	**90.28 (±0.84)**	53.8 (±0.79)	22 (±0.6)	14.47 (±0.5)	25.12 (±0.62)
**2015**	**89.24 (±0.84)**	52.37 (±0.79)	22.83 (±0.61)	14.04 (±0.49)	26.16 (±0.63)
**2016**	**88.49 (±0.84)**	51.42 (±0.78)	23.23 (±0.61)	13.83 (±0.49)	26.91 (±0.64)
**2017**	**88.09 (±0.84)**	50.57 (±0.78)	23.87 (±0.62)	13.64 (±0.48)	27.31 (±0.64)
**2018**	**87.77 (±0.83)**	49.99 (±0.78)	24.27 (±0.62)	13.51 (±0.48)	27.63 (±0.65)
**2019**	**87.35 (±0.83)**	49.1 (±0.77)	24.89 (±0.63)	13.36 (±0.48)	28.05 (±0.65)
**2020**	**87.03 (±0.83)**	48.61 (±0.77)	25.12 (±0.63)	13.3 (±0.48)	28.37 (±0.65)

Table S2. Land use transition five years after forest loss (A) and in 2020 (B). Standard errors are included in parentheses.

**A**	Unplanted land	Cleared by fire	Oil palm	Small-holder use	Tree plantations	Other clearing types	Built up land	Land use unclear	Total (±direct driver)
**Unplanted land**	***6.23 (±0.33)***	–	1.74 (±0.18)	0.08 (±0.04)	0.17 (±0.06)	0.19 (±0.06)	0.02 (±0.02)	0.11 (±0.05)	**8.53 (±0.39)**
**Cleared by fire**	–	***6.94 (±0.35)***	0.25 (±0.07)	0.04 (±0.03)	0.06 (±0.03)	0.08 (±0.04)	–	–	**7.36 (±0.36)**
**Oil palm**	–	–	***2.6 (±0.22)***	–	–	–	–	–	**2.6 (±0.22)**
**Small-holder use**	–	0.06 (±0.03)	–	***5.06 (±0.3)***	–	0.02 (±0.02)	–	0.02 (±0.02)	**5.16 (±0.31)**
**Tree plantations**	0.02 (±0.02)	0.02 (±0.02)	–	–	***1.7 (±0.18)***	–	–	–	**1.74 (±0.18)**
**Other clearing types**	–	0.04 (±0.03)	–	–	–	***1.81 (±0.18)***	–	–	**1.85 (±0.19)**
**Built up land**	0.02 (±0.02)	0.02 (±0.02)	–	–	–	0.02 (±0.02)	***0.77 (±0.12)***	–	**0.83 (±0.12)**
**Land use unclear**	–	–	–	–	–	–	–	***0.3 (±0.08)***	**0.3 (±0.08)**
**Total (±5 yrs.)**	**6.27 (±0.34)**	**7.08 (±0.35)**	**4.59 (±0.29)**	**5.17 (±0.31)**	**1.92 (±0.19)**	**2.11 (±0.2)**	**0.79 (±0.12)**	**0.43 (±0.09)**	**28.37 (±0.65)**

**B**	Unplanted land	Cleared by fire	Oil palm	Small-holder use	Tree plantations	Other clearing	Built up land	Land use unclear	Total (±direct driver)
**Unplanted land**	***3.89 (±0.27)***	–	3.42 (±0.25)	0.15 (±0.05)	0.19 (±0.06)	0.42 (±0.09)	0.04 (±0.03)	0.43 (±0.09)	**8.53 (±0.39)**
**Cleared by fire**	–	***4.68 (±0.29)***	1.53 (±0.17)	0.17 (±0.06)	0.3 (±0.08)	0.21 (±0.06)	0.06 (±0.03)	0.42 (±0.09)	**7.36 (±0.36)**
**Oil palm**	0.02 (±0.02)	–	***2.55 (±0.22)***	–	–	–	–	0.04 (±0.03)	**2.6 (±0.22)**
**Small-holder use**	0.02 (±0.02)	0.04 (±0.03)	0.17 (±0.06)	***4.72 (±0.29)***	–	0.08 (±0.04)	0.02 (±0.02)	0.11 (±0.05)	**5.16 (±0.31)**
**Tree plantations**	0.04 (±0.03)	0.02 (±0.02)	0.04 (±0.03)	–	***1.3 (±0.16)***	0.02 (±0.02)	–	0.32 (±0.08)	**1.74 (±0.18)**
**Other clearing types**	0.02 (±0.02)	0.02 (±0.02)	0.08 (±0.04)	0.04 (±0.03)	–	***1.51 (±0.17)***	0.02 (±0.02)	0.17 (±0.06)	**1.85 (±0.19)**
**Built up land**	–	0.04 (±0.03)	0.06 (±0.03)	–	0.04 (±0.03)	0.04 (±0.03)	***0.66 (±0.11)***	–	**0.83 (±0.12)**
**Land use unclear**	–	–	–	–	–	–	–	***0.3 (±0.08)***	**0.3 (±0.08)**
**Total (final land use)**	**3.98 (±0.27)**	**4.79 (±0.3)**	**7.83 (±0.37)**	**5.08 (±0.3)**	**1.83 (±0.18)**	**2.26 (±0.2)**	**0.79 (±0.12)**	**1.79 (±0.18)**	**28.37 (±0.65)**

Table S3. Mean forest disturbance area (Mha) by era; all clearing mechanisms (A) non-fire clearing only (B), and intact primary forest degradation (C). Standard errors reported in parentheses.

Region	Time period	All terrains	slopes >4° and/or elevations >1500 m	slopes ≤4° and/or elevations ≤1500 m
**A. All clearing mechanisms**				
**National**	1991-2020	0.95 (±0.02)	0.27 (±0.01)	0.67 (±0.02)
	2013-2016	0.83 (±0.06)	0.28 (±0.04)	0.55 (±0.05)
	2017-2020	0.36 (±0.04)	0.20 (±0.03)	0.17 (±0.03)
**Kalimantan**	1991-2020	0.4 (±0.01)	0.10 (±0.01)	0.31 (±0.01)
	2013-2016	0.34 (±0.04)	0.08 (±0.02)	0.26 (±0.03)
	2017-2020	0.16 (±0.03)	0.06 (±0.02)	0.1 (±0.02)
**Sumatra**	1991-2020	0.37 (±0.01)	0.08 (±0.01)	0.29 (±0.01)
	2013-2016	0.29 (±0.04)	0.09 (±0.02)	0.2 (±0.03)
	2017-2020	0.1 (±0.02)	0.06 (±0.02)	0.04 (±0.01)
**Papua**	1991-2020	0.06 (±0.01)	0.02 (±0.00)	0.04 (±0.00)
	2013-2016	0.09 (±0.02)	0.02 (±0.01)	0.06 (±0.02)
	2017-2020	0.02 (±0.01)	0.01 (±0.01)	0.01 (±0.01)
**B. Non-fire clearing**				
**National**	1991-2020	0.7 (±0.02)	0.24 (±0.01)	0.46 (±0.02)
	2013-2016	0.65 (±0.05)	0.25 (±0.03)	0.41 (±0.04)
	2017-2020	0.34 (±0.04)	0.18 (±0.03)	0.16 (±0.03)
**Kalimantan**	1991-2020	0.26 (±0.01)	0.08 (±0.01)	0.18 (±0.01)
	2013-2016	0.27 (±0.04)	0.08 (±0.02)	0.19 (±0.03)
	2017-2020	0.16 (±0.03)	0.06 (±0.02)	0.1 (±0.02)
**Sumatra**	1991-2020	0.3 (±0.01)	0.08 (±0.01)	0.22 (±0.01)
	2013-2016	0.23 (±0.03)	0.08 (±0.02)	0.14 (±0.03)
	2017-2020	0.09 (±0.02)	0.06 (±0.02)	0.03 (±0.01)
**Papua**	1991-2020	0.04 (±0.01)	0.01 (±0.00)	0.03 (±0.00)
	2013-2016	0.07 (±0.02)	0.02 (±0.01)	0.05 (±0.01)
	2017-2020	0.01 (±0.01)	0.00 (±0.00)	0.01 (±0.01)
**C. Intact primary forest degradation**				
**National**	1991-2020	1.19 (±0.02)	0.59 (±0.02)	0.6 (±0.02)
	2013-2016	1.02 (±0.07)	0.69 (±0.06)	0.33 (±0.04)
	2017-2020	0.64 (±0.05)	0.47 (±0.05)	0.17 (±0.03)
**Kalimantan**	1991-2020	0.38 (±0.01)	0.17 (±0.01)	0.21 (±0.01)
	2013-2016	0.25 (±0.03)	0.12 (±0.02)	0.13 (±0.02)
	2017-2020	0.17 (±0.03)	0.11 (±0.02)	0.05 (±0.02)
**Sumatra**	1991-2020	0.31 (±0.01)	0.13 (±0.01)	0.18 (±0.01)
	2013-2016	0.16 (±0.03)	0.1 (±0.02)	0.06 (±0.02)
	2017-2020	0.11 (±0.02)	0.08 (±0.02)	0.04 (±0.01)
**Papua**	1991-2020	0.31 (±0.01)	0.13 (±0.01)	0.18 (±0.01)
	2013-2016	0.35 (±0.04)	0.2 (±0.03)	0.14 (±0.03)
	2017-2020	0.24 (±0.03)	0.17 (±0.03)	0.07 (±0.02)

Table S4. Estimated primary forest and forest loss area (Mha) nationally in areas with slopes >4° and/or elevations >1500 meters. Standard errors are reported in parenthesis.

Year	Primary forest	Intact primary forest	Degraded after 1990	Degraded in 1990	Forest loss
**1990**	**65.24 (±0.87)**	52.98 (±0.82)	–	12.26 (±0.46)	–
**1991**	**65.09 (±0.87)**	52.49 (±0.82)	0.47 (±0.09)	12.13 (±0.46)	0.15 (±0.05)
**1992**	**64.92 (±0.87)**	52.02 (±0.82)	0.93 (±0.13)	11.98 (±0.46)	0.32 (±0.08)
**1993**	**64.75 (±0.86)**	51.58 (±0.81)	1.32 (±0.16)	11.85 (±0.46)	0.49 (±0.1)
**1994**	**64.47 (±0.86)**	50.88 (±0.81)	1.91 (±0.19)	11.68 (±0.45)	0.77 (±0.12)
**1995**	**64.22 (±0.86)**	50.35 (±0.81)	2.36 (±0.21)	11.51 (±0.45)	1.02 (±0.14)
**1996**	**63.92 (±0.86)**	49.77 (±0.81)	2.83 (±0.23)	11.32 (±0.45)	1.32 (±0.16)
**1997**	**63.45 (±0.86)**	48.15 (±0.8)	4.25 (±0.28)	11.06 (±0.44)	1.79 (±0.18)
**1998**	**62.66 (±0.86)**	46.47 (±0.79)	5.63 (±0.32)	10.57 (±0.43)	2.59 (±0.22)
**1999**	**62.2 (±0.86)**	45.39 (±0.78)	6.49 (±0.34)	10.32 (±0.43)	3.04 (±0.24)
**2000**	**61.9 (±0.86)**	44.71 (±0.78)	6.99 (±0.36)	10.21 (±0.42)	3.34 (±0.25)
**2001**	**61.64 (±0.86)**	43.9 (±0.77)	7.68 (±0.37)	10.05 (±0.42)	3.61 (±0.26)
**2002**	**61.3 (±0.85)**	43.31 (±0.77)	8.14 (±0.38)	9.85 (±0.42)	3.95 (±0.27)
**2003**	**61.11 (±0.85)**	42.88 (±0.77)	8.46 (±0.39)	9.77 (±0.42)	4.14 (±0.28)
**2004**	**60.82 (±0.85)**	42.18 (±0.77)	9.01 (±0.4)	9.64 (±0.41)	4.42 (±0.28)
**2005**	**60.52 (±0.85)**	41.74 (±0.76)	9.27 (±0.41)	9.51 (±0.41)	4.72 (±0.29)
**2006**	**60.16 (±0.85)**	41.23 (±0.76)	9.54 (±0.41)	9.39 (±0.41)	5.08 (±0.3)
**2007**	**59.97 (±0.85)**	40.59 (±0.76)	10.1 (±0.42)	9.28 (±0.41)	5.27 (±0.31)
**2008**	**59.71 (±0.85)**	40.12 (±0.75)	10.39 (±0.43)	9.2 (±0.4)	5.53 (±0.32)
**2009**	**59.41 (±0.85)**	39.78 (±0.75)	10.65 (±0.43)	8.98 (±0.4)	5.83 (±0.33)
**2010**	**59.3 (±0.85)**	39.44 (±0.75)	10.95 (±0.44)	8.9 (±0.4)	5.95 (±0.33)
**2011**	**59.01 (±0.85)**	39.12 (±0.75)	11.1 (±0.44)	8.79 (±0.4)	6.23 (±0.34)
**2012**	**58.92 (±0.84)**	38.91 (±0.74)	11.27 (±0.45)	8.73 (±0.39)	6.33 (±0.34)
**2013**	**58.67 (±0.84)**	38.25 (±0.74)	11.77 (±0.45)	8.66 (±0.39)	6.57 (±0.34)
**2014**	**58.41 (±0.84)**	37.7 (±0.74)	12.11 (±0.46)	8.6 (±0.39)	6.83 (±0.35)
**2015**	**58.09 (±0.84)**	36.63 (±0.73)	13.05 (±0.48)	8.41 (±0.39)	7.16 (±0.36)
**2016**	**57.8 (±0.84)**	36.06 (±0.73)	13.41 (±0.48)	8.34 (±0.39)	7.44 (±0.37)
**2017**	**57.58 (±0.84)**	35.4 (±0.72)	13.98 (±0.49)	8.2 (±0.38)	7.67 (±0.37)
**2018**	**57.41 (±0.84)**	35.06 (±0.72)	14.26 (±0.5)	8.09 (±0.38)	7.84 (±0.37)
**2019**	**57.18 (±0.84)**	34.28 (±0.71)	14.86 (±0.5)	8.03 (±0.38)	8.06 (±0.38)
**2020**	**57.01 (±0.84)**	34.04 (±0.71)	14.94 (±0.51)	8.03 (±0.38)	8.23 (±0.38)

Table S5. Estimated primary forest and forest loss area (Mha) nationally in areas with slopes ≤4° and/or elevations ≤1500 meters. Standard errors are reported in parenthesis.

Year	Primary forest	Intact primary forest	Degraded after 1990	Degraded in 1990	Forest loss
1990	**50.16 (±0.79)**	36 (±0.7)	–	14.16 (±0.49)	–
1991	**49.74 (±0.79)**	35.45 (±0.7)	0.51 (±0.1)	13.78 (±0.48)	0.42 (±0.09)
1992	**48.78 (±0.79)**	34.19 (±0.69)	1.51 (±0.17)	13.08 (±0.47)	1.38 (±0.16)
1993	**48.08 (±0.78)**	32.96 (±0.68)	2.53 (±0.22)	12.59 (±0.47)	2.08 (±0.2)
1994	**47.12 (±0.78)**	31.7 (±0.67)	3.34 (±0.25)	12.08 (±0.46)	3.04 (±0.24)
1995	**46.2 (±0.77)**	30.34 (±0.65)	4.38 (±0.28)	11.48 (±0.45)	3.96 (±0.27)
1996	**45.42 (±0.77)**	29.26 (±0.64)	5.1 (±0.3)	11.06 (±0.44)	4.74 (±0.29)
1997	**43.38 (±0.75)**	26.68 (±0.62)	6.38 (±0.34)	10.33 (±0.42)	6.77 (±0.35)
1998	**41.1 (±0.74)**	24.03 (±0.59)	7.82 (±0.37)	9.25 (±0.4)	9.06 (±0.4)
1999	**40.4 (±0.73)**	23.16 (±0.58)	8.25 (±0.38)	8.99 (±0.4)	9.76 (±0.41)
2000	**40.08 (±0.73)**	22.35 (±0.57)	8.89 (±0.4)	8.84 (±0.4)	10.08 (±0.42)
2001	**39.67 (±0.73)**	21.62 (±0.57)	9.35 (±0.41)	8.7 (±0.39)	10.49 (±0.43)
2002	**39.01 (±0.72)**	20.77 (±0.55)	9.84 (±0.42)	8.4 (±0.39)	11.15 (±0.44)
2003	**38.55 (±0.72)**	20.27 (±0.55)	10.12 (±0.42)	8.16 (±0.38)	11.61 (±0.45)
2004	**37.93 (±0.71)**	19.67 (±0.54)	10.35 (±0.43)	7.91 (±0.38)	12.23 (±0.46)
2005	**37.27 (±0.71)**	19.31 (±0.54)	10.24 (±0.42)	7.72 (±0.37)	12.89 (±0.47)
2006	**36.46 (±0.7)**	18.86 (±0.53)	10.29 (±0.43)	7.31 (±0.36)	13.7 (±0.48)
2007	**35.85 (±0.69)**	18.58 (±0.53)	10.22 (±0.42)	7.06 (±0.36)	14.3 (±0.49)
2008	**35.31 (±0.69)**	18.2 (±0.52)	10.25 (±0.42)	6.85 (±0.35)	14.85 (±0.5)
2009	**34.61 (±0.68)**	17.88 (±0.52)	10.09 (±0.42)	6.65 (±0.35)	15.55 (±0.51)
2010	**34.16 (±0.68)**	17.52 (±0.51)	10.14 (±0.42)	6.5 (±0.34)	16 (±0.51)
2011	**33.63 (±0.67)**	17.35 (±0.51)	9.88 (±0.42)	6.4 (±0.34)	16.53 (±0.52)
2012	**32.89 (±0.67)**	16.95 (±0.51)	9.84 (±0.41)	6.1 (±0.33)	17.27 (±0.53)
2013	**32.55 (±0.66)**	16.54 (±0.5)	9.99 (±0.42)	6.02 (±0.33)	17.61 (±0.53)
2014	**31.87 (±0.66)**	16.1 (±0.49)	9.9 (±0.42)	5.87 (±0.33)	18.29 (±0.54)
2015	**31.15 (±0.65)**	15.74 (±0.49)	9.78 (±0.41)	5.63 (±0.32)	19 (±0.55)
2016	**30.68 (±0.65)**	15.37 (±0.48)	9.82 (±0.41)	5.5 (±0.32)	19.48 (±0.56)
2017	**30.51 (±0.64)**	15.18 (±0.48)	9.9 (±0.41)	5.44 (±0.31)	19.65 (±0.56)
2018	**30.36 (±0.64)**	14.93 (±0.48)	10.01 (±0.42)	5.42 (±0.31)	19.8 (±0.56)
2019	**30.17 (±0.64)**	14.82 (±0.47)	10.03 (±0.42)	5.32 (±0.31)	19.99 (±0.56)
2020	**30.02 (±0.64)**	14.57 (±0.47)	10.18 (±0.42)	5.27 (±0.31)	20.14 (±0.56)

Table S6. Estimated primary forest and forest loss area (Mha) in Kalimantan. Standard errors are reported in parenthesis.

Year	Island	Primary forest	Intact forest	Degraded after 1990	Degraded in 1990	Forest loss
1990	Kalimantan	**37.6 (±0.46)**	26.47 (±0.5)	–	11.13 (±0.41)	**–**
1991	Kalimantan	**37.24 (±0.46)**	26 (±0.5)	0.42 (±0.09)	10.83 (±0.4)	**0.36 (±0.08)**
1992	Kalimantan	**36.84 (±0.46)**	25.45 (±0.5)	0.92 (±0.13)	10.47 (±0.4)	**0.75 (±0.12)**
1993	Kalimantan	**36.47 (±0.47)**	24.73 (±0.5)	1.57 (±0.17)	10.17 (±0.39)	**1.13 (±0.14)**
1994	Kalimantan	**36.18 (±0.47)**	24.11 (±0.5)	2.08 (±0.19)	10 (±0.39)	**1.41 (±0.16)**
1995	Kalimantan	**35.69 (±0.47)**	23.24 (±0.5)	2.81 (±0.22)	9.64 (±0.39)	**1.91 (±0.19)**
1996	Kalimantan	**35.26 (±0.48)**	22.62 (±0.5)	3.26 (±0.24)	9.38 (±0.38)	**2.34 (±0.21)**
1997	Kalimantan	**33.79 (±0.48)**	21.05 (±0.49)	3.92 (±0.26)	8.81 (±0.37)	**3.81 (±0.26)**
1998	Kalimantan	**32.22 (±0.49)**	19.2 (±0.48)	4.98 (±0.29)	8.04 (±0.36)	**5.38 (±0.3)**
1999	Kalimantan	**31.88 (±0.49)**	18.75 (±0.48)	5.3 (±0.3)	7.83 (±0.36)	**5.72 (±0.31)**
2000	Kalimantan	**31.66 (±0.49)**	18.05 (±0.47)	5.89 (±0.31)	7.72 (±0.35)	**5.94 (±0.32)**
2001	Kalimantan	**31.39 (±0.49)**	17.51 (±0.47)	6.28 (±0.32)	7.6 (±0.35)	**6.21 (±0.32)**
2002	Kalimantan	**30.96 (±0.5)**	17.09 (±0.47)	6.53 (±0.33)	7.34 (±0.35)	**6.64 (±0.33)**
2003	Kalimantan	**30.75 (±0.5)**	16.94 (±0.47)	6.58 (±0.33)	7.23 (±0.34)	**6.85 (±0.34)**
2004	Kalimantan	**30.45 (±0.5)**	16.54 (±0.46)	6.9 (±0.34)	7 (±0.34)	**7.15 (±0.34)**
2005	Kalimantan	**30.15 (±0.5)**	16.21 (±0.46)	7.06 (±0.34)	6.89 (±0.34)	**7.45 (±0.35)**
2006	Kalimantan	**29.52 (±0.5)**	15.9 (±0.46)	7.15 (±0.34)	6.47 (±0.33)	**8.07 (±0.36)**
2007	Kalimantan	**29.13 (±0.5)**	15.6 (±0.46)	7.24 (±0.34)	6.28 (±0.32)	**8.47 (±0.37)**
2008	Kalimantan	**28.79 (±0.5)**	15.41 (±0.45)	7.26 (±0.34)	6.11 (±0.32)	**8.81 (±0.37)**
2009	Kalimantan	**28.41 (±0.5)**	15.26 (±0.45)	7.15 (±0.34)	6 (±0.32)	**9.19 (±0.38)**
2010	Kalimantan	**28.18 (±0.5)**	15.09 (±0.45)	7.17 (±0.34)	5.92 (±0.32)	**9.41 (±0.38)**
2011	Kalimantan	**27.79 (±0.5)**	14.96 (±0.45)	7.06 (±0.34)	5.77 (±0.31)	**9.81 (±0.39)**
2012	Kalimantan	**27.51 (±0.5)**	14.79 (±0.45)	7.06 (±0.34)	5.66 (±0.31)	**10.09 (±0.39)**
2013	Kalimantan	**27.26 (±0.5)**	14.41 (±0.45)	7.28 (±0.34)	5.57 (±0.31)	**10.34 (±0.4)**
2014	Kalimantan	**26.96 (±0.5)**	14.02 (±0.44)	7.43 (±0.35)	5.51 (±0.31)	**10.64 (±0.4)**
2015	Kalimantan	**26.45 (±0.5)**	13.83 (±0.44)	7.36 (±0.35)	5.26 (±0.3)	**11.15 (±0.41)**
2016	Kalimantan	**26.13 (±0.5)**	13.62 (±0.44)	7.38 (±0.35)	5.13 (±0.3)	**11.47 (±0.41)**
2017	Kalimantan	**25.92 (±0.5)**	13.36 (±0.43)	7.53 (±0.35)	5.04 (±0.29)	**11.68 (±0.41)**
2018	Kalimantan	**25.83 (±0.5)**	13.09 (±0.43)	7.73 (±0.35)	5 (±0.29)	**11.77 (±0.42)**
2019	Kalimantan	**25.62 (±0.5)**	12.87 (±0.43)	7.87 (±0.36)	4.89 (±0.29)	**11.98 (±0.42)**
2020	Kalimantan	**25.49 (±0.5)**	12.79 (±0.43)	7.83 (±0.36)	4.87 (±0.29)	**12.11 (±0.42)**

Table S7. Estimated primary forest and forest loss area (Mha) in Sumatra. Standard errors are reported in parenthesis.

Year	Island	Primary forest	Intact forest	Degraded after 1990	Degraded in 1990	Forest loss
1990	Sumatra	**22.91 (±0.47)**	16.61 (±0.45)	–	6.3 (±0.32)	**–**
1991	Sumatra	**22.78 (±0.47)**	16.29 (±0.45)	0.32 (±0.08)	6.17 (±0.32)	**0.13 (±0.05)**
1992	Sumatra	**22.08 (±0.47)**	15.36 (±0.44)	1.04 (±0.14)	5.68 (±0.31)	**0.83 (±0.12)**
1993	Sumatra	**21.68 (±0.47)**	14.8 (±0.44)	1.45 (±0.16)	5.44 (±0.3)	**1.23 (±0.15)**
1994	Sumatra	**21.06 (±0.47)**	14.02 (±0.43)	1.89 (±0.18)	5.15 (±0.29)	**1.85 (±0.18)**
1995	Sumatra	**20.55 (±0.47)**	13.44 (±0.43)	2.25 (±0.2)	4.87 (±0.29)	**2.36 (±0.21)**
1996	Sumatra	**20.08 (±0.47)**	12.89 (±0.42)	2.57 (±0.21)	4.62 (±0.28)	**2.83 (±0.22)**
1997	Sumatra	**19.46 (±0.47)**	12.27 (±0.41)	2.81 (±0.22)	4.38 (±0.27)	**3.45 (±0.25)**
1998	Sumatra	**18.19 (±0.46)**	10.83 (±0.4)	3.59 (±0.25)	3.77 (±0.26)	**4.72 (±0.28)**
1999	Sumatra	**17.83 (±0.46)**	10.17 (±0.39)	3.96 (±0.26)	3.7 (±0.25)	**5.08 (±0.29)**
2000	Sumatra	**17.53 (±0.46)**	9.76 (±0.38)	4.15 (±0.27)	3.62 (±0.25)	**5.38 (±0.3)**
2001	Sumatra	**17.29 (±0.46)**	9.32 (±0.38)	4.45 (±0.28)	3.51 (±0.25)	**5.62 (±0.31)**
2002	Sumatra	**16.98 (±0.45)**	9 (±0.37)	4.57 (±0.28)	3.42 (±0.24)	**5.93 (±0.31)**
2003	Sumatra	**16.76 (±0.45)**	8.76 (±0.37)	4.68 (±0.28)	3.32 (±0.24)	**6.15 (±0.32)**
2004	Sumatra	**16.32 (±0.45)**	8.4 (±0.36)	4.7 (±0.28)	3.23 (±0.24)	**6.59 (±0.33)**
2005	Sumatra	**15.8 (±0.45)**	8.19 (±0.36)	4.51 (±0.28)	3.1 (±0.23)	**7.11 (±0.34)**
2006	Sumatra	**15.32 (±0.44)**	7.96 (±0.35)	4.38 (±0.27)	2.98 (±0.23)	**7.59 (±0.35)**
2007	Sumatra	**15 (±0.44)**	7.78 (±0.35)	4.36 (±0.27)	2.87 (±0.23)	**7.91 (±0.35)**
2008	Sumatra	**14.61 (±0.44)**	7.55 (±0.35)	4.28 (±0.27)	2.77 (±0.22)	**8.3 (±0.36)**
2009	Sumatra	**14.23 (±0.43)**	7.4 (±0.34)	4.21 (±0.27)	2.62 (±0.22)	**8.68 (±0.37)**
2010	Sumatra	**13.98 (±0.43)**	7.27 (±0.34)	4.17 (±0.27)	2.55 (±0.21)	**8.93 (±0.37)**
2011	Sumatra	**13.7 (±0.43)**	7.11 (±0.34)	4.1 (±0.27)	2.49 (±0.21)	**9.21 (±0.37)**
2012	Sumatra	**13.29 (±0.43)**	7 (±0.34)	3.98 (±0.26)	2.3 (±0.2)	**9.62 (±0.38)**
2013	Sumatra	**13.08 (±0.42)**	6.87 (±0.33)	3.94 (±0.26)	2.26 (±0.2)	**9.83 (±0.38)**
2014	Sumatra	**12.63 (±0.42)**	6.81 (±0.33)	3.68 (±0.25)	2.13 (±0.2)	**10.29 (±0.39)**
2015	Sumatra	**12.4 (±0.42)**	6.61 (±0.33)	3.74 (±0.25)	2.06 (±0.19)	**10.51 (±0.39)**
2016	Sumatra	**12.13 (±0.41)**	6.34 (±0.32)	3.77 (±0.26)	2.02 (±0.19)	**10.78 (±0.4)**
2017	Sumatra	**12.04 (±0.41)**	6.19 (±0.32)	3.85 (±0.26)	2 (±0.19)	**10.87 (±0.4)**
2018	Sumatra	**11.89 (±0.41)**	6.06 (±0.32)	3.89 (±0.26)	1.94 (±0.19)	**11.02 (±0.4)**
2019	Sumatra	**11.8 (±0.41)**	5.93 (±0.31)	3.93 (±0.26)	1.94 (±0.19)	**11.12 (±0.4)**
2020	Sumatra	**11.72 (±0.41)**	5.83 (±0.31)	3.96 (±0.26)	1.92 (±0.19)	**11.19 (±0.4)**

Table S8. Estimated primary forest and forest loss area (Mha) in Papua. Standard errors are reported in parenthesis.

Year	Island	Primary forest	Intact forest	Degraded after 1990	Degraded in 1990	Forest loss
1990	Papua	**35.23 (±0.31)**	31.34 (±0.37)	–	3.9 (±0.26)	**–**
1991	Papua	**35.21 (±0.31)**	31.24 (±0.38)	0.09 (±0.04)	3.88 (±0.26)	**0.02 (±0.02)**
1992	Papua	**35.17 (±0.31)**	31.03 (±0.38)	0.26 (±0.07)	3.88 (±0.26)	**0.06 (±0.03)**
1993	Papua	**35.14 (±0.31)**	30.75 (±0.38)	0.53 (±0.1)	3.86 (±0.26)	**0.09 (±0.04)**
1994	Papua	**35.1 (±0.31)**	30.48 (±0.39)	0.79 (±0.12)	3.82 (±0.26)	**0.13 (±0.05)**
1995	Papua	**35.08 (±0.31)**	30.24 (±0.39)	1.04 (±0.14)	3.8 (±0.26)	**0.15 (±0.05)**
1996	Papua	**35.04 (±0.31)**	29.97 (±0.39)	1.29 (±0.15)	3.78 (±0.25)	**0.19 (±0.06)**
1997	Papua	**34.8 (±0.32)**	28.69 (±0.4)	2.4 (±0.21)	3.71 (±0.25)	**0.43 (±0.09)**
1998	Papua	**34.72 (±0.32)**	27.91 (±0.41)	3.16 (±0.23)	3.65 (±0.25)	**0.51 (±0.1)**
1999	Papua	**34.53 (±0.32)**	27.33 (±0.42)	3.63 (±0.25)	3.57 (±0.25)	**0.7 (±0.11)**
2000	Papua	**34.51 (±0.32)**	27.04 (±0.42)	3.9 (±0.26)	3.57 (±0.25)	**0.72 (±0.12)**
2001	Papua	**34.46 (±0.32)**	26.7 (±0.42)	4.18 (±0.27)	3.57 (±0.25)	**0.78 (±0.12)**
2002	Papua	**34.42 (±0.32)**	26.31 (±0.42)	4.56 (±0.28)	3.56 (±0.25)	**0.81 (±0.12)**
2003	Papua	**34.38 (±0.33)**	25.98 (±0.42)	4.86 (±0.28)	3.54 (±0.25)	**0.85 (±0.13)**
2004	Papua	**34.34 (±0.33)**	25.7 (±0.43)	5.12 (±0.29)	3.52 (±0.25)	**0.89 (±0.13)**
2005	Papua	**34.34 (±0.33)**	25.57 (±0.43)	5.26 (±0.29)	3.52 (±0.25)	**0.89 (±0.13)**
2006	Papua	**34.3 (±0.33)**	25.38 (±0.43)	5.41 (±0.3)	3.52 (±0.25)	**0.93 (±0.13)**
2007	Papua	**34.27 (±0.33)**	25.17 (±0.43)	5.6 (±0.3)	3.5 (±0.25)	**0.96 (±0.13)**
2008	Papua	**34.27 (±0.33)**	24.89 (±0.43)	5.88 (±0.31)	3.5 (±0.25)	**0.96 (±0.13)**
2009	Papua	**34.17 (±0.33)**	24.6 (±0.43)	6.13 (±0.31)	3.44 (±0.24)	**1.06 (±0.14)**
2010	Papua	**34.13 (±0.33)**	24.28 (±0.43)	6.43 (±0.32)	3.42 (±0.24)	**1.1 (±0.14)**
2011	Papua	**34.02 (±0.33)**	24.13 (±0.43)	6.47 (±0.32)	3.42 (±0.24)	**1.21 (±0.15)**
2012	Papua	**33.95 (±0.33)**	23.94 (±0.43)	6.6 (±0.32)	3.4 (±0.24)	**1.29 (±0.15)**
2013	Papua	**33.85 (±0.34)**	23.64 (±0.44)	6.83 (±0.33)	3.39 (±0.24)	**1.38 (±0.16)**
2014	Papua	**33.78 (±0.34)**	23.2 (±0.44)	7.19 (±0.33)	3.39 (±0.24)	**1.46 (±0.16)**
2015	Papua	**33.68 (±0.34)**	22.79 (±0.44)	7.53 (±0.34)	3.37 (±0.24)	**1.55 (±0.17)**
2016	Papua	**33.61 (±0.34)**	22.49 (±0.44)	7.75 (±0.34)	3.37 (±0.24)	**1.63 (±0.17)**
2017	Papua	**33.59 (±0.34)**	22.18 (±0.44)	8.04 (±0.35)	3.37 (±0.24)	**1.65 (±0.17)**
2018	Papua	**33.57 (±0.34)**	22.07 (±0.44)	8.13 (±0.35)	3.37 (±0.24)	**1.66 (±0.17)**
2019	Papua	**33.53 (±0.34)**	21.71 (±0.44)	8.47 (±0.36)	3.35 (±0.24)	**1.7 (±0.18)**
2020	Papua	**33.53 (±0.34)**	21.52 (±0.44)	8.66 (±0.36)	3.35 (±0.24)	**1.7 (±0.18)**

Table S9. Estimated primary forest and forest loss area (Mha) in Sulawesi. Standard errors are reported in parenthesis.

Year	Island	Primary forest	Intact forest	Degraded after 1990	Degraded in 1990	Forest loss
1990	Sulawesi	**11.15 (±0.29)**	9.21 (±0.3)	–	1.94 (±0.18)	**–**
1991	Sulawesi	**11.13 (±0.29)**	9.19 (±0.3)	0.02 (±0.02)	1.92 (±0.18)	**0.02 (±0.02)**
1992	Sulawesi	**11.13 (±0.29)**	9.19 (±0.3)	0.02 (±0.02)	1.92 (±0.18)	**0.02 (±0.02)**
1993	Sulawesi	**11.11 (±0.29)**	9.15 (±0.3)	0.06 (±0.03)	1.91 (±0.18)	**0.04 (±0.03)**
1994	Sulawesi	**10.85 (±0.29)**	8.87 (±0.3)	0.25 (±0.07)	1.74 (±0.17)	**0.3 (±0.07)**
1995	Sulawesi	**10.79 (±0.29)**	8.72 (±0.3)	0.38 (±0.08)	1.7 (±0.17)	**0.36 (±0.08)**
1996	Sulawesi	**10.68 (±0.29)**	8.55 (±0.3)	0.49 (±0.09)	1.64 (±0.17)	**0.47 (±0.09)**
1997	Sulawesi	**10.59 (±0.29)**	8.06 (±0.29)	0.94 (±0.13)	1.58 (±0.17)	**0.57 (±0.1)**
1998	Sulawesi	**10.42 (±0.29)**	7.85 (±0.29)	1.11 (±0.14)	1.45 (±0.16)	**0.74 (±0.12)**
1999	Sulawesi	**10.23 (±0.3)**	7.66 (±0.29)	1.21 (±0.15)	1.36 (±0.15)	**0.92 (±0.13)**
2000	Sulawesi	**10.17 (±0.3)**	7.59 (±0.29)	1.28 (±0.15)	1.3 (±0.15)	**0.98 (±0.13)**
2001	Sulawesi	**10.08 (±0.3)**	7.51 (±0.29)	1.3 (±0.15)	1.26 (±0.15)	**1.08 (±0.14)**
2002	Sulawesi	**9.89 (±0.3)**	7.34 (±0.29)	1.38 (±0.16)	1.17 (±0.14)	**1.26 (±0.15)**
2003	Sulawesi	**9.77 (±0.3)**	7.19 (±0.29)	1.47 (±0.16)	1.11 (±0.14)	**1.38 (±0.16)**
2004	Sulawesi	**9.66 (±0.3)**	6.96 (±0.29)	1.6 (±0.17)	1.09 (±0.14)	**1.49 (±0.16)**
2005	Sulawesi	**9.57 (±0.3)**	6.91 (±0.29)	1.6 (±0.17)	1.06 (±0.14)	**1.58 (±0.17)**
2006	Sulawesi	**9.55 (±0.3)**	6.74 (±0.28)	1.75 (±0.17)	1.06 (±0.14)	**1.6 (±0.17)**
2007	Sulawesi	**9.55 (±0.3)**	6.55 (±0.28)	1.94 (±0.18)	1.06 (±0.14)	**1.6 (±0.17)**
2008	Sulawesi	**9.47 (±0.3)**	6.45 (±0.28)	1.98 (±0.18)	1.04 (±0.14)	**1.68 (±0.17)**
2009	Sulawesi	**9.36 (±0.3)**	6.45 (±0.28)	1.96 (±0.18)	0.94 (±0.13)	**1.79 (±0.17)**
2010	Sulawesi	**9.32 (±0.3)**	6.4 (±0.28)	2.02 (±0.18)	0.91 (±0.13)	**1.83 (±0.18)**
2011	Sulawesi	**9.3 (±0.3)**	6.36 (±0.28)	2.04 (±0.19)	0.91 (±0.13)	**1.85 (±0.18)**
2012	Sulawesi	**9.28 (±0.3)**	6.3 (±0.28)	2.09 (±0.19)	0.89 (±0.13)	**1.87 (±0.18)**
2013	Sulawesi	**9.25 (±0.3)**	6.09 (±0.28)	2.26 (±0.19)	0.89 (±0.13)	**1.91 (±0.18)**
2014	Sulawesi	**9.15 (±0.3)**	6.02 (±0.28)	2.26 (±0.19)	0.87 (±0.13)	**2 (±0.18)**
2015	Sulawesi	**9.04 (±0.3)**	5.57 (±0.27)	2.64 (±0.21)	0.83 (±0.12)	**2.11 (±0.19)**
2016	Sulawesi	**8.96 (±0.3)**	5.47 (±0.27)	2.68 (±0.21)	0.81 (±0.12)	**2.19 (±0.19)**
2017	Sulawesi	**8.91 (±0.3)**	5.4 (±0.27)	2.75 (±0.21)	0.75 (±0.12)	**2.25 (±0.19)**
2018	Sulawesi	**8.87 (±0.3)**	5.34 (±0.27)	2.79 (±0.21)	0.74 (±0.12)	**2.28 (±0.19)**
2019	Sulawesi	**8.85 (±0.3)**	5.23 (±0.27)	2.89 (±0.21)	0.74 (±0.12)	**2.3 (±0.2)**
2020	Sulawesi	**8.81 (±0.3)**	5.15 (±0.27)	2.92 (±0.22)	0.74 (±0.12)	**2.34 (±0.2)**

Table S10. Estimated primary forest and forest loss area (Mha) in Maluku, Java, and Nusa Tenggara. Standard errors are reported in parenthesis.

Year	Island	Primary forest	Intact forest	Degraded after 1990	Degraded in 1990	Forest loss
1990	Other	**8.51 (±0.27)**	5.36 (±0.25)	–	3.15 (±0.22)	**–**
1991	Other	**8.47 (±0.27)**	5.23 (±0.25)	0.13 (±0.05)	3.11 (±0.22)	**0.04 (±0.03)**
1992	Other	**8.47 (±0.27)**	5.17 (±0.25)	0.19 (±0.06)	3.11 (±0.22)	**0.04 (±0.03)**
1993	Other	**8.43 (±0.27)**	5.11 (±0.25)	0.25 (±0.07)	3.07 (±0.22)	**0.08 (±0.04)**
1994	Other	**8.39 (±0.27)**	5.1 (±0.25)	0.25 (±0.07)	3.05 (±0.22)	**0.11 (±0.05)**
1995	Other	**8.3 (±0.27)**	5.06 (±0.25)	0.27 (±0.07)	2.98 (±0.22)	**0.21 (±0.06)**
1996	Other	**8.28 (±0.27)**	5 (±0.25)	0.32 (±0.08)	2.96 (±0.22)	**0.23 (±0.07)**
1997	Other	**8.21 (±0.27)**	4.76 (±0.25)	0.55 (±0.1)	2.9 (±0.21)	**0.3 (±0.08)**
1998	Other	**8.21 (±0.27)**	4.7 (±0.25)	0.61 (±0.1)	2.9 (±0.21)	**0.3 (±0.08)**
1999	Other	**8.13 (±0.27)**	4.64 (±0.24)	0.64 (±0.11)	2.84 (±0.21)	**0.38 (±0.08)**
2000	Other	**8.11 (±0.27)**	4.62 (±0.24)	0.66 (±0.11)	2.83 (±0.21)	**0.4 (±0.09)**
2001	Other	**8.09 (±0.27)**	4.47 (±0.24)	0.81 (±0.12)	2.81 (±0.21)	**0.42 (±0.09)**
2002	Other	**8.05 (±0.27)**	4.34 (±0.24)	0.95 (±0.13)	2.77 (±0.21)	**0.46 (±0.09)**
2003	Other	**8 (±0.27)**	4.28 (±0.24)	0.98 (±0.13)	2.73 (±0.21)	**0.51 (±0.1)**
2004	Other	**7.98 (±0.27)**	4.24 (±0.24)	1.02 (±0.13)	2.71 (±0.21)	**0.53 (±0.1)**
2005	Other	**7.94 (±0.27)**	4.19 (±0.24)	1.08 (±0.14)	2.67 (±0.21)	**0.57 (±0.1)**
2006	Other	**7.92 (±0.27)**	4.11 (±0.24)	1.14 (±0.14)	2.67 (±0.21)	**0.59 (±0.1)**
2007	Other	**7.88 (±0.27)**	4.07 (±0.24)	1.17 (±0.14)	2.64 (±0.21)	**0.63 (±0.11)**
2008	Other	**7.88 (±0.27)**	4.02 (±0.23)	1.23 (±0.15)	2.64 (±0.21)	**0.63 (±0.11)**
2009	Other	**7.84 (±0.27)**	3.94 (±0.23)	1.29 (±0.15)	2.62 (±0.21)	**0.66 (±0.11)**
2010	Other	**7.83 (±0.27)**	3.92 (±0.23)	1.31 (±0.15)	2.6 (±0.2)	**0.68 (±0.11)**
2011	Other	**7.83 (±0.27)**	3.9 (±0.23)	1.33 (±0.15)	2.6 (±0.2)	**0.68 (±0.11)**
2012	Other	**7.79 (±0.27)**	3.83 (±0.23)	1.38 (±0.15)	2.58 (±0.2)	**0.72 (±0.11)**
2013	Other	**7.79 (±0.27)**	3.77 (±0.23)	1.44 (±0.16)	2.58 (±0.2)	**0.72 (±0.11)**
2014	Other	**7.77 (±0.27)**	3.75 (±0.23)	1.44 (±0.16)	2.58 (±0.2)	**0.74 (±0.12)**
2015	Other	**7.67 (±0.27)**	3.58 (±0.23)	1.57 (±0.16)	2.52 (±0.2)	**0.83 (±0.12)**
2016	Other	**7.66 (±0.27)**	3.5 (±0.23)	1.65 (±0.17)	2.5 (±0.2)	**0.85 (±0.12)**
2017	Other	**7.64 (±0.27)**	3.45 (±0.22)	1.7 (±0.17)	2.48 (±0.2)	**0.87 (±0.13)**
2018	Other	**7.62 (±0.27)**	3.43 (±0.22)	1.72 (±0.17)	2.46 (±0.2)	**0.89 (±0.13)**
2019	Other	**7.56 (±0.27)**	3.37 (±0.22)	1.74 (±0.17)	2.45 (±0.2)	**0.95 (±0.13)**
2020	Other	**7.48 (±0.27)**	3.31 (±0.22)	1.74 (±0.17)	2.43 (±0.2)	**1.02 (±0.14)**

Table S11. Estimated primary forest and forest loss area (Mha) in Kalimantan in areas with slopes >4° and/or elevations >1500 meters. Standard errors are reported in parenthesis.

Year	Island	Primary forest	Intact forest	Degraded after 1990	Degraded in 1990	Forest loss
1990	Kalimantan	**20.22 (±0.49)**	16.28 (±0.46)	–	3.94 (±0.26)	**–**
1991	Kalimantan	**20.17 (±0.49)**	16.04 (±0.46)	0.23 (±0.07)	3.91 (±0.26)	**0.06 (±0.03)**
1992	Kalimantan	**20.07 (±0.49)**	15.92 (±0.46)	0.34 (±0.08)	3.81 (±0.26)	**0.15 (±0.05)**
1993	Kalimantan	**19.98 (±0.49)**	15.75 (±0.46)	0.51 (±0.1)	3.72 (±0.26)	**0.25 (±0.07)**
1994	Kalimantan	**19.94 (±0.49)**	15.58 (±0.46)	0.68 (±0.11)	3.68 (±0.25)	**0.28 (±0.07)**
1995	Kalimantan	**19.87 (±0.49)**	15.36 (±0.45)	0.89 (±0.13)	3.62 (±0.25)	**0.36 (±0.08)**
1996	Kalimantan	**19.77 (±0.48)**	15.15 (±0.45)	1.06 (±0.14)	3.57 (±0.25)	**0.45 (±0.09)**
1997	Kalimantan	**19.58 (±0.48)**	14.6 (±0.45)	1.53 (±0.17)	3.45 (±0.25)	**0.64 (±0.11)**
1998	Kalimantan	**19.24 (±0.48)**	13.77 (±0.44)	2.19 (±0.2)	3.28 (±0.24)	**0.98 (±0.13)**
1999	Kalimantan	**19.17 (±0.48)**	13.55 (±0.44)	2.38 (±0.21)	3.24 (±0.24)	**1.06 (±0.14)**
2000	Kalimantan	**19.04 (±0.48)**	13.24 (±0.43)	2.58 (±0.22)	3.21 (±0.24)	**1.19 (±0.15)**
2001	Kalimantan	**18.94 (±0.48)**	12.89 (±0.43)	2.91 (±0.23)	3.15 (±0.24)	**1.28 (±0.15)**
2002	Kalimantan	**18.83 (±0.48)**	12.77 (±0.43)	3 (±0.23)	3.06 (±0.23)	**1.4 (±0.16)**
2003	Kalimantan	**18.79 (±0.48)**	12.68 (±0.43)	3.09 (±0.23)	3.02 (±0.23)	**1.43 (±0.16)**
2004	Kalimantan	**18.64 (±0.48)**	12.45 (±0.42)	3.26 (±0.24)	2.92 (±0.23)	**1.58 (±0.17)**
2005	Kalimantan	**18.53 (±0.48)**	12.3 (±0.42)	3.36 (±0.24)	2.87 (±0.23)	**1.7 (±0.18)**
2006	Kalimantan	**18.34 (±0.48)**	12.19 (±0.42)	3.4 (±0.24)	2.75 (±0.22)	**1.89 (±0.19)**
2007	Kalimantan	**18.24 (±0.48)**	12.07 (±0.42)	3.47 (±0.25)	2.7 (±0.22)	**1.98 (±0.19)**
2008	Kalimantan	**18.15 (±0.48)**	11.96 (±0.42)	3.53 (±0.25)	2.66 (±0.22)	**2.08 (±0.19)**
2009	Kalimantan	**18.11 (±0.48)**	11.89 (±0.42)	3.58 (±0.25)	2.64 (±0.22)	**2.11 (±0.2)**
2010	Kalimantan	**18.07 (±0.47)**	11.77 (±0.42)	3.68 (±0.25)	2.62 (±0.22)	**2.15 (±0.2)**
2011	Kalimantan	**17.87 (±0.47)**	11.7 (±0.42)	3.64 (±0.25)	2.53 (±0.21)	**2.36 (±0.21)**
2012	Kalimantan	**17.87 (±0.47)**	11.7 (±0.42)	3.64 (±0.25)	2.53 (±0.21)	**2.36 (±0.21)**
2013	Kalimantan	**17.81 (±0.47)**	11.56 (±0.41)	3.75 (±0.26)	2.49 (±0.21)	**2.41 (±0.21)**
2014	Kalimantan	**17.73 (±0.47)**	11.34 (±0.41)	3.92 (±0.26)	2.47 (±0.21)	**2.49 (±0.21)**
2015	Kalimantan	**17.6 (±0.47)**	11.26 (±0.41)	3.96 (±0.26)	2.38 (±0.21)	**2.62 (±0.22)**
2016	Kalimantan	**17.53 (±0.47)**	11.19 (±0.41)	3.98 (±0.26)	2.36 (±0.21)	**2.7 (±0.22)**
2017	Kalimantan	**17.43 (±0.47)**	11 (±0.41)	4.13 (±0.27)	2.3 (±0.2)	**2.79 (±0.22)**
2018	Kalimantan	**17.41 (±0.47)**	10.9 (±0.4)	4.23 (±0.27)	2.28 (±0.2)	**2.81 (±0.22)**
2019	Kalimantan	**17.32 (±0.47)**	10.7 (±0.4)	4.4 (±0.28)	2.23 (±0.2)	**2.91 (±0.23)**
2020	Kalimantan	**17.28 (±0.47)**	10.68 (±0.4)	4.38 (±0.28)	2.23 (±0.2)	**2.94 (±0.23)**

Table S12. Estimated primary forest and forest loss area (Mha) in Sumatra in areas with slopes >4° and/or elevations >1500 meters. Standard errors are reported in parenthesis.

Year	Island	Primary forest	Intact forest	Degraded after 1990	Degraded in 1990	Forest loss
1990	Sumatra	**10.85 (±0.4)**	8.78 (±0.37)	–	2.08 (±0.19)	**–**
1991	Sumatra	**10.83 (±0.4)**	8.66 (±0.37)	0.11 (±0.05)	2.06 (±0.19)	**0.02 (±0.02)**
1992	Sumatra	**10.76 (±0.4)**	8.45 (±0.36)	0.3 (±0.08)	2 (±0.19)	**0.09 (±0.04)**
1993	Sumatra	**10.72 (±0.4)**	8.27 (±0.36)	0.45 (±0.09)	2 (±0.19)	**0.13 (±0.05)**
1994	Sumatra	**10.68 (±0.4)**	8.08 (±0.36)	0.6 (±0.11)	2 (±0.19)	**0.17 (±0.06)**
1995	Sumatra	**10.62 (±0.39)**	8 (±0.35)	0.66 (±0.11)	1.96 (±0.19)	**0.23 (±0.07)**
1996	Sumatra	**10.49 (±0.39)**	7.87 (±0.35)	0.75 (±0.12)	1.87 (±0.18)	**0.36 (±0.08)**
1997	Sumatra	**10.4 (±0.39)**	7.66 (±0.35)	0.91 (±0.13)	1.83 (±0.18)	**0.45 (±0.09)**
1998	Sumatra	**10.1 (±0.39)**	7.19 (±0.34)	1.28 (±0.15)	1.62 (±0.17)	**0.75 (±0.12)**
1999	Sumatra	**10 (±0.39)**	6.78 (±0.33)	1.62 (±0.17)	1.6 (±0.17)	**0.85 (±0.13)**
2000	Sumatra	**9.89 (±0.38)**	6.59 (±0.33)	1.72 (±0.18)	1.59 (±0.17)	**0.96 (±0.13)**
2001	Sumatra	**9.81 (±0.38)**	6.47 (±0.32)	1.79 (±0.18)	1.55 (±0.17)	**1.04 (±0.14)**
2002	Sumatra	**9.79 (±0.38)**	6.4 (±0.32)	1.85 (±0.18)	1.55 (±0.17)	**1.06 (±0.14)**
2003	Sumatra	**9.76 (±0.38)**	6.32 (±0.32)	1.89 (±0.18)	1.55 (±0.17)	**1.09 (±0.14)**
2004	Sumatra	**9.74 (±0.38)**	6.21 (±0.32)	1.98 (±0.19)	1.55 (±0.17)	**1.11 (±0.14)**
2005	Sumatra	**9.62 (±0.38)**	6.15 (±0.32)	1.96 (±0.19)	1.51 (±0.17)	**1.23 (±0.15)**
2006	Sumatra	**9.51 (±0.38)**	6.02 (±0.32)	1.98 (±0.19)	1.51 (±0.17)	**1.34 (±0.16)**
2007	Sumatra	**9.47 (±0.38)**	5.83 (±0.31)	2.13 (±0.2)	1.51 (±0.17)	**1.38 (±0.16)**
2008	Sumatra	**9.38 (±0.38)**	5.7 (±0.31)	2.19 (±0.2)	1.49 (±0.17)	**1.47 (±0.16)**
2009	Sumatra	**9.21 (±0.37)**	5.62 (±0.31)	2.21 (±0.2)	1.38 (±0.16)	**1.64 (±0.17)**
2010	Sumatra	**9.17 (±0.37)**	5.61 (±0.31)	2.21 (±0.2)	1.36 (±0.16)	**1.68 (±0.17)**
2011	Sumatra	**9.12 (±0.37)**	5.51 (±0.3)	2.26 (±0.2)	1.34 (±0.16)	**1.74 (±0.18)**
2012	Sumatra	**9.04 (±0.37)**	5.47 (±0.3)	2.26 (±0.2)	1.3 (±0.15)	**1.81 (±0.18)**
2013	Sumatra	**8.93 (±0.37)**	5.4 (±0.3)	2.25 (±0.2)	1.28 (±0.15)	**1.92 (±0.19)**
2014	Sumatra	**8.81 (±0.37)**	5.38 (±0.3)	2.19 (±0.2)	1.25 (±0.15)	**2.04 (±0.19)**
2015	Sumatra	**8.79 (±0.37)**	5.21 (±0.3)	2.34 (±0.2)	1.25 (±0.15)	**2.06 (±0.19)**
2016	Sumatra	**8.68 (±0.37)**	5.08 (±0.29)	2.38 (±0.21)	1.23 (±0.15)	**2.17 (±0.2)**
2017	Sumatra	**8.62 (±0.37)**	4.98 (±0.29)	2.43 (±0.21)	1.21 (±0.15)	**2.23 (±0.2)**
2018	Sumatra	**8.53 (±0.36)**	4.89 (±0.29)	2.49 (±0.21)	1.15 (±0.15)	**2.32 (±0.2)**
2019	Sumatra	**8.47 (±0.36)**	4.77 (±0.28)	2.55 (±0.21)	1.15 (±0.15)	**2.38 (±0.21)**
2020	Sumatra	**8.44 (±0.36)**	4.74 (±0.28)	2.55 (±0.21)	1.15 (±0.15)	**2.42 (±0.21)**

Table S13. Estimated primary forest and forest loss area (Mha) in Papua in areas with slopes >4° and/or elevations >1500 meters. Standard errors are reported in parenthesis.

Year	Island	Primary forest	Intact forest	Degraded after 1990	Degraded in 1990	Forest loss
1990	Papua	**17.34 (±0.44)**	15.09 (±0.42)	–	2.25 (±0.2)	**–**
1991	Papua	**17.32 (±0.44)**	15.07 (±0.42)	0.02 (±0.02)	2.23 (±0.2)	**0.02 (±0.02)**
1992	Papua	**17.32 (±0.44)**	14.94 (±0.42)	0.15 (±0.05)	2.23 (±0.2)	**0.02 (±0.02)**
1993	Papua	**17.32 (±0.44)**	14.92 (±0.42)	0.17 (±0.06)	2.23 (±0.2)	**0.02 (±0.02)**
1994	Papua	**17.32 (±0.44)**	14.83 (±0.42)	0.26 (±0.07)	2.23 (±0.2)	**0.02 (±0.02)**
1995	Papua	**17.32 (±0.44)**	14.75 (±0.42)	0.34 (±0.08)	2.23 (±0.2)	**0.02 (±0.02)**
1996	Papua	**17.3 (±0.44)**	14.68 (±0.42)	0.4 (±0.09)	2.23 (±0.2)	**0.04 (±0.03)**
1997	Papua	**17.25 (±0.44)**	14.41 (±0.42)	0.64 (±0.11)	2.19 (±0.2)	**0.09 (±0.04)**
1998	Papua	**17.25 (±0.44)**	14.28 (±0.42)	0.78 (±0.12)	2.19 (±0.2)	**0.09 (±0.04)**
1999	Papua	**17.17 (±0.43)**	14.05 (±0.42)	0.98 (±0.13)	2.14 (±0.2)	**0.17 (±0.06)**
2000	Papua	**17.17 (±0.43)**	13.94 (±0.42)	1.1 (±0.14)	2.14 (±0.2)	**0.17 (±0.06)**
2001	Papua	**17.17 (±0.43)**	13.81 (±0.42)	1.23 (±0.15)	2.14 (±0.2)	**0.17 (±0.06)**
2002	Papua	**17.13 (±0.43)**	13.63 (±0.42)	1.38 (±0.16)	2.12 (±0.19)	**0.21 (±0.06)**
2003	Papua	**17.11 (±0.43)**	13.56 (±0.41)	1.44 (±0.16)	2.12 (±0.19)	**0.23 (±0.07)**
2004	Papua	**17.1 (±0.43)**	13.43 (±0.41)	1.57 (±0.17)	2.1 (±0.19)	**0.25 (±0.07)**
2005	Papua	**17.1 (±0.43)**	13.31 (±0.41)	1.68 (±0.17)	2.1 (±0.19)	**0.25 (±0.07)**
2006	Papua	**17.08 (±0.43)**	13.26 (±0.41)	1.72 (±0.18)	2.1 (±0.19)	**0.26 (±0.07)**
2007	Papua	**17.06 (±0.43)**	13.12 (±0.41)	1.85 (±0.18)	2.08 (±0.19)	**0.28 (±0.07)**
2008	Papua	**17.06 (±0.43)**	13.03 (±0.41)	1.95 (±0.19)	2.08 (±0.19)	**0.28 (±0.07)**
2009	Papua	**17.02 (±0.43)**	12.9 (±0.41)	2.08 (±0.19)	2.04 (±0.19)	**0.32 (±0.08)**
2010	Papua	**17 (±0.43)**	12.76 (±0.41)	2.21 (±0.2)	2.02 (±0.19)	**0.34 (±0.08)**
2011	Papua	**16.98 (±0.43)**	12.67 (±0.41)	2.29 (±0.2)	2.02 (±0.19)	**0.36 (±0.08)**
2012	Papua	**16.98 (±0.43)**	12.59 (±0.41)	2.36 (±0.21)	2.02 (±0.19)	**0.36 (±0.08)**
2013	Papua	**16.94 (±0.43)**	12.41 (±0.4)	2.53 (±0.21)	2 (±0.19)	**0.4 (±0.09)**
2014	Papua	**16.94 (±0.43)**	12.18 (±0.4)	2.76 (±0.22)	2 (±0.19)	**0.4 (±0.09)**
2015	Papua	**16.91 (±0.43)**	11.93 (±0.4)	2.99 (±0.23)	1.99 (±0.19)	**0.43 (±0.09)**
2016	Papua	**16.89 (±0.43)**	11.74 (±0.4)	3.16 (±0.23)	1.99 (±0.19)	**0.45 (±0.09)**
2017	Papua	**16.87 (±0.43)**	11.5 (±0.4)	3.39 (±0.24)	1.99 (±0.19)	**0.47 (±0.09)**
2018	Papua	**16.87 (±0.43)**	11.42 (±0.39)	3.46 (±0.24)	1.99 (±0.19)	**0.47 (±0.09)**
2019	Papua	**16.85 (±0.43)**	11.14 (±0.39)	3.73 (±0.25)	1.99 (±0.19)	**0.49 (±0.1)**
2020	Papua	**16.85 (±0.43)**	11.06 (±0.39)	3.8 (±0.26)	1.99 (±0.19)	**0.49 (±0.1)**

Table S14. Estimated primary forest and forest loss area (Mha) in Sulawesi in areas with slopes >4° and/or elevations >1500 meters. Standard errors are reported in parenthesis.

Year	Island	Primary forest	Intact	Degraded after 1990	Degraded in 1990	Forest loss
1990	Sulawesi	**9.92 (±0.3)**	8.49 (±0.3)	–	1.43 (±0.16)	**–**
1991	Sulawesi	**9.91 (±0.3)**	8.47 (±0.3)	0.02 (±0.02)	1.42 (±0.16)	**0.02 (±0.02)**
1992	Sulawesi	**9.91 (±0.3)**	8.47 (±0.3)	0.02 (±0.02)	1.42 (±0.16)	**0.02 (±0.02)**
1993	Sulawesi	**9.91 (±0.3)**	8.45 (±0.3)	0.04 (±0.03)	1.42 (±0.16)	**0.02 (±0.02)**
1994	Sulawesi	**9.72 (±0.3)**	8.23 (±0.29)	0.19 (±0.06)	1.3 (±0.15)	**0.21 (±0.06)**
1995	Sulawesi	**9.68 (±0.3)**	8.11 (±0.29)	0.28 (±0.07)	1.28 (±0.15)	**0.25 (±0.07)**
1996	Sulawesi	**9.64 (±0.3)**	7.96 (±0.29)	0.42 (±0.09)	1.26 (±0.15)	**0.28 (±0.07)**
1997	Sulawesi	**9.57 (±0.3)**	7.55 (±0.29)	0.79 (±0.12)	1.23 (±0.15)	**0.36 (±0.08)**
1998	Sulawesi	**9.42 (±0.3)**	7.36 (±0.29)	0.94 (±0.13)	1.11 (±0.14)	**0.51 (±0.1)**
1999	Sulawesi	**9.26 (±0.3)**	7.19 (±0.29)	1.04 (±0.14)	1.04 (±0.14)	**0.66 (±0.11)**
2000	Sulawesi	**9.23 (±0.3)**	7.11 (±0.29)	1.11 (±0.14)	1 (±0.13)	**0.7 (±0.11)**
2001	Sulawesi	**9.15 (±0.3)**	7.06 (±0.29)	1.13 (±0.14)	0.96 (±0.13)	**0.77 (±0.12)**
2002	Sulawesi	**9.02 (±0.3)**	6.94 (±0.29)	1.17 (±0.14)	0.91 (±0.13)	**0.91 (±0.13)**
2003	Sulawesi	**8.98 (±0.3)**	6.79 (±0.29)	1.28 (±0.15)	0.91 (±0.13)	**0.94 (±0.13)**
2004	Sulawesi	**8.91 (±0.3)**	6.59 (±0.28)	1.42 (±0.16)	0.91 (±0.13)	**1.02 (±0.13)**
2005	Sulawesi	**8.85 (±0.3)**	6.53 (±0.28)	1.43 (±0.16)	0.89 (±0.13)	**1.08 (±0.14)**
2006	Sulawesi	**8.83 (±0.3)**	6.4 (±0.28)	1.55 (±0.16)	0.89 (±0.13)	**1.09 (±0.14)**
2007	Sulawesi	**8.83 (±0.3)**	6.23 (±0.28)	1.72 (±0.17)	0.89 (±0.13)	**1.09 (±0.14)**
2008	Sulawesi	**8.76 (±0.3)**	6.13 (±0.28)	1.75 (±0.17)	0.87 (±0.13)	**1.17 (±0.14)**
2009	Sulawesi	**8.7 (±0.3)**	6.13 (±0.28)	1.75 (±0.17)	0.81 (±0.12)	**1.23 (±0.15)**
2010	Sulawesi	**8.7 (±0.3)**	6.08 (±0.28)	1.81 (±0.18)	0.81 (±0.12)	**1.23 (±0.15)**
2011	Sulawesi	**8.7 (±0.3)**	6.04 (±0.28)	1.85 (±0.18)	0.81 (±0.12)	**1.23 (±0.15)**
2012	Sulawesi	**8.68 (±0.3)**	6 (±0.28)	1.89 (±0.18)	0.79 (±0.12)	**1.25 (±0.15)**
2013	Sulawesi	**8.64 (±0.3)**	5.79 (±0.27)	2.06 (±0.19)	0.79 (±0.12)	**1.28 (±0.15)**
2014	Sulawesi	**8.59 (±0.3)**	5.74 (±0.27)	2.06 (±0.19)	0.79 (±0.12)	**1.34 (±0.15)**
2015	Sulawesi	**8.51 (±0.3)**	5.3 (±0.27)	2.45 (±0.2)	0.75 (±0.12)	**1.42 (±0.16)**
2016	Sulawesi	**8.45 (±0.3)**	5.21 (±0.27)	2.51 (±0.2)	0.74 (±0.12)	**1.47 (±0.16)**
2017	Sulawesi	**8.4 (±0.3)**	5.13 (±0.26)	2.59 (±0.21)	0.68 (±0.11)	**1.53 (±0.16)**
2018	Sulawesi	**8.36 (±0.29)**	5.08 (±0.26)	2.62 (±0.21)	0.66 (±0.11)	**1.57 (±0.16)**
2019	Sulawesi	**8.34 (±0.29)**	4.96 (±0.26)	2.72 (±0.21)	0.66 (±0.11)	**1.58 (±0.17)**
2020	Sulawesi	**8.3 (±0.29)**	4.91 (±0.26)	2.74 (±0.21)	0.66 (±0.11)	**1.62 (±0.17)**

Table S15. Estimated primary forest and forest loss area (Mha) in Maluku, Java, and Nusa Tenggara in areas with slopes >4° and/or elevations >1500 meters. Standard errors are reported in parenthesis.

Year	Island	Primary forest	Intact	Degraded after 1990	Degraded in 1990	Forest loss
1990	Other	**6.9 (±0.28)**	4.34 (±0.24)	–	2.56 (±0.21)	**–**
1991	Other	**6.86 (±0.28)**	4.25 (±0.24)	0.09 (±0.04)	2.52 (±0.2)	**0.04 (±0.03)**
1992	Other	**6.86 (±0.28)**	4.23 (±0.24)	0.11 (±0.05)	2.52 (±0.2)	**0.04 (±0.03)**
1993	Other	**6.83 (±0.28)**	4.19 (±0.24)	0.15 (±0.05)	2.49 (±0.2)	**0.08 (±0.04)**
1994	Other	**6.81 (±0.28)**	4.17 (±0.24)	0.17 (±0.06)	2.47 (±0.2)	**0.1 (±0.04)**
1995	Other	**6.73 (±0.28)**	4.13 (±0.24)	0.19 (±0.06)	2.41 (±0.2)	**0.17 (±0.06)**
1996	Other	**6.71 (±0.28)**	4.11 (±0.24)	0.21 (±0.06)	2.39 (±0.2)	**0.19 (±0.06)**
1997	Other	**6.65 (±0.28)**	3.92 (±0.24)	0.38 (±0.08)	2.35 (±0.2)	**0.25 (±0.07)**
1998	Other	**6.65 (±0.28)**	3.87 (±0.24)	0.44 (±0.09)	2.35 (±0.2)	**0.25 (±0.07)**
1999	Other	**6.6 (±0.28)**	3.83 (±0.23)	0.47 (±0.09)	2.3 (±0.2)	**0.3 (±0.08)**
2000	Other	**6.58 (±0.28)**	3.83 (±0.23)	0.47 (±0.09)	2.28 (±0.2)	**0.32 (±0.08)**
2001	Other	**6.56 (±0.28)**	3.68 (±0.23)	0.63 (±0.11)	2.26 (±0.19)	**0.34 (±0.08)**
2002	Other	**6.52 (±0.28)**	3.56 (±0.23)	0.74 (±0.12)	2.22 (±0.19)	**0.38 (±0.08)**
2003	Other	**6.47 (±0.28)**	3.53 (±0.23)	0.76 (±0.12)	2.18 (±0.19)	**0.44 (±0.09)**
2004	Other	**6.45 (±0.28)**	3.51 (±0.23)	0.78 (±0.12)	2.16 (±0.19)	**0.46 (±0.09)**
2005	Other	**6.43 (±0.27)**	3.45 (±0.23)	0.83 (±0.12)	2.14 (±0.19)	**0.47 (±0.09)**
2006	Other	**6.41 (±0.27)**	3.37 (±0.22)	0.89 (±0.13)	2.14 (±0.19)	**0.49 (±0.1)**
2007	Other	**6.37 (±0.27)**	3.34 (±0.22)	0.93 (±0.13)	2.11 (±0.19)	**0.53 (±0.1)**
2008	Other	**6.37 (±0.27)**	3.3 (±0.22)	0.97 (±0.13)	2.11 (±0.19)	**0.53 (±0.1)**
2009	Other	**6.37 (±0.27)**	3.24 (±0.22)	1.02 (±0.13)	2.11 (±0.19)	**0.53 (±0.1)**
2010	Other	**6.35 (±0.27)**	3.22 (±0.22)	1.04 (±0.14)	2.09 (±0.19)	**0.55 (±0.1)**
2011	Other	**6.35 (±0.27)**	3.2 (±0.22)	1.06 (±0.14)	2.09 (±0.19)	**0.55 (±0.1)**
2012	Other	**6.35 (±0.27)**	3.15 (±0.22)	1.12 (±0.14)	2.09 (±0.19)	**0.55 (±0.1)**
2013	Other	**6.35 (±0.27)**	3.09 (±0.22)	1.17 (±0.14)	2.09 (±0.19)	**0.55 (±0.1)**
2014	Other	**6.33 (±0.27)**	3.07 (±0.22)	1.17 (±0.14)	2.09 (±0.19)	**0.57 (±0.1)**
2015	Other	**6.28 (±0.27)**	2.92 (±0.21)	1.31 (±0.15)	2.05 (±0.19)	**0.63 (±0.11)**
2016	Other	**6.26 (±0.27)**	2.84 (±0.21)	1.38 (±0.15)	2.03 (±0.19)	**0.65 (±0.11)**
2017	Other	**6.26 (±0.27)**	2.79 (±0.21)	1.44 (±0.16)	2.03 (±0.19)	**0.65 (±0.11)**
2018	Other	**6.24 (±0.27)**	2.77 (±0.21)	1.46 (±0.16)	2.01 (±0.18)	**0.66 (±0.11)**
2019	Other	**6.2 (±0.27)**	2.71 (±0.21)	1.48 (±0.16)	2.01 (±0.18)	**0.7 (±0.11)**
2020	Other	**6.14 (±0.27)**	2.65 (±0.21)	1.48 (±0.16)	2.01 (±0.18)	**0.76 (±0.12)**

Table S16. Estimated primary forest and forest loss area (Mha) in Kalimantan in areas with slopes ≤4° and/or elevations ≤1500 meters. Standard errors are reported in parenthesis.

Year	Island	Primary forest	Intact	Degraded after 1990	Degraded in 1990	Forest loss
1990	Kalimantan	**17.37 (±0.47)**	10.19 (±0.39)	–	7.19 (±0.34)	**–**
1991	Kalimantan	**17.07 (±0.47)**	9.96 (±0.39)	0.19 (±0.06)	6.92 (±0.34)	**0.3 (±0.08)**
1992	Kalimantan	**16.77 (±0.47)**	9.53 (±0.38)	0.58 (±0.1)	6.66 (±0.33)	**0.6 (±0.11)**
1993	Kalimantan	**16.49 (±0.46)**	8.98 (±0.38)	1.06 (±0.14)	6.45 (±0.33)	**0.89 (±0.13)**
1994	Kalimantan	**16.24 (±0.46)**	8.53 (±0.37)	1.4 (±0.16)	6.32 (±0.32)	**1.13 (±0.14)**
1995	Kalimantan	**15.83 (±0.46)**	7.89 (±0.36)	1.92 (±0.19)	6.02 (±0.32)	**1.55 (±0.17)**
1996	Kalimantan	**15.49 (±0.46)**	7.47 (±0.35)	2.21 (±0.2)	5.81 (±0.31)	**1.89 (±0.19)**
1997	Kalimantan	**14.21 (±0.44)**	6.45 (±0.33)	2.4 (±0.21)	5.36 (±0.3)	**3.17 (±0.24)**
1998	Kalimantan	**12.98 (±0.43)**	5.43 (±0.3)	2.79 (±0.22)	4.75 (±0.29)	**4.4 (±0.28)**
1999	Kalimantan	**12.72 (±0.43)**	5.21 (±0.3)	2.92 (±0.23)	4.58 (±0.28)	**4.66 (±0.28)**
2000	Kalimantan	**12.62 (±0.43)**	4.81 (±0.29)	3.3 (±0.24)	4.51 (±0.28)	**4.75 (±0.29)**
2001	Kalimantan	**12.45 (±0.42)**	4.62 (±0.28)	3.38 (±0.24)	4.45 (±0.28)	**4.92 (±0.29)**
2002	Kalimantan	**12.13 (±0.42)**	4.32 (±0.27)	3.53 (±0.25)	4.28 (±0.27)	**5.24 (±0.3)**
2003	Kalimantan	**11.96 (±0.42)**	4.26 (±0.27)	3.49 (±0.25)	4.21 (±0.27)	**5.41 (±0.3)**
2004	Kalimantan	**11.81 (±0.42)**	4.09 (±0.27)	3.64 (±0.25)	4.07 (±0.27)	**5.57 (±0.31)**
2005	Kalimantan	**11.62 (±0.41)**	3.91 (±0.26)	3.7 (±0.25)	4.02 (±0.26)	**5.75 (±0.31)**
2006	Kalimantan	**11.19 (±0.41)**	3.72 (±0.26)	3.75 (±0.26)	3.72 (±0.26)	**6.19 (±0.32)**
2007	Kalimantan	**10.89 (±0.4)**	3.53 (±0.25)	3.77 (±0.26)	3.58 (±0.25)	**6.49 (±0.33)**
2008	Kalimantan	**10.64 (±0.4)**	3.45 (±0.25)	3.74 (±0.26)	3.45 (±0.25)	**6.73 (±0.33)**
2009	Kalimantan	**10.3 (±0.4)**	3.38 (±0.24)	3.57 (±0.25)	3.36 (±0.24)	**7.07 (±0.34)**
2010	Kalimantan	**10.11 (±0.39)**	3.32 (±0.24)	3.49 (±0.25)	3.3 (±0.24)	**7.26 (±0.34)**
2011	Kalimantan	**9.92 (±0.39)**	3.26 (±0.24)	3.41 (±0.25)	3.24 (±0.24)	**7.45 (±0.35)**
2012	Kalimantan	**9.64 (±0.39)**	3.09 (±0.23)	3.41 (±0.25)	3.13 (±0.24)	**7.73 (±0.35)**
2013	Kalimantan	**9.45 (±0.38)**	2.85 (±0.23)	3.53 (±0.25)	3.08 (±0.23)	**7.92 (±0.36)**
2014	Kalimantan	**9.23 (±0.38)**	2.68 (±0.22)	3.51 (±0.25)	3.04 (±0.23)	**8.15 (±0.36)**
2015	Kalimantan	**8.85 (±0.37)**	2.57 (±0.21)	3.4 (±0.24)	2.89 (±0.23)	**8.53 (±0.37)**
2016	Kalimantan	**8.6 (±0.37)**	2.43 (±0.21)	3.4 (±0.24)	2.77 (±0.22)	**8.77 (±0.37)**
2017	Kalimantan	**8.49 (±0.37)**	2.36 (±0.21)	3.4 (±0.24)	2.74 (±0.22)	**8.89 (±0.37)**
2018	Kalimantan	**8.41 (±0.37)**	2.19 (±0.2)	3.51 (±0.25)	2.72 (±0.22)	**8.96 (±0.38)**
2019	Kalimantan	**8.3 (±0.36)**	2.17 (±0.2)	3.47 (±0.25)	2.66 (±0.22)	**9.07 (±0.38)**
2020	Kalimantan	**8.21 (±0.36)**	2.11 (±0.2)	3.45 (±0.25)	2.64 (±0.22)	**9.17 (±0.38)**

Table S17. Estimated primary forest and forest loss area (Mha) in Sumatra in areas with slopes ≤4° and/or elevations ≤1500 meters. Standard errors are reported in parenthesis.

Year	Island	Primary forest	Intact	Degraded after 1990	Degraded in 1990	Forest loss
1990	Sumatra	**12.06 (±0.41)**	7.83 (±0.35)	–	4.23 (±0.27)	**–**
1991	Sumatra	**11.95 (±0.41)**	7.62 (±0.35)	0.21 (±0.06)	4.11 (±0.27)	**0.11 (±0.05)**
1992	Sumatra	**11.32 (±0.4)**	6.91 (±0.33)	0.74 (±0.12)	3.68 (±0.25)	**0.74 (±0.12)**
1993	Sumatra	**10.96 (±0.4)**	6.53 (±0.33)	1 (±0.14)	3.43 (±0.25)	**1.09 (±0.14)**
1994	Sumatra	**10.38 (±0.39)**	5.94 (±0.31)	1.28 (±0.15)	3.15 (±0.24)	**1.68 (±0.17)**
1995	Sumatra	**9.93 (±0.39)**	5.44 (±0.3)	1.59 (±0.17)	2.91 (±0.23)	**2.13 (±0.2)**
1996	Sumatra	**9.59 (±0.38)**	5.02 (±0.29)	1.81 (±0.18)	2.76 (±0.22)	**2.47 (±0.21)**
1997	Sumatra	**9.06 (±0.37)**	4.6 (±0.28)	1.91 (±0.19)	2.55 (±0.21)	**3 (±0.23)**
1998	Sumatra	**8.1 (±0.36)**	3.64 (±0.25)	2.3 (±0.2)	2.15 (±0.2)	**3.96 (±0.26)**
1999	Sumatra	**7.83 (±0.35)**	3.4 (±0.24)	2.34 (±0.2)	2.09 (±0.19)	**4.23 (±0.27)**
2000	Sumatra	**7.64 (±0.35)**	3.17 (±0.24)	2.43 (±0.21)	2.04 (±0.19)	**4.42 (±0.27)**
2001	Sumatra	**7.47 (±0.34)**	2.85 (±0.22)	2.66 (±0.22)	1.96 (±0.19)	**4.59 (±0.28)**
2002	Sumatra	**7.19 (±0.34)**	2.6 (±0.22)	2.72 (±0.22)	1.87 (±0.18)	**4.87 (±0.29)**
2003	Sumatra	**7 (±0.34)**	2.43 (±0.21)	2.79 (±0.22)	1.77 (±0.18)	**5.06 (±0.29)**
2004	Sumatra	**6.59 (±0.33)**	2.19 (±0.2)	2.72 (±0.22)	1.68 (±0.17)	**5.47 (±0.3)**
2005	Sumatra	**6.17 (±0.32)**	2.04 (±0.19)	2.55 (±0.21)	1.59 (±0.17)	**5.89 (±0.31)**
2006	Sumatra	**5.81 (±0.31)**	1.94 (±0.19)	2.4 (±0.21)	1.47 (±0.16)	**6.25 (±0.32)**
2007	Sumatra	**5.53 (±0.3)**	1.94 (±0.19)	2.23 (±0.2)	1.36 (±0.16)	**6.53 (±0.33)**
2008	Sumatra	**5.23 (±0.3)**	1.85 (±0.18)	2.09 (±0.19)	1.28 (±0.15)	**6.83 (±0.33)**
2009	Sumatra	**5.02 (±0.29)**	1.77 (±0.18)	2 (±0.19)	1.25 (±0.15)	**7.04 (±0.34)**
2010	Sumatra	**4.81 (±0.29)**	1.66 (±0.17)	1.96 (±0.19)	1.19 (±0.15)	**7.25 (±0.34)**
2011	Sumatra	**4.59 (±0.28)**	1.6 (±0.17)	1.83 (±0.18)	1.15 (±0.15)	**7.47 (±0.34)**
2012	Sumatra	**4.25 (±0.27)**	1.53 (±0.17)	1.72 (±0.18)	1 (±0.14)	**7.81 (±0.35)**
2013	Sumatra	**4.15 (±0.27)**	1.47 (±0.16)	1.7 (±0.18)	0.98 (±0.13)	**7.91 (±0.35)**
2014	Sumatra	**3.81 (±0.26)**	1.43 (±0.16)	1.49 (±0.17)	0.89 (±0.13)	**8.25 (±0.36)**
2015	Sumatra	**3.6 (±0.25)**	1.4 (±0.16)	1.4 (±0.16)	0.81 (±0.12)	**8.45 (±0.36)**
2016	Sumatra	**3.45 (±0.25)**	1.26 (±0.15)	1.4 (±0.16)	0.79 (±0.12)	**8.61 (±0.36)**
2017	Sumatra	**3.42 (±0.24)**	1.21 (±0.15)	1.42 (±0.16)	0.79 (±0.12)	**8.64 (±0.37)**
2018	Sumatra	**3.36 (±0.24)**	1.17 (±0.15)	1.4 (±0.16)	0.79 (±0.12)	**8.7 (±0.37)**
2019	Sumatra	**3.32 (±0.24)**	1.15 (±0.15)	1.38 (±0.16)	0.79 (±0.12)	**8.74 (±0.37)**
2020	Sumatra	**3.28 (±0.24)**	1.09 (±0.14)	1.42 (±0.16)	0.77 (±0.12)	**8.78 (±0.37)**

Table S18. Estimated primary forest and forest loss area (Mha) in Papua in areas with slopes ≤4° and/or elevations ≤1500 meters. Standard errors are reported in parenthesis.

Year	Island	Primary forest	Intact	Degraded after 1990	Degraded in 1990	Forest loss
1990	Papua	**17.89 (±0.44)**	16.24 (±0.43)	–	1.65 (±0.17)	**–**
1991	Papua	**17.89 (±0.44)**	16.17 (±0.43)	0.08 (±0.04)	1.65 (±0.17)	**–**
1992	Papua	**17.85 (±0.44)**	16.09 (±0.43)	0.11 (±0.05)	1.65 (±0.17)	**0.04 (±0.03)**
1993	Papua	**17.81 (±0.44)**	15.83 (±0.43)	0.36 (±0.08)	1.63 (±0.17)	**0.08 (±0.04)**
1994	Papua	**17.78 (±0.44)**	15.66 (±0.43)	0.53 (±0.1)	1.59 (±0.17)	**0.11 (±0.05)**
1995	Papua	**17.76 (±0.44)**	15.49 (±0.43)	0.7 (±0.11)	1.57 (±0.17)	**0.13 (±0.05)**
1996	Papua	**17.74 (±0.44)**	15.3 (±0.43)	0.89 (±0.13)	1.55 (±0.17)	**0.15 (±0.05)**
1997	Papua	**17.55 (±0.44)**	14.28 (±0.42)	1.76 (±0.18)	1.51 (±0.17)	**0.34 (±0.08)**
1998	Papua	**17.47 (±0.44)**	13.63 (±0.42)	2.38 (±0.21)	1.46 (±0.16)	**0.42 (±0.09)**
1999	Papua	**17.36 (±0.44)**	13.28 (±0.41)	2.65 (±0.22)	1.44 (±0.16)	**0.53 (±0.1)**
2000	Papua	**17.34 (±0.44)**	13.11 (±0.41)	2.8 (±0.22)	1.44 (±0.16)	**0.55 (±0.1)**
2001	Papua	**17.28 (±0.44)**	12.9 (±0.41)	2.95 (±0.23)	1.44 (±0.16)	**0.61 (±0.11)**
2002	Papua	**17.28 (±0.44)**	12.67 (±0.41)	3.18 (±0.24)	1.44 (±0.16)	**0.61 (±0.11)**
2003	Papua	**17.27 (±0.44)**	12.42 (±0.4)	3.42 (±0.24)	1.42 (±0.16)	**0.62 (±0.11)**
2004	Papua	**17.25 (±0.44)**	12.27 (±0.4)	3.56 (±0.25)	1.42 (±0.16)	**0.64 (±0.11)**
2005	Papua	**17.25 (±0.44)**	12.25 (±0.4)	3.57 (±0.25)	1.42 (±0.16)	**0.64 (±0.11)**
2006	Papua	**17.23 (±0.44)**	12.12 (±0.4)	3.69 (±0.25)	1.42 (±0.16)	**0.66 (±0.11)**
2007	Papua	**17.21 (±0.43)**	12.05 (±0.4)	3.74 (±0.25)	1.42 (±0.16)	**0.68 (±0.11)**
2008	Papua	**17.21 (±0.43)**	11.86 (±0.4)	3.93 (±0.26)	1.42 (±0.16)	**0.68 (±0.11)**
2009	Papua	**17.15 (±0.43)**	11.71 (±0.4)	4.05 (±0.26)	1.4 (±0.16)	**0.74 (±0.12)**
2010	Papua	**17.13 (±0.43)**	11.52 (±0.4)	4.22 (±0.27)	1.4 (±0.16)	**0.76 (±0.12)**
2011	Papua	**17.04 (±0.43)**	11.46 (±0.4)	4.18 (±0.27)	1.4 (±0.16)	**0.85 (±0.13)**
2012	Papua	**16.96 (±0.43)**	11.35 (±0.39)	4.24 (±0.27)	1.38 (±0.16)	**0.93 (±0.13)**
2013	Papua	**16.91 (±0.43)**	11.23 (±0.39)	4.29 (±0.27)	1.38 (±0.16)	**0.98 (±0.13)**
2014	Papua	**16.83 (±0.43)**	11.03 (±0.39)	4.43 (±0.27)	1.38 (±0.16)	**1.06 (±0.14)**
2015	Papua	**16.77 (±0.43)**	10.85 (±0.39)	4.54 (±0.28)	1.38 (±0.16)	**1.12 (±0.14)**
2016	Papua	**16.72 (±0.43)**	10.74 (±0.39)	4.6 (±0.28)	1.38 (±0.16)	**1.17 (±0.15)**
2017	Papua	**16.72 (±0.43)**	10.68 (±0.39)	4.65 (±0.28)	1.38 (±0.16)	**1.17 (±0.15)**
2018	Papua	**16.7 (±0.43)**	10.65 (±0.39)	4.67 (±0.28)	1.38 (±0.16)	**1.19 (±0.15)**
2019	Papua	**16.68 (±0.43)**	10.57 (±0.39)	4.75 (±0.28)	1.36 (±0.16)	**1.21 (±0.15)**
2020	Papua	**16.68 (±0.43)**	10.46 (±0.38)	4.86 (±0.28)	1.36 (±0.16)	**1.21 (±0.15)**

Table S19. Estimated primary forest and forest loss area (Mha) in Sulawesi in areas with slopes ≤4° and/or elevations ≤1500 meters. Standard errors are reported in parenthesis.

Year	Island	Primary forest	Intact	Degraded after 1990	Degraded in 1990	Forest loss
1990	Sulawesi	**1.23 (±0.15)**	0.72 (±0.11)	–	0.51 (±0.1)	**–**
1991	Sulawesi	**1.23 (±0.15)**	0.72 (±0.11)	–	0.51 (±0.1)	**–**
1992	Sulawesi	**1.23 (±0.15)**	0.72 (±0.11)	–	0.51 (±0.1)	**–**
1993	Sulawesi	**1.21 (±0.15)**	0.7 (±0.11)	0.02 (±0.02)	0.49 (±0.09)	**0.02 (±0.02)**
1994	Sulawesi	**1.13 (±0.14)**	0.64 (±0.11)	0.06 (±0.03)	0.43 (±0.09)	**0.09 (±0.04)**
1995	Sulawesi	**1.11 (±0.14)**	0.6 (±0.11)	0.09 (±0.04)	0.42 (±0.09)	**0.11 (±0.05)**
1996	Sulawesi	**1.04 (±0.14)**	0.58 (±0.1)	0.08 (±0.04)	0.38 (±0.08)	**0.19 (±0.06)**
1997	Sulawesi	**1.02 (±0.13)**	0.51 (±0.1)	0.15 (±0.05)	0.36 (±0.08)	**0.21 (±0.06)**
1998	Sulawesi	**1 (±0.13)**	0.49 (±0.09)	0.17 (±0.06)	0.34 (±0.08)	**0.23 (±0.06)**
1999	Sulawesi	**0.96 (±0.13)**	0.47 (±0.09)	0.17 (±0.06)	0.32 (±0.08)	**0.26 (±0.07)**
2000	Sulawesi	**0.94 (±0.13)**	0.47 (±0.09)	0.17 (±0.06)	0.3 (±0.07)	**0.28 (±0.07)**
2001	Sulawesi	**0.92 (±0.13)**	0.45 (±0.09)	0.17 (±0.06)	0.3 (±0.07)	**0.3 (±0.07)**
2002	Sulawesi	**0.87 (±0.13)**	0.4 (±0.09)	0.21 (±0.06)	0.26 (±0.07)	**0.36 (±0.08)**
2003	Sulawesi	**0.79 (±0.12)**	0.4 (±0.09)	0.19 (±0.06)	0.21 (±0.06)	**0.43 (±0.09)**
2004	Sulawesi	**0.75 (±0.12)**	0.38 (±0.08)	0.19 (±0.06)	0.19 (±0.06)	**0.47 (±0.09)**
2005	Sulawesi	**0.72 (±0.11)**	0.38 (±0.08)	0.17 (±0.06)	0.17 (±0.06)	**0.51 (±0.1)**
2006	Sulawesi	**0.72 (±0.11)**	0.34 (±0.08)	0.21 (±0.06)	0.17 (±0.06)	**0.51 (±0.1)**
2007	Sulawesi	**0.72 (±0.11)**	0.32 (±0.08)	0.23 (±0.06)	0.17 (±0.06)	**0.51 (±0.1)**
2008	Sulawesi	**0.72 (±0.11)**	0.32 (±0.08)	0.23 (±0.06)	0.17 (±0.06)	**0.51 (±0.1)**
2009	Sulawesi	**0.66 (±0.11)**	0.32 (±0.08)	0.21 (±0.06)	0.13 (±0.05)	**0.57 (±0.1)**
2010	Sulawesi	**0.62 (±0.11)**	0.32 (±0.08)	0.21 (±0.06)	0.09 (±0.04)	**0.6 (±0.11)**
2011	Sulawesi	**0.6 (±0.11)**	0.32 (±0.08)	0.19 (±0.06)	0.09 (±0.04)	**0.62 (±0.11)**
2012	Sulawesi	**0.6 (±0.11)**	0.3 (±0.07)	0.21 (±0.06)	0.09 (±0.04)	**0.62 (±0.11)**
2013	Sulawesi	**0.6 (±0.11)**	0.3 (±0.07)	0.21 (±0.06)	0.09 (±0.04)	**0.62 (±0.11)**
2014	Sulawesi	**0.57 (±0.1)**	0.28 (±0.07)	0.21 (±0.06)	0.08 (±0.04)	**0.66 (±0.11)**
2015	Sulawesi	**0.53 (±0.1)**	0.26 (±0.07)	0.19 (±0.06)	0.08 (±0.04)	**0.7 (±0.11)**
2016	Sulawesi	**0.51 (±0.1)**	0.26 (±0.07)	0.17 (±0.06)	0.08 (±0.04)	**0.72 (±0.11)**
2017	Sulawesi	**0.51 (±0.1)**	0.26 (±0.07)	0.17 (±0.06)	0.08 (±0.04)	**0.72 (±0.11)**
2018	Sulawesi	**0.51 (±0.1)**	0.26 (±0.07)	0.17 (±0.06)	0.08 (±0.04)	**0.72 (±0.11)**
2019	Sulawesi	**0.51 (±0.1)**	0.26 (±0.07)	0.17 (±0.06)	0.08 (±0.04)	**0.72 (±0.11)**
2020	Sulawesi	**0.51 (±0.1)**	0.25 (±0.07)	0.19 (±0.06)	0.08 (±0.04)	**0.72 (±0.11)**

Table S20. Estimated primary forest and forest loss area (Mha) in Maluku, Java, and Nusa Tenggara in areas with slopes ≤4° and/or elevations ≤1500 meters. Standard errors are reported in parenthesis.

Year	Island	Primary forest	Intact	Degraded after 1990	Degraded in 1990	Forest loss
1990	Other	**1.61 (±0.16)**	1.02 (±0.13)	–	0.59 (±0.1)	**–**
1991	Other	**1.61 (±0.16)**	0.98 (±0.13)	0.04 (±0.03)	0.59 (±0.1)	**–**
1992	Other	**1.61 (±0.16)**	0.94 (±0.13)	0.08 (±0.04)	0.59 (±0.1)	**–**
1993	Other	**1.61 (±0.16)**	0.93 (±0.13)	0.09 (±0.04)	0.59 (±0.1)	**–**
1994	Other	**1.59 (±0.16)**	0.93 (±0.13)	0.08 (±0.04)	0.59 (±0.1)	**0.02 (±0.02)**
1995	Other	**1.57 (±0.16)**	0.93 (±0.13)	0.08 (±0.04)	0.57 (±0.1)	**0.04 (±0.03)**
1996	Other	**1.57 (±0.16)**	0.89 (±0.12)	0.11 (±0.05)	0.57 (±0.1)	**0.04 (±0.03)**
1997	Other	**1.55 (±0.16)**	0.83 (±0.12)	0.17 (±0.06)	0.55 (±0.1)	**0.06 (±0.03)**
1998	Other	**1.55 (±0.16)**	0.83 (±0.12)	0.17 (±0.06)	0.55 (±0.1)	**0.06 (±0.03)**
1999	Other	**1.53 (±0.16)**	0.81 (±0.12)	0.17 (±0.06)	0.55 (±0.1)	**0.08 (±0.04)**
2000	Other	**1.53 (±0.16)**	0.79 (±0.12)	0.19 (±0.06)	0.55 (±0.1)	**0.08 (±0.04)**
2001	Other	**1.53 (±0.16)**	0.79 (±0.12)	0.19 (±0.06)	0.55 (±0.1)	**0.08 (±0.04)**
2002	Other	**1.53 (±0.16)**	0.77 (±0.12)	0.21 (±0.06)	0.55 (±0.1)	**0.08 (±0.04)**
2003	Other	**1.53 (±0.16)**	0.76 (±0.11)	0.23 (±0.06)	0.55 (±0.1)	**0.08 (±0.04)**
2004	Other	**1.53 (±0.16)**	0.74 (±0.11)	0.25 (±0.07)	0.55 (±0.1)	**0.08 (±0.04)**
2005	Other	**1.51 (±0.16)**	0.74 (±0.11)	0.25 (±0.07)	0.53 (±0.1)	**0.09 (±0.04)**
2006	Other	**1.51 (±0.16)**	0.74 (±0.11)	0.25 (±0.07)	0.53 (±0.1)	**0.09 (±0.04)**
2007	Other	**1.51 (±0.16)**	0.74 (±0.11)	0.25 (±0.07)	0.53 (±0.1)	**0.09 (±0.04)**
2008	Other	**1.51 (±0.16)**	0.72 (±0.11)	0.26 (±0.07)	0.53 (±0.1)	**0.09 (±0.04)**
2009	Other	**1.48 (±0.15)**	0.7 (±0.11)	0.26 (±0.07)	0.51 (±0.1)	**0.13 (±0.05)**
2010	Other	**1.48 (±0.15)**	0.7 (±0.11)	0.26 (±0.07)	0.51 (±0.1)	**0.13 (±0.05)**
2011	Other	**1.48 (±0.15)**	0.7 (±0.11)	0.26 (±0.07)	0.51 (±0.1)	**0.13 (±0.05)**
2012	Other	**1.44 (±0.15)**	0.68 (±0.11)	0.26 (±0.07)	0.49 (±0.09)	**0.17 (±0.06)**
2013	Other	**1.44 (±0.15)**	0.68 (±0.11)	0.26 (±0.07)	0.49 (±0.09)	**0.17 (±0.06)**
2014	Other	**1.44 (±0.15)**	0.68 (±0.11)	0.26 (±0.07)	0.49 (±0.09)	**0.17 (±0.06)**
2015	Other	**1.4 (±0.15)**	0.66 (±0.11)	0.26 (±0.07)	0.47 (±0.09)	**0.21 (±0.06)**
2016	Other	**1.4 (±0.15)**	0.66 (±0.11)	0.26 (±0.07)	0.47 (±0.09)	**0.21 (±0.06)**
2017	Other	**1.38 (±0.15)**	0.66 (±0.11)	0.26 (±0.07)	0.45 (±0.09)	**0.23 (±0.06)**
2018	Other	**1.38 (±0.15)**	0.66 (±0.11)	0.26 (±0.07)	0.45 (±0.09)	**0.23 (±0.06)**
2019	Other	**1.36 (±0.15)**	0.66 (±0.11)	0.26 (±0.07)	0.44 (±0.09)	**0.25 (±0.07)**
2020	Other	**1.34 (±0.15)**	0.66 (±0.11)	0.26 (±0.07)	0.42 (±0.09)	**0.27 (±0.07)**

Table S21. Estimated annual primary forest loss and annual intact forest degradation (Mha). Estimated primary forest area actively cleared and cleared by fire is also reported. Standard errors are reported in parenthesis.

Year	Total loss	Active clearing	Cleared by fire	Intact forest degradation
1991	**0.57 (±0.1)**	0.36 (±0.08)	0.21 (±0.06)	**0.98 (±0.14)**
1992	**1.13 (±0.15)**	0.7 (±0.11)	0.43 (±0.09)	**1.53 (±0.17)**
1993	**0.87 (±0.13)**	0.7 (±0.11)	0.17 (±0.06)	**1.55 (±0.17)**
1994	**1.25 (±0.15)**	0.77 (±0.12)	0.47 (±0.09)	**1.7 (±0.18)**
1995	**1.17 (±0.15)**	0.87 (±0.13)	0.3 (±0.08)	**1.68 (±0.18)**
1996	**1.08 (±0.14)**	1.04 (±0.14)	0.04 (±0.03)	**1.45 (±0.16)**
1997	**2.51 (±0.22)**	0.89 (±0.13)	1.62 (±0.17)	**3.51 (±0.25)**
1998	**3.08 (±0.24)**	1.25 (±0.15)	1.83 (±0.18)	**3.55 (±0.26)**
1999	**1.15 (±0.15)**	1.04 (±0.14)	0.11 (±0.05)	**1.62 (±0.17)**
2000	**0.62 (±0.11)**	0.59 (±0.1)	0.04 (±0.03)	**1.42 (±0.16)**
2001	**0.68 (±0.11)**	0.62 (±0.11)	0.06 (±0.03)	**1.45 (±0.17)**
2002	**1 (±0.14)**	0.66 (±0.11)	0.34 (±0.08)	**1.28 (±0.16)**
2003	**0.64 (±0.11)**	0.47 (±0.09)	0.17 (±0.06)	**0.79 (±0.12)**
2004	**0.91 (±0.13)**	0.75 (±0.12)	0.15 (±0.05)	**1.17 (±0.15)**
2005	**0.96 (±0.13)**	0.89 (±0.13)	0.08 (±0.04)	**0.74 (±0.12)**
2006	**1.17 (±0.15)**	0.94 (±0.13)	0.23 (±0.07)	**0.83 (±0.12)**
2007	**0.79 (±0.12)**	0.74 (±0.12)	0.06 (±0.03)	**0.81 (±0.12)**
2008	**0.81 (±0.12)**	0.79 (±0.12)	0.02 (±0.02)	**0.81 (±0.12)**
2009	**1 (±0.14)**	0.81 (±0.12)	0.19 (±0.06)	**0.53 (±0.1)**
2010	**0.57 (±0.1)**	0.53 (±0.1)	0.04 (±0.03)	**0.62 (±0.11)**
2011	**0.81 (±0.12)**	0.81 (±0.12)	–	**0.38 (±0.08)**
2012	**0.83 (±0.12)**	0.83 (±0.12)	–	**0.51 (±0.1)**
2013	**0.59 (±0.1)**	0.53 (±0.1)	0.06 (±0.03)	**1 (±0.14)**
2014	**0.94 (±0.13)**	0.79 (±0.12)	0.15 (±0.05)	**0.85 (±0.13)**
2015	**1.04 (±0.14)**	0.6 (±0.11)	0.43 (±0.09)	**1.38 (±0.16)**
2016	**0.76 (±0.12)**	0.68 (±0.11)	0.08 (±0.04)	**0.87 (±0.13)**
2017	**0.4 (±0.09)**	0.4 (±0.09)	–	**0.76 (±0.12)**
2018	**0.32 (±0.08)**	0.32 (±0.08)	–	**0.55 (±0.1)**
2019	**0.42 (±0.09)**	0.34 (±0.08)	0.08 (±0.04)	**0.81 (±0.12)**
2020	**0.32 (±0.08)**	0.3 (±0.08)	0.02 (±0.02)	**0.44 (±0.09)**

Table S22. Estimated annual primary forest loss and annual intact forest degradation (Mha) in Kalimantan. Estimated primary forest area actively cleared and cleared by fire is also reported. Standard errors are reported in parenthesis.

Year	Island	Total loss	Active clearing	Cleared by fire	Intact forest degradation
1991	Kalimantan	**0.36 (±0.08)**	0.15 (±0.05)	0.21 (±0.06)	**0.42 (±0.09)**
1992	Kalimantan	**0.4 (±0.09)**	0.21 (±0.06)	0.19 (±0.06)	**0.53 (±0.1)**
1993	Kalimantan	**0.38 (±0.08)**	0.34 (±0.08)	0.04 (±0.03)	**0.7 (±0.11)**
1994	Kalimantan	**0.28 (±0.07)**	0.13 (±0.05)	0.15 (±0.05)	**0.58 (±0.1)**
1995	Kalimantan	**0.49 (±0.1)**	0.26 (±0.07)	0.23 (±0.07)	**0.81 (±0.12)**
1996	Kalimantan	**0.43 (±0.09)**	0.43 (±0.09)	–	**0.51 (±0.1)**
1997	Kalimantan	**1.47 (±0.16)**	0.36 (±0.08)	1.11 (±0.14)	**1.08 (±0.14)**
1998	Kalimantan	**1.57 (±0.17)**	0.4 (±0.09)	1.17 (±0.15)	**1.41 (±0.16)**
1999	Kalimantan	**0.34 (±0.08)**	0.32 (±0.08)	0.02 (±0.02)	**0.4 (±0.09)**
2000	Kalimantan	**0.23 (±0.07)**	0.23 (±0.07)	–	**0.64 (±0.11)**
2001	Kalimantan	**0.26 (±0.07)**	0.25 (±0.07)	0.02 (±0.02)	**0.53 (±0.1)**
2002	Kalimantan	**0.43 (±0.09)**	0.21 (±0.06)	0.23 (±0.07)	**0.4 (±0.09)**
2003	Kalimantan	**0.21 (±0.06)**	0.11 (±0.05)	0.09 (±0.04)	**0.13 (±0.05)**
2004	Kalimantan	**0.3 (±0.08)**	0.21 (±0.06)	0.09 (±0.04)	**0.38 (±0.08)**
2005	Kalimantan	**0.3 (±0.08)**	0.28 (±0.07)	0.02 (±0.02)	**0.3 (±0.08)**
2006	Kalimantan	**0.62 (±0.11)**	0.45 (±0.09)	0.17 (±0.06)	**0.28 (±0.07)**
2007	Kalimantan	**0.4 (±0.09)**	0.38 (±0.08)	0.02 (±0.02)	**0.19 (±0.06)**
2008	Kalimantan	**0.34 (±0.08)**	0.32 (±0.08)	0.02 (±0.02)	**0.19 (±0.06)**
2009	Kalimantan	**0.38 (±0.08)**	0.25 (±0.07)	0.13 (±0.05)	**0.08 (±0.04)**
2010	Kalimantan	**0.23 (±0.07)**	0.21 (±0.06)	0.02 (±0.02)	**0.13 (±0.05)**
2011	Kalimantan	**0.4 (±0.09)**	0.4 (±0.09)	–	**0.08 (±0.04)**
2012	Kalimantan	**0.28 (±0.07)**	0.28 (±0.07)	–	**0.11 (±0.05)**
2013	Kalimantan	**0.25 (±0.07)**	0.25 (±0.07)	–	**0.34 (±0.08)**
2014	Kalimantan	**0.3 (±0.08)**	0.26 (±0.07)	0.04 (±0.03)	**0.32 (±0.08)**
2015	Kalimantan	**0.51 (±0.1)**	0.26 (±0.07)	0.25 (±0.07)	**0.17 (±0.06)**
2016	Kalimantan	**0.32 (±0.08)**	0.32 (±0.08)	–	**0.17 (±0.06)**
2017	Kalimantan	**0.21 (±0.06)**	0.21 (±0.06)	–	**0.19 (±0.06)**
2018	Kalimantan	**0.09 (±0.04)**	0.09 (±0.04)	–	**0.23 (±0.07)**
2019	Kalimantan	**0.21 (±0.06)**	0.21 (±0.06)	–	**0.21 (±0.06)**
2020	Kalimantan	**0.13 (±0.05)**	0.13 (±0.05)	–	**0.04 (±0.03)**

Table S23. Estimated annual primary forest loss and annual intact forest degradation (Mha) in Sumatra. Estimated primary forest area actively cleared and cleared by fire is also reported. Standard errors are reported in parenthesis.

Year	Island	Total loss	Active clearing	Cleared by fire	Intact forest degradation
1991	Sumatra	**0.13 (±0.05)**	0.13 (±0.05)	–	**0.32 (±0.08)**
1992	Sumatra	**0.7 (±0.11)**	0.45 (±0.09)	0.25 (±0.07)	**0.77 (±0.12)**
1993	Sumatra	**0.4 (±0.09)**	0.32 (±0.08)	0.08 (±0.04)	**0.49 (±0.1)**
1994	Sumatra	**0.62 (±0.11)**	0.38 (±0.08)	0.25 (±0.07)	**0.64 (±0.11)**
1995	Sumatra	**0.51 (±0.1)**	0.43 (±0.09)	0.08 (±0.04)	**0.47 (±0.09)**
1996	Sumatra	**0.47 (±0.09)**	0.43 (±0.09)	0.04 (±0.03)	**0.49 (±0.1)**
1997	Sumatra	**0.62 (±0.11)**	0.34 (±0.08)	0.28 (±0.07)	**0.53 (±0.1)**
1998	Sumatra	**1.26 (±0.15)**	0.7 (±0.11)	0.57 (±0.1)	**1.11 (±0.14)**
1999	Sumatra	**0.36 (±0.08)**	0.34 (±0.08)	0.02 (±0.02)	**0.53 (±0.1)**
2000	Sumatra	**0.3 (±0.08)**	0.26 (±0.07)	0.04 (±0.03)	**0.4 (±0.09)**
2001	Sumatra	**0.25 (±0.07)**	0.23 (±0.07)	0.02 (±0.02)	**0.42 (±0.09)**
2002	Sumatra	**0.3 (±0.08)**	0.25 (±0.07)	0.06 (±0.03)	**0.26 (±0.07)**
2003	Sumatra	**0.23 (±0.07)**	0.19 (±0.06)	0.04 (±0.03)	**0.19 (±0.06)**
2004	Sumatra	**0.43 (±0.09)**	0.42 (±0.09)	0.02 (±0.02)	**0.28 (±0.07)**
2005	Sumatra	**0.53 (±0.1)**	0.49 (±0.1)	0.04 (±0.03)	**0.19 (±0.06)**
2006	Sumatra	**0.47 (±0.09)**	0.42 (±0.09)	0.06 (±0.03)	**0.15 (±0.05)**
2007	Sumatra	**0.32 (±0.08)**	0.28 (±0.07)	0.04 (±0.03)	**0.19 (±0.06)**
2008	Sumatra	**0.4 (±0.09)**	0.4 (±0.09)	–	**0.21 (±0.06)**
2009	Sumatra	**0.38 (±0.08)**	0.32 (±0.08)	0.06 (±0.03)	**0.13 (±0.05)**
2010	Sumatra	**0.25 (±0.07)**	0.25 (±0.07)	–	**0.09 (±0.04)**
2011	Sumatra	**0.28 (±0.07)**	0.28 (±0.07)	–	**0.11 (±0.05)**
2012	Sumatra	**0.42 (±0.09)**	0.42 (±0.09)	–	**0.09 (±0.04)**
2013	Sumatra	**0.21 (±0.06)**	0.17 (±0.06)	0.04 (±0.03)	**0.13 (±0.05)**
2014	Sumatra	**0.45 (±0.09)**	0.36 (±0.08)	0.09 (±0.04)	**0.04 (±0.03)**
2015	Sumatra	**0.23 (±0.07)**	0.13 (±0.05)	0.09 (±0.04)	**0.21 (±0.06)**
2016	Sumatra	**0.26 (±0.07)**	0.25 (±0.07)	0.02 (±0.02)	**0.25 (±0.07)**
2017	Sumatra	**0.09 (±0.04)**	0.09 (±0.04)	–	**0.13 (±0.05)**
2018	Sumatra	**0.15 (±0.05)**	0.15 (±0.05)	–	**0.13 (±0.05)**
2019	Sumatra	**0.09 (±0.04)**	0.06 (±0.03)	0.04 (±0.03)	**0.11 (±0.05)**
2020	Sumatra	**0.08 (±0.04)**	0.06 (±0.03)	0.02 (±0.02)	**0.08 (±0.04)**

Table S24. Estimated annual primary forest loss and annual intact forest degradation (Mha) in Papua. Estimated primary forest area actively cleared and cleared by fire is also reported. Standard errors are reported in parenthesis.

Year	Island	Total loss	Active clearing	Cleared by fire	Intact forest degradation
1991	Papua	**0.02 (±0.02)**	0.02 (±0.02)	–	**0.09 (±0.04)**
1992	Papua	**0.04 (±0.03)**	0.04 (±0.03)	–	**0.17 (±0.06)**
1993	Papua	**0.04 (±0.03)**	–	0.04 (±0.03)	**0.26 (±0.07)**
1994	Papua	**0.04 (±0.03)**	0.02 (±0.02)	0.02 (±0.02)	**0.26 (±0.07)**
1995	Papua	**0.02 (±0.02)**	0.02 (±0.02)	–	**0.25 (±0.07)**
1996	Papua	**0.04 (±0.03)**	0.04 (±0.03)	–	**0.26 (±0.07)**
1997	Papua	**0.25 (±0.07)**	0.08 (±0.04)	0.17 (±0.06)	**1.19 (±0.15)**
1998	Papua	**0.08 (±0.04)**	0.04 (±0.03)	0.04 (±0.03)	**0.76 (±0.12)**
1999	Papua	**0.19 (±0.06)**	0.13 (±0.05)	0.06 (±0.03)	**0.53 (±0.1)**
2000	Papua	**0.02 (±0.02)**	0.02 (±0.02)	–	**0.28 (±0.07)**
2001	Papua	**0.06 (±0.03)**	0.06 (±0.03)	–	**0.3 (±0.08)**
2002	Papua	**0.04 (±0.03)**	0.02 (±0.02)	0.02 (±0.02)	**0.38 (±0.08)**
2003	Papua	**0.04 (±0.03)**	0.04 (±0.03)	–	**0.3 (±0.08)**
2004	Papua	**0.04 (±0.03)**	0.04 (±0.03)	–	**0.28 (±0.07)**
2005	Papua	**–**	–	–	**0.13 (±0.05)**
2006	Papua	**0.04 (±0.03)**	0.04 (±0.03)	–	**0.17 (±0.06)**
2007	Papua	**0.04 (±0.03)**	0.04 (±0.03)	–	**0.21 (±0.06)**
2008	Papua	**–**	–	–	**0.28 (±0.07)**
2009	Papua	**0.09 (±0.04)**	0.09 (±0.04)	–	**0.25 (±0.07)**
2010	Papua	**0.04 (±0.03)**	0.02 (±0.02)	0.02 (±0.02)	**0.32 (±0.08)**
2011	Papua	**0.11 (±0.05)**	0.11 (±0.05)	–	**0.13 (±0.05)**
2012	Papua	**0.08 (±0.04)**	0.08 (±0.04)	–	**0.19 (±0.06)**
2013	Papua	**0.09 (±0.04)**	0.08 (±0.04)	0.02 (±0.02)	**0.28 (±0.07)**
2014	Papua	**0.08 (±0.04)**	0.08 (±0.04)	–	**0.42 (±0.09)**
2015	Papua	**0.09 (±0.04)**	0.06 (±0.03)	0.04 (±0.03)	**0.4 (±0.09)**
2016	Papua	**0.08 (±0.04)**	0.06 (±0.03)	0.02 (±0.02)	**0.28 (±0.07)**
2017	Papua	**0.02 (±0.02)**	0.02 (±0.02)	–	**0.3 (±0.08)**
2018	Papua	**0.02 (±0.02)**	0.02 (±0.02)	–	**0.11 (±0.05)**
2019	Papua	**0.04 (±0.03)**	0.02 (±0.02)	0.02 (±0.02)	**0.34 (±0.08)**
2020	Papua	**–**	–	–	**0.19 (±0.06)**

Table S25. Estimated annual primary forest loss and annual intact forest degradation (Mha) in Sulawesi. Estimated primary forest area actively cleared and cleared by fire is also reported. Standard errors are reported in parenthesis.

Year	Island	Total loss	Active clearing	Cleared by fire	Intact forest degradation
1991	Sulawesi	**0.02 (±0.02)**	0.02 (±0.02)	–	**0.02 (±0.02)**
1993	Sulawesi	**0.02 (±0.02)**	–	0.02 (±0.02)	**0.04 (±0.03)**
1994	Sulawesi	**0.26 (±0.07)**	0.23 (±0.06)	0.04 (±0.03)	**0.19 (±0.06)**
1995	Sulawesi	**0.06 (±0.03)**	0.06 (±0.03)	–	**0.13 (±0.05)**
1996	Sulawesi	**0.11 (±0.05)**	0.11 (±0.05)	–	**0.13 (±0.05)**
1997	Sulawesi	**0.09 (±0.04)**	0.09 (±0.04)	–	**0.47 (±0.09)**
1998	Sulawesi	**0.17 (±0.06)**	0.11 (±0.05)	0.06 (±0.03)	**0.21 (±0.06)**
1999	Sulawesi	**0.19 (±0.06)**	0.17 (±0.06)	0.02 (±0.02)	**0.13 (±0.05)**
2000	Sulawesi	**0.06 (±0.03)**	0.06 (±0.03)	–	**0.08 (±0.04)**
2001	Sulawesi	**0.09 (±0.04)**	0.08 (±0.04)	0.02 (±0.02)	**0.06 (±0.03)**
2002	Sulawesi	**0.19 (±0.06)**	0.17 (±0.06)	0.02 (±0.02)	**0.11 (±0.05)**
2003	Sulawesi	**0.11 (±0.05)**	0.09 (±0.04)	0.02 (±0.02)	**0.13 (±0.05)**
2004	Sulawesi	**0.11 (±0.05)**	0.09 (±0.04)	0.02 (±0.02)	**0.19 (±0.06)**
2005	Sulawesi	**0.09 (±0.04)**	0.08 (±0.04)	0.02 (±0.02)	**0.06 (±0.03)**
2006	Sulawesi	**0.02 (±0.02)**	0.02 (±0.02)	–	**0.17 (±0.06)**
2007	Sulawesi	**–**	–	–	**0.19 (±0.06)**
2008	Sulawesi	**0.08 (±0.04)**	0.08 (±0.04)	–	**0.08 (±0.04)**
2009	Sulawesi	**0.11 (±0.05)**	0.11 (±0.05)	–	**–**
2010	Sulawesi	**0.04 (±0.03)**	0.04 (±0.03)	–	**0.06 (±0.03)**
2011	Sulawesi	**0.02 (±0.02)**	0.02 (±0.02)	–	**0.04 (±0.03)**
2012	Sulawesi	**0.02 (±0.02)**	0.02 (±0.02)	–	**0.06 (±0.03)**
2013	Sulawesi	**0.04 (±0.03)**	0.04 (±0.03)	–	**0.19 (±0.06)**
2014	Sulawesi	**0.09 (±0.04)**	0.08 (±0.04)	0.02 (±0.02)	**0.06 (±0.03)**
2015	Sulawesi	**0.11 (±0.05)**	0.09 (±0.04)	0.02 (±0.02)	**0.43 (±0.09)**
2016	Sulawesi	**0.08 (±0.04)**	0.04 (±0.03)	0.04 (±0.03)	**0.09 (±0.04)**
2017	Sulawesi	**0.06 (±0.03)**	0.06 (±0.03)	–	**0.08 (±0.04)**
2018	Sulawesi	**0.04 (±0.03)**	0.04 (±0.03)	–	**0.06 (±0.03)**
2019	Sulawesi	**0.02 (±0.02)**	–	0.02 (±0.02)	**0.11 (±0.05)**
2020	Sulawesi	**0.04 (±0.03)**	0.04 (±0.03)	–	**0.08 (±0.04)**
1991	Sulawesi	**0.02 (±0.02)**	0.02 (±0.02)	–	**0.02 (±0.02)**

Table S26. Estimated annual primary forest loss and annual intact forest degradation (Mha) in Maluku, Java, and Nusa Tenggara. Estimated primary forest area actively cleared and cleared by fire is also reported. Standard errors are reported in parenthesis.

Year	Island	Total loss	Active clearing	Cleared by fire	Intact forest degradation
1991	Other	**0.04 (±0.03)**	0.04 (±0.03)	–	**0.13 (±0.05)**
1992	Other	**–**	–	–	**0.06 (±0.03)**
1993	Other	**0.04 (±0.03)**	0.04 (±0.03)	–	**0.06 (±0.03)**
1994	Other	**0.04 (±0.03)**	0.02 (±0.02)	0.02 (±0.02)	**0.02 (±0.02)**
1995	Other	**0.09 (±0.04)**	0.09 (±0.04)	–	**0.02 (±0.02)**
1996	Other	**0.02 (±0.02)**	0.02 (±0.02)	–	**0.06 (±0.03)**
1997	Other	**0.08 (±0.04)**	0.02 (±0.02)	0.06 (±0.03)	**0.25 (±0.07)**
1998	Other	**–**	–	–	**0.06 (±0.03)**
1999	Other	**0.08 (±0.04)**	0.08 (±0.04)	–	**0.04 (±0.03)**
2000	Other	**0.02 (±0.02)**	0.02 (±0.02)	–	**0.02 (±0.02)**
2001	Other	**0.02 (±0.02)**	0.02 (±0.02)	–	**0.15 (±0.05)**
2002	Other	**0.04 (±0.03)**	0.02 (±0.02)	0.02 (±0.02)	**0.13 (±0.05)**
2003	Other	**0.06 (±0.03)**	0.04 (±0.03)	0.02 (±0.02)	**0.04 (±0.03)**
2004	Other	**0.02 (±0.02)**	–	0.02 (±0.02)	**0.04 (±0.03)**
2005	Other	**0.04 (±0.03)**	0.04 (±0.03)	–	**0.06 (±0.03)**
2006	Other	**0.02 (±0.02)**	0.02 (±0.02)	–	**0.06 (±0.03)**
2007	Other	**0.04 (±0.03)**	0.04 (±0.03)	–	**0.04 (±0.03)**
2008	Other	**–**	–	–	**0.06 (±0.03)**
2009	Other	**0.04 (±0.03)**	0.04 (±0.03)	–	**0.08 (±0.04)**
2010	Other	**0.02 (±0.02)**	0.02 (±0.02)	–	**0.02 (±0.02)**
2011	Other	**–**	–	–	**0.02 (±0.02)**
2012	Other	**0.04 (±0.03)**	0.04 (±0.03)	–	**0.06 (±0.03)**
2013	Other	**–**	–	–	**0.06 (±0.03)**
2014	Other	**0.02 (±0.02)**	0.02 (±0.02)	–	**0.02 (±0.02)**
2015	Other	**0.09 (±0.04)**	0.06 (±0.03)	0.04 (±0.03)	**0.17 (±0.06)**
2016	Other	**0.02 (±0.02)**	0.02 (±0.02)	–	**0.08 (±0.04)**
2017	Other	**0.02 (±0.02)**	0.02 (±0.02)	–	**0.06 (±0.03)**
2018	Other	**0.02 (±0.02)**	0.02 (±0.02)	–	**0.02 (±0.02)**
2019	Other	**0.06 (±0.03)**	0.06 (±0.03)	–	**0.04 (±0.03)**
2020	Other	**0.08 (±0.04)**	0.08 (±0.04)	–	**0.06 (±0.03)**
